# Supplementary material for: Synthesis of Tetrasubstituted Phosphorus Analogs of Aspartic Acid as Antiproliferative Agents
Source: Molecules. 2022 Nov 18;27(22):8024. doi: 10.3390/molecules27228024 (PMC9693455; doi:10.3390/molecules27228024)

Supporting Information

Synthesis of Tetrasubstituted Phosphorus Analogs of Aspartic Acid as Antiproliferative Agents

Xabier del Corte †, Aitor Maestro †, Adrián López-Francés, Francisco Palacios and Javier Vicario *

Departamento de Química Orgánica I, Centro de Investigación y Estudios Avanzados "Lucio Lascaray", Facultad de Farmacia, University of the Basque Country, UPV/EHU Paseo de la Universidad 7, 01006 Vitoria-Gasteiz, Spain
* Correspondence: javier.vicario@ehu.eus; Tel.: +34 945013891.
† These authors contributed equally to this work.

1. ^{1}H NMR and ^{13}C NMR spectra of compounds **5** and **6** **S2**

2. ^{1}H NMR, ^{13}C NMR, ^{31}P NMR and ^{19}F NMR spectra of compounds **7** **S4**

3. ^{1}H NMR, ^{13}C NMR, ^{31}P NMR and ^{19}F NMR spectra of compounds **12**, **13** and **14** **S42**

4. ^{1}H NMR, ^{13}C NMR and ^{31}P NMR spectra of compound **18** **S50**

5. 2D NMR spectra of compound **7a** **S52**

1. ^{1}H NMR and ^{13}C NMR spectra of compounds 5 and 6

Ethyl 3-((4-methylphenyl)sulfonamido)-3-phenylpropanoate (5).

^{1}H NMR (400 MHz, $CDCl_3$)

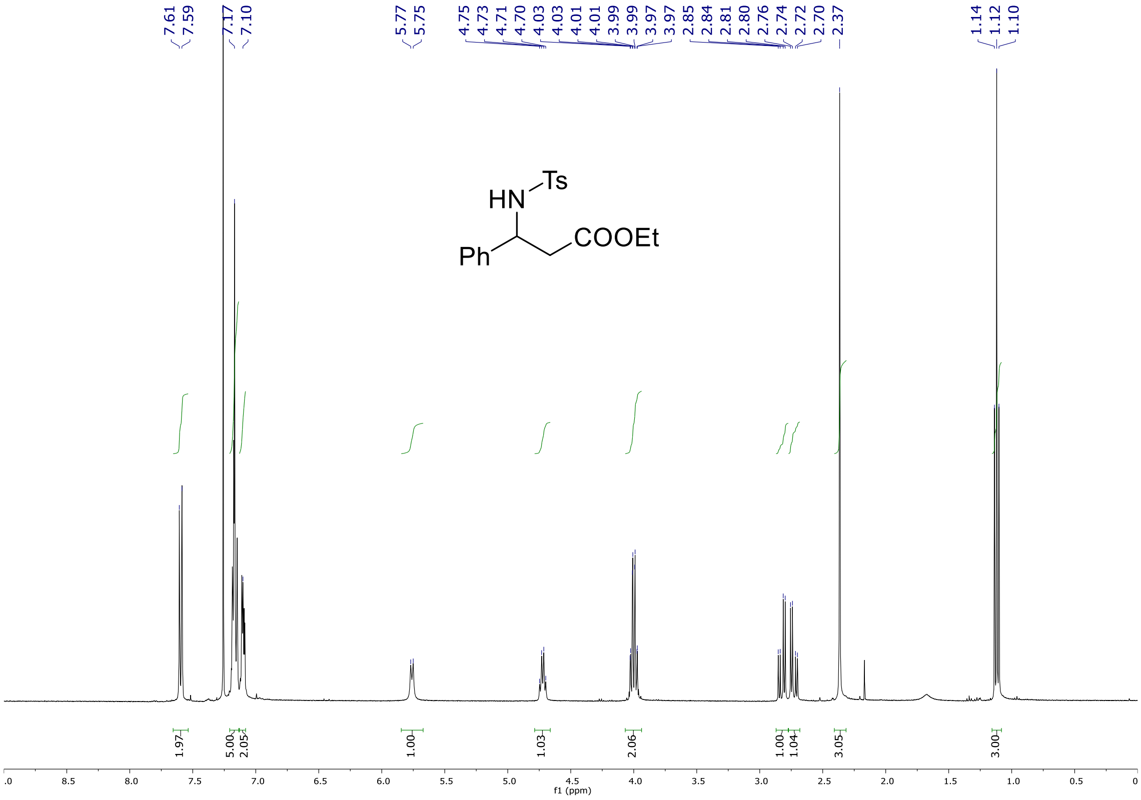

^{13}C NMR {^{1}H} (101 MHz, $CDCl_3$)

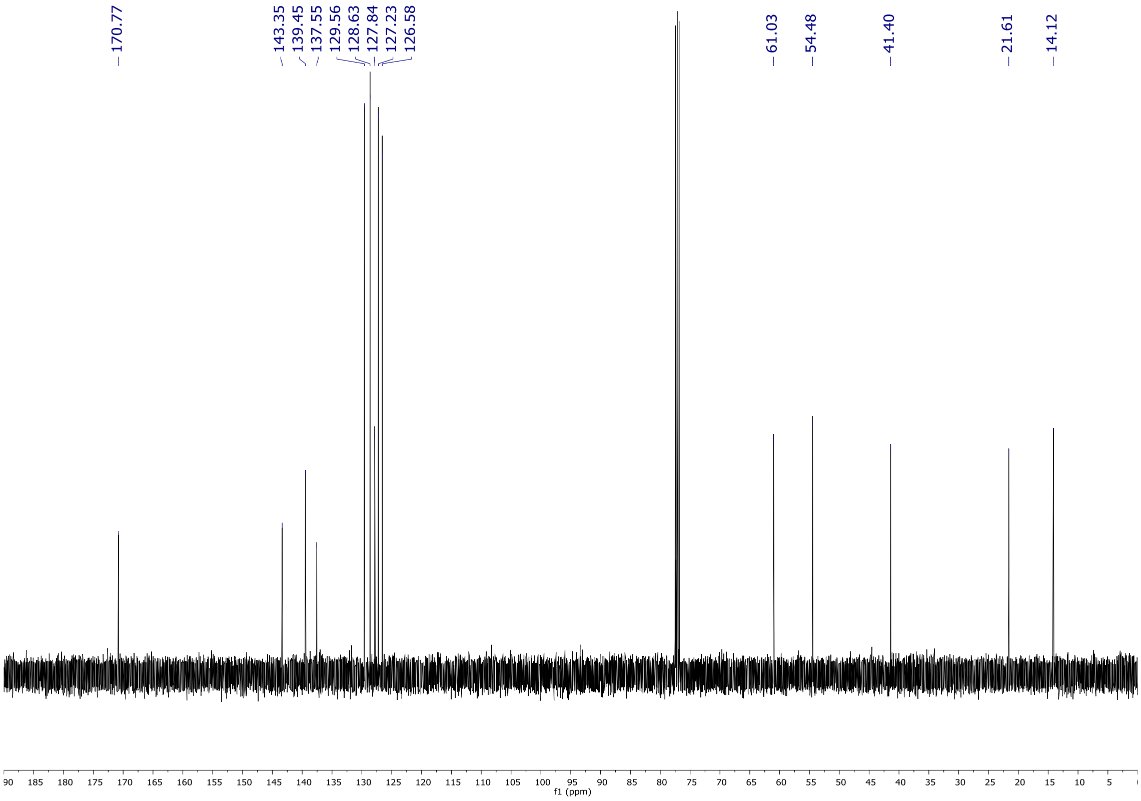

Diethyl 2-((4-methylphenyl)sulfonamido)-2-phenylsuccinate (6).

^{1}H NMR (400 MHz, $CDCl_3$)

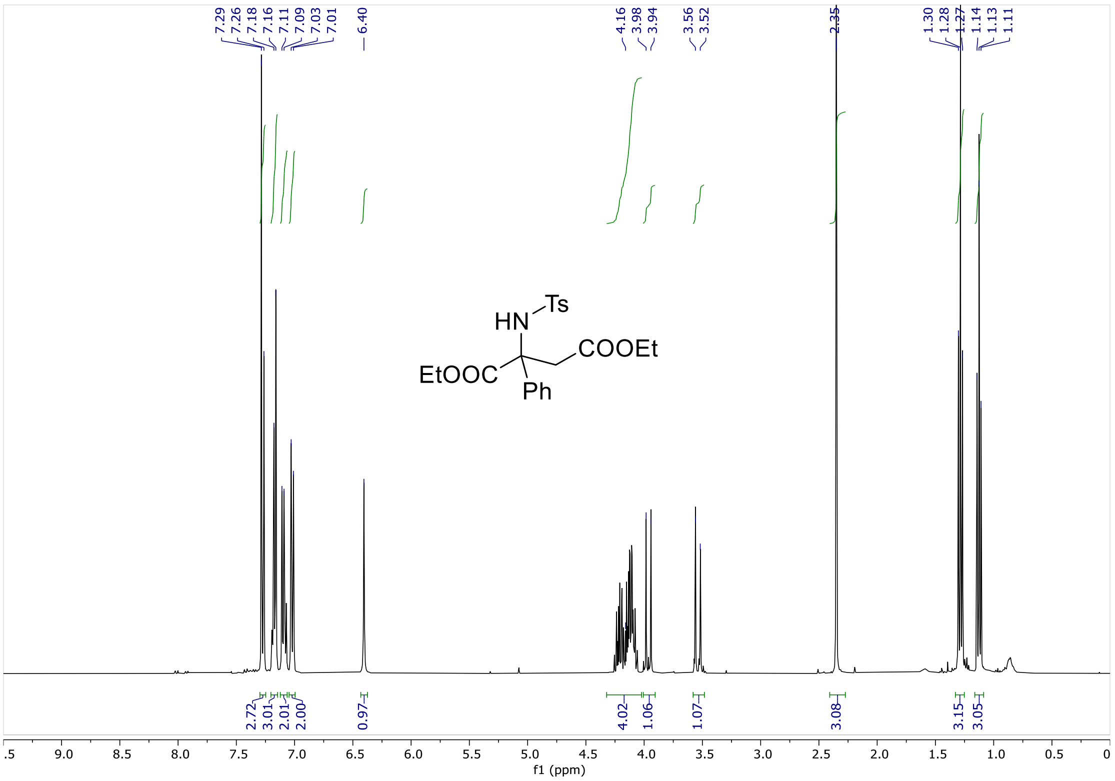

^{13}C NMR {^{1}H} (101 MHz, $CDCl_3$)

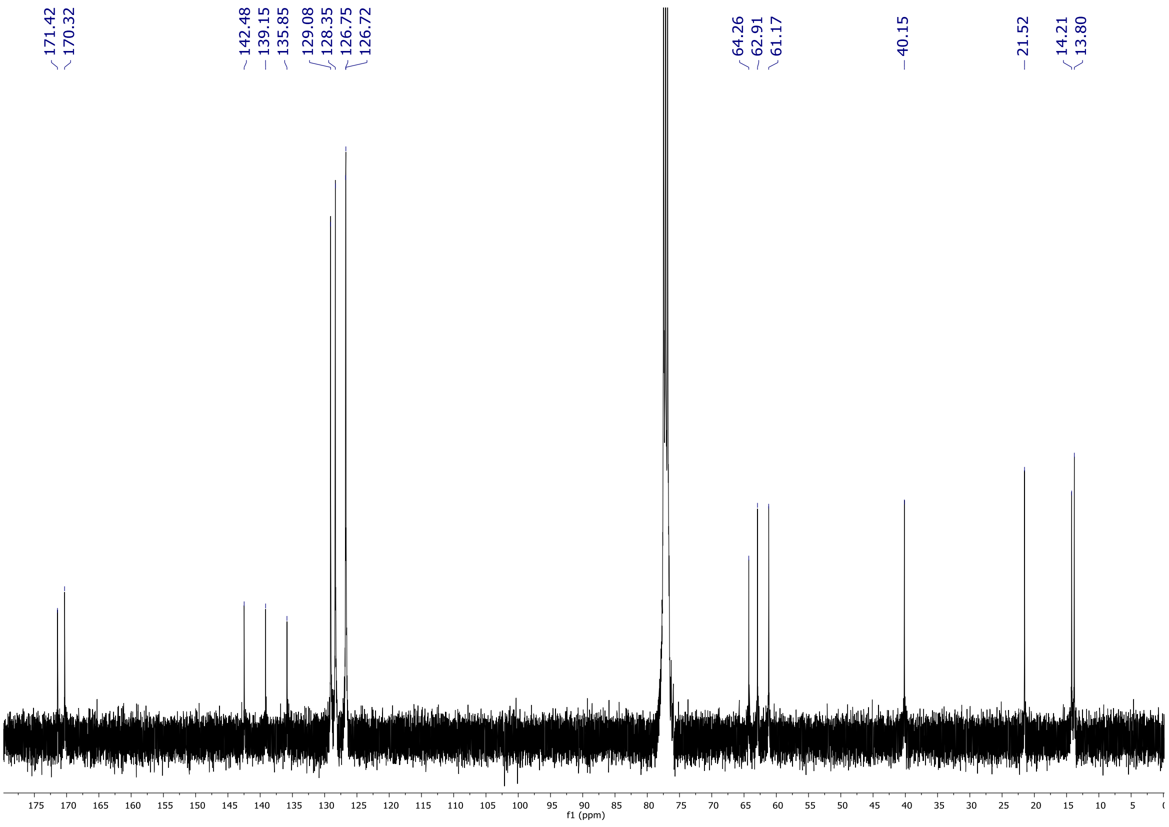

2. ^{1}H NMR, ^{13}C NMR, ^{31}P NMR and ^{19}FNMR spectra of compounds 7

Ethyl 3-(dimethoxyphosphoryl)-3-((4-methylphenyl)sulfonamido)-3-phenylpropanoate (7a)

^{1}H NMR (400 MHz, $CDCl_3$)

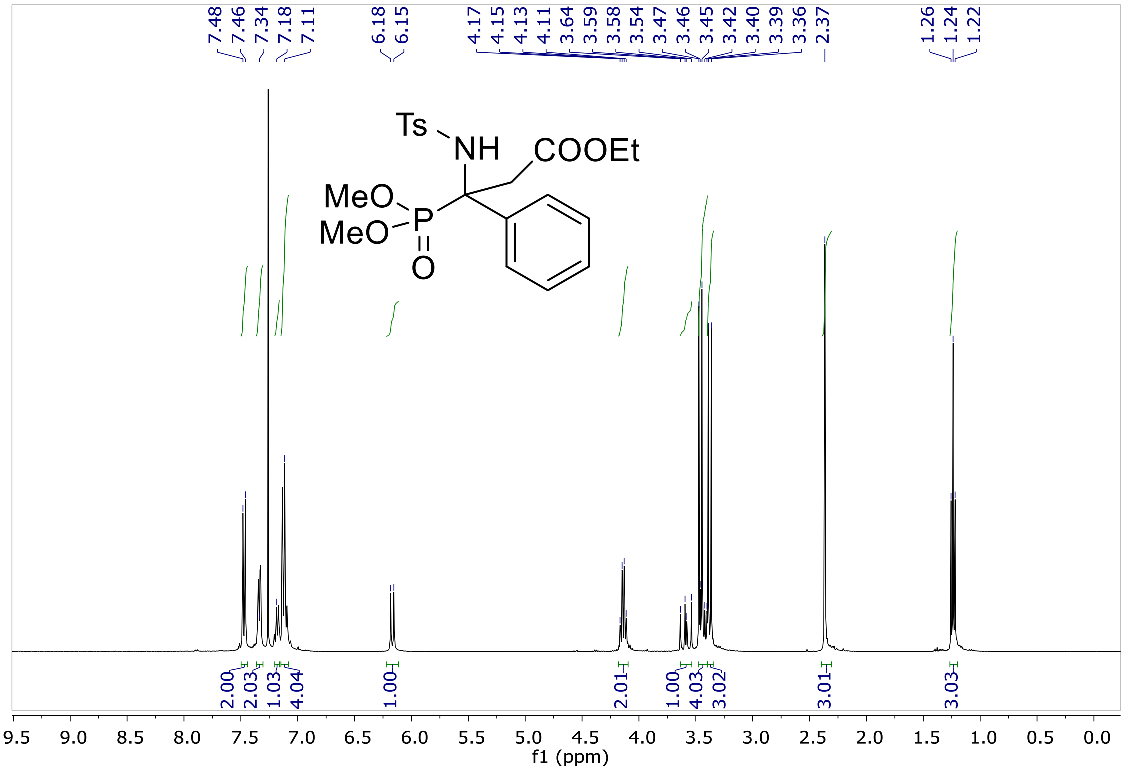

^{13}C NMR {^{1}H} (101 MHz, $CDCl_3$)

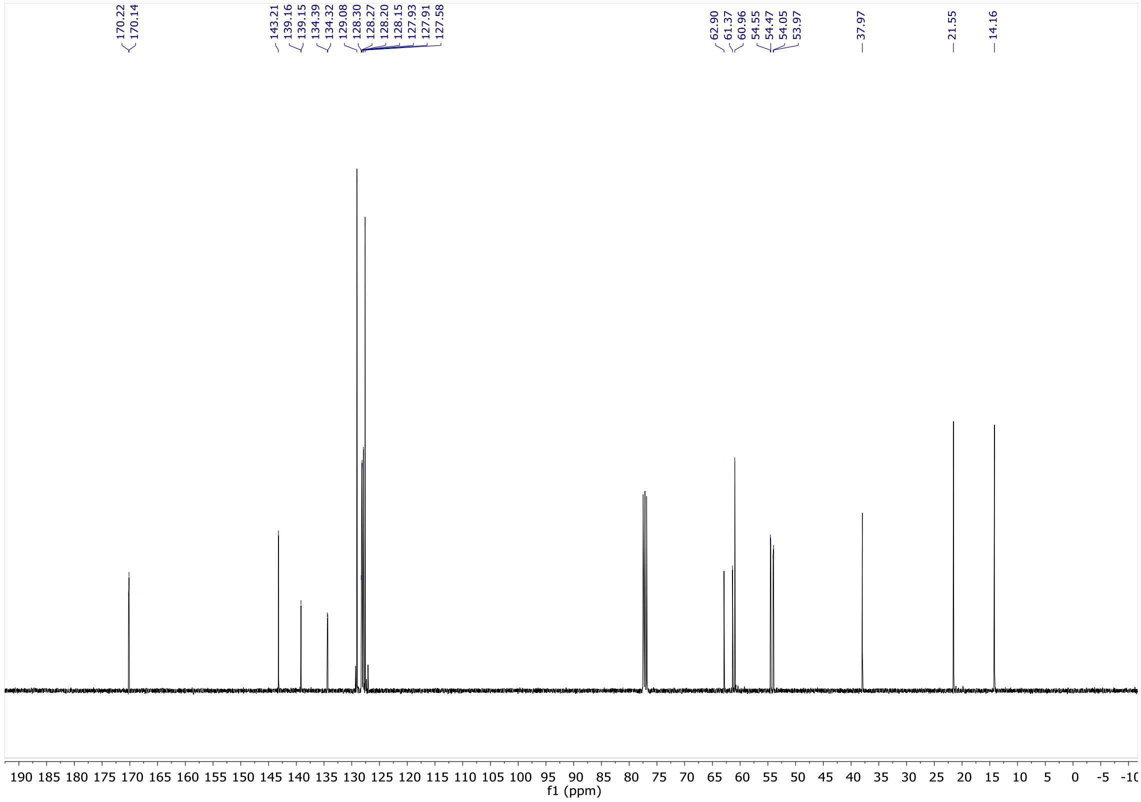

^{31}P NMR (120 MHz, $CDCl_3$)

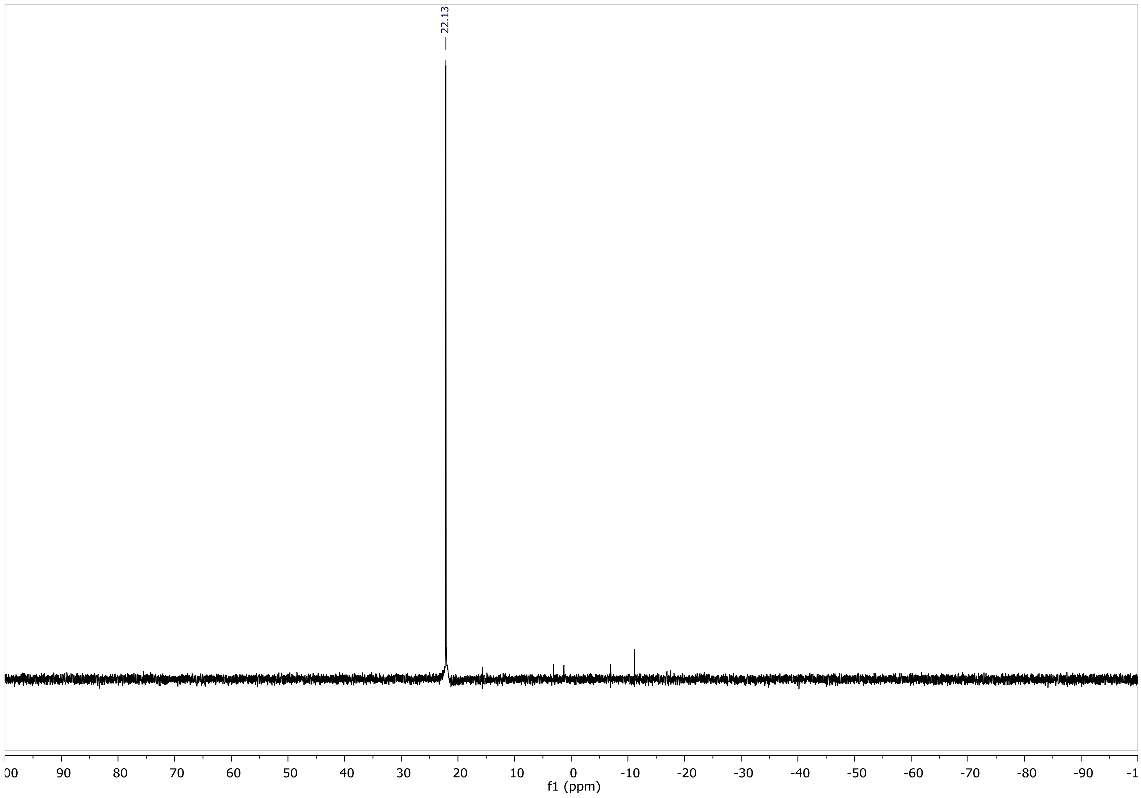

Ethyl 3-(dimethoxyphosphoryl)-3-((4-methylphenyl)sulfonamido)-3-(p-tolyl)propanoate (7b)

^{1}H NMR (400 MHz, $CDCl_3$)

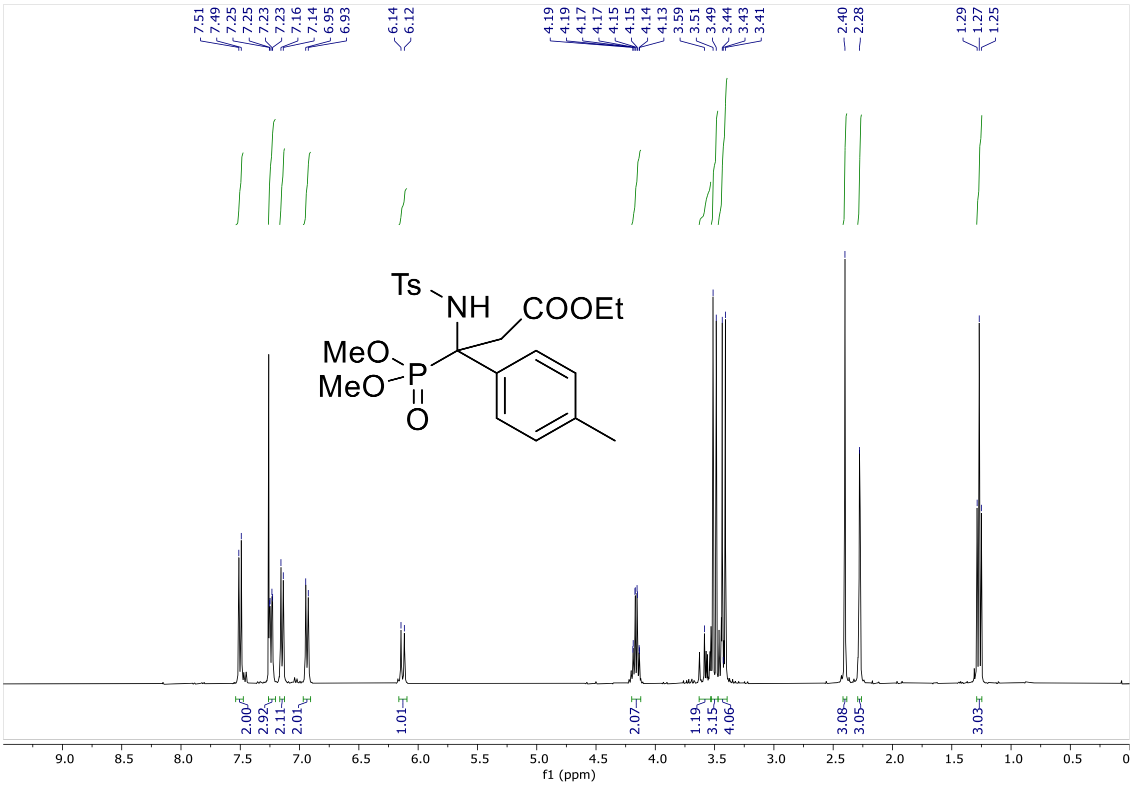

^{13}C NMR {^{1}H} (75 MHz, $CDCl_3$)

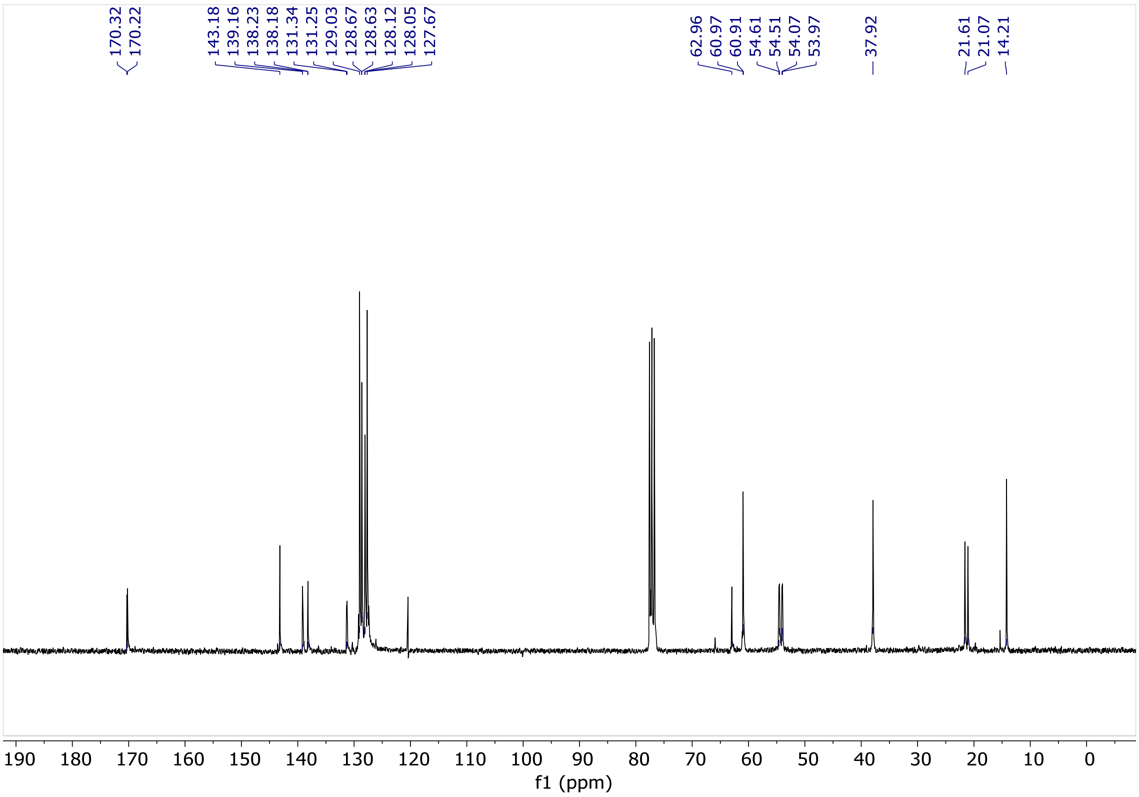

^{31}P NMR (120 MHz, $CDCl_3$)

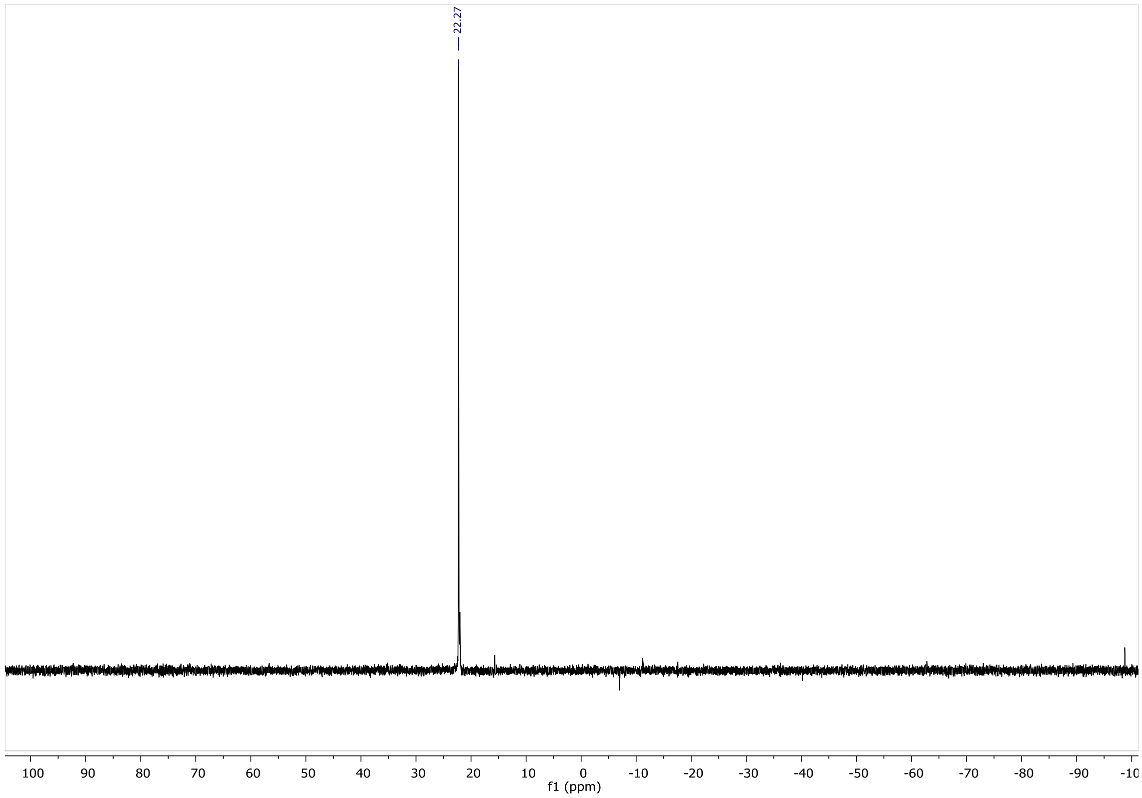

Ethyl 3-(dimethoxyphosphoryl)-3-((4-methylphenyl)sulfonamido)-3-(m-tolyl)propanoate (7c)

^{1}H NMR (400 MHz, $CDCl_3$)

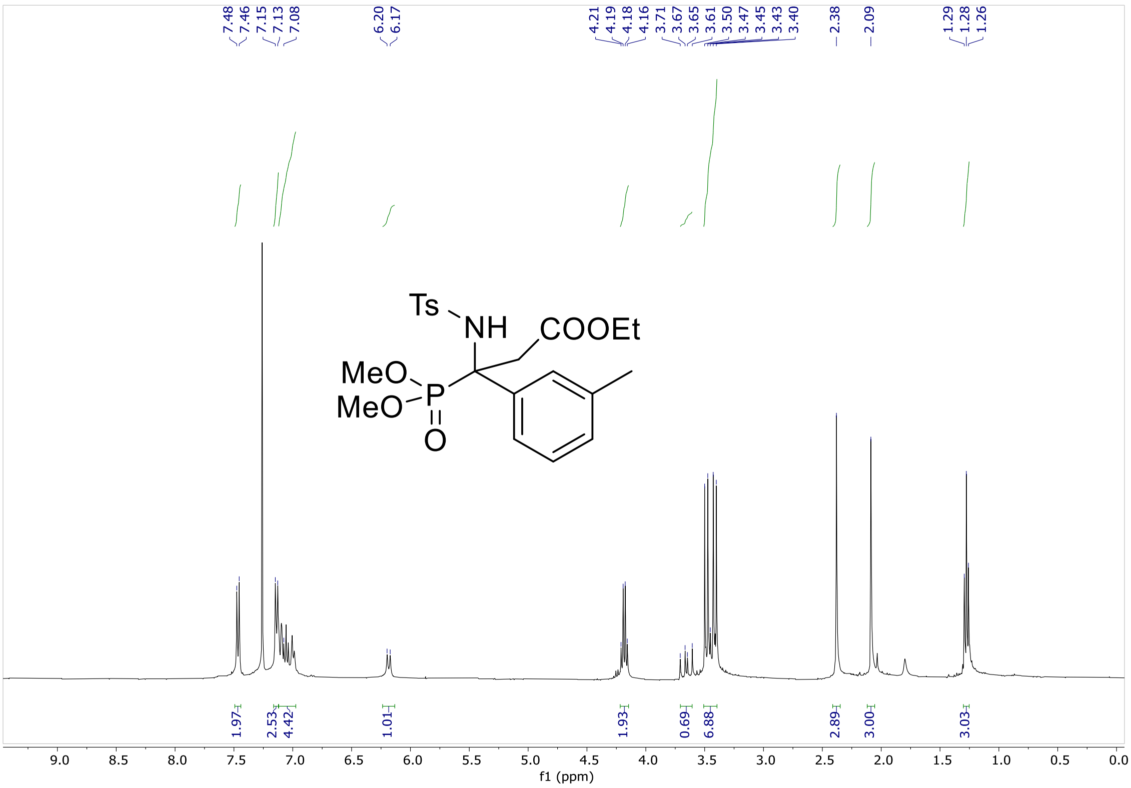

^{13}C NMR {^{1}H} (101 MHz, $CDCl_3$)

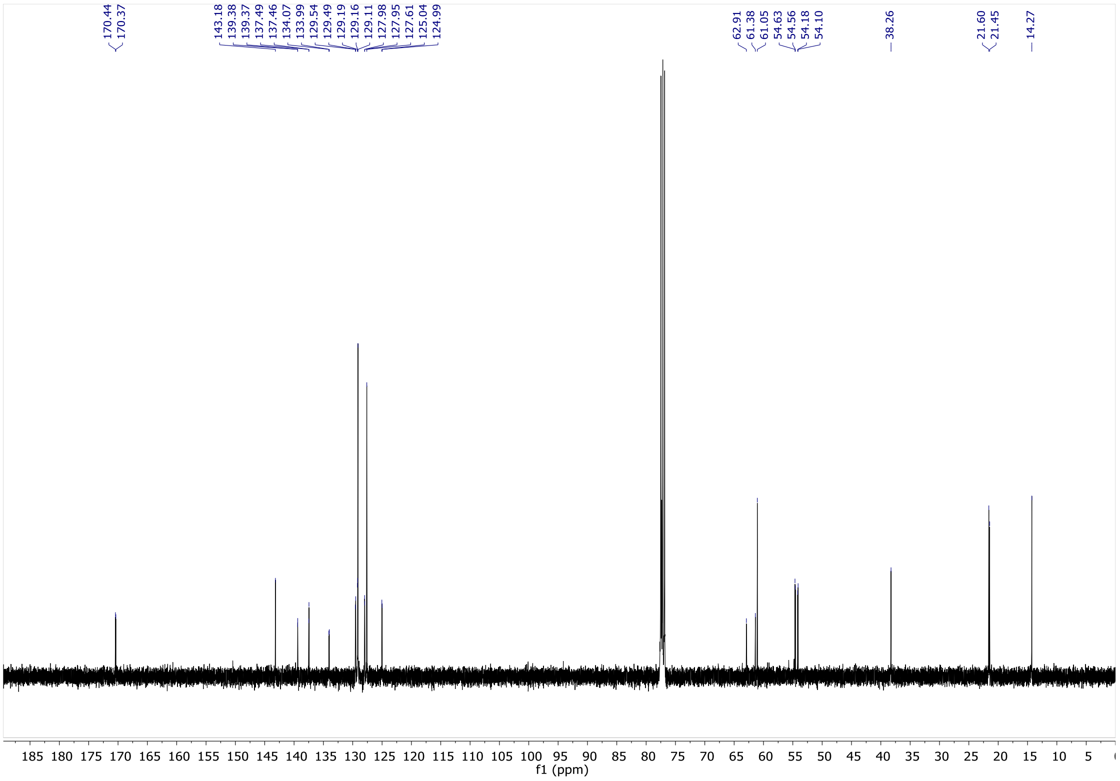

^{31}P NMR (120 MHz, $CDCl_3$)

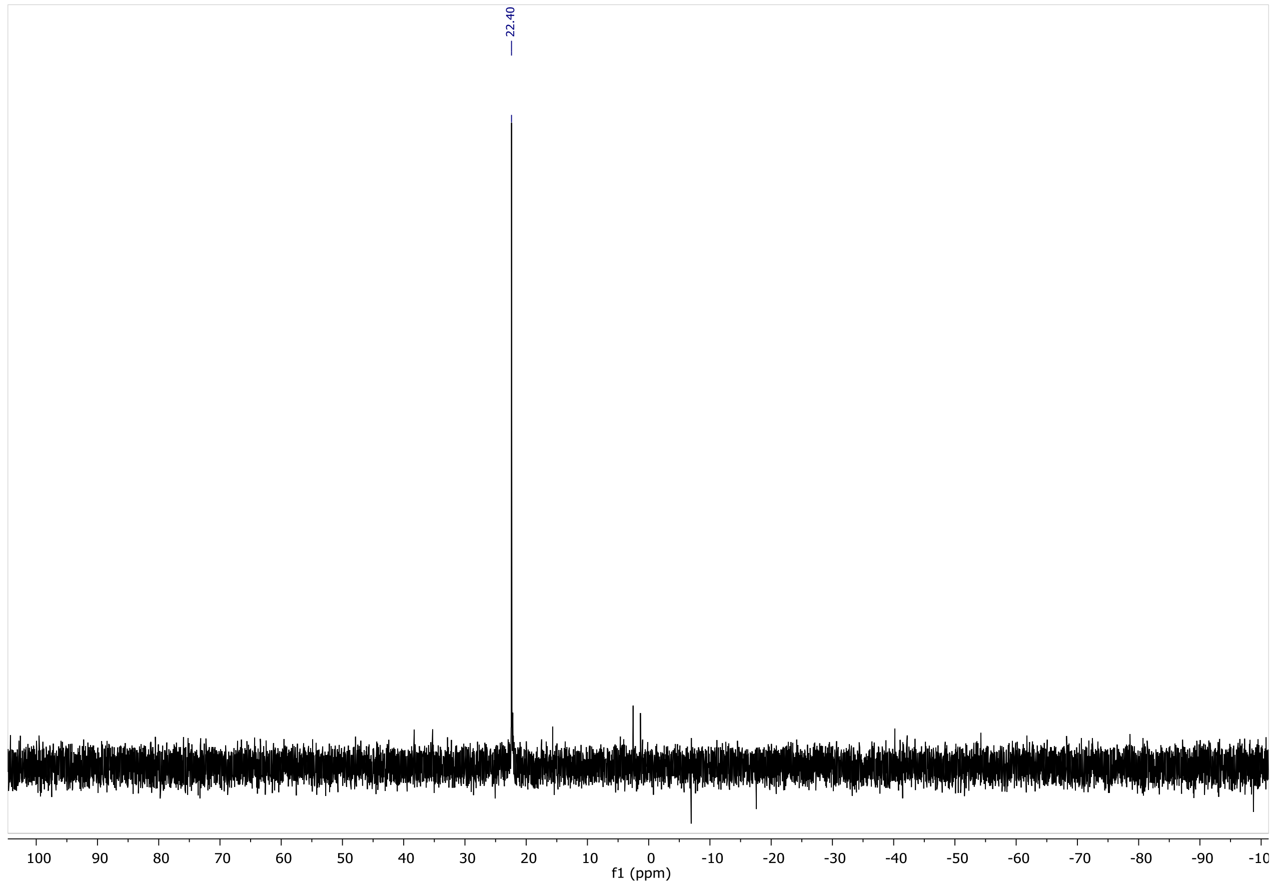

Ethyl 3-(dimethoxyphosphoryl)-3-((4-methylphenyl)sulfonamido)-3-(4-((trichloromethyl)thio) phenyl)propanoate (7d)

^{1}H NMR (400 MHz, $CDCl_3$)

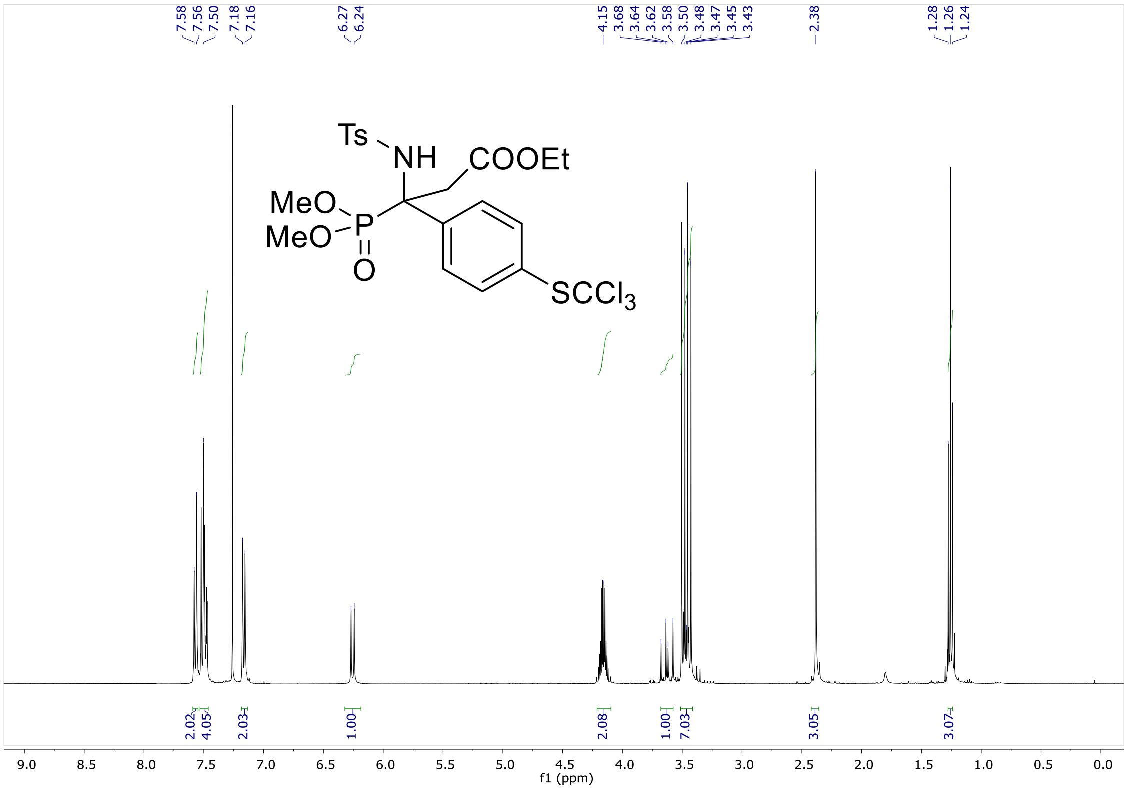

^{13}C NMR {^{1}H} (101 MHz, $CDCl_3$)

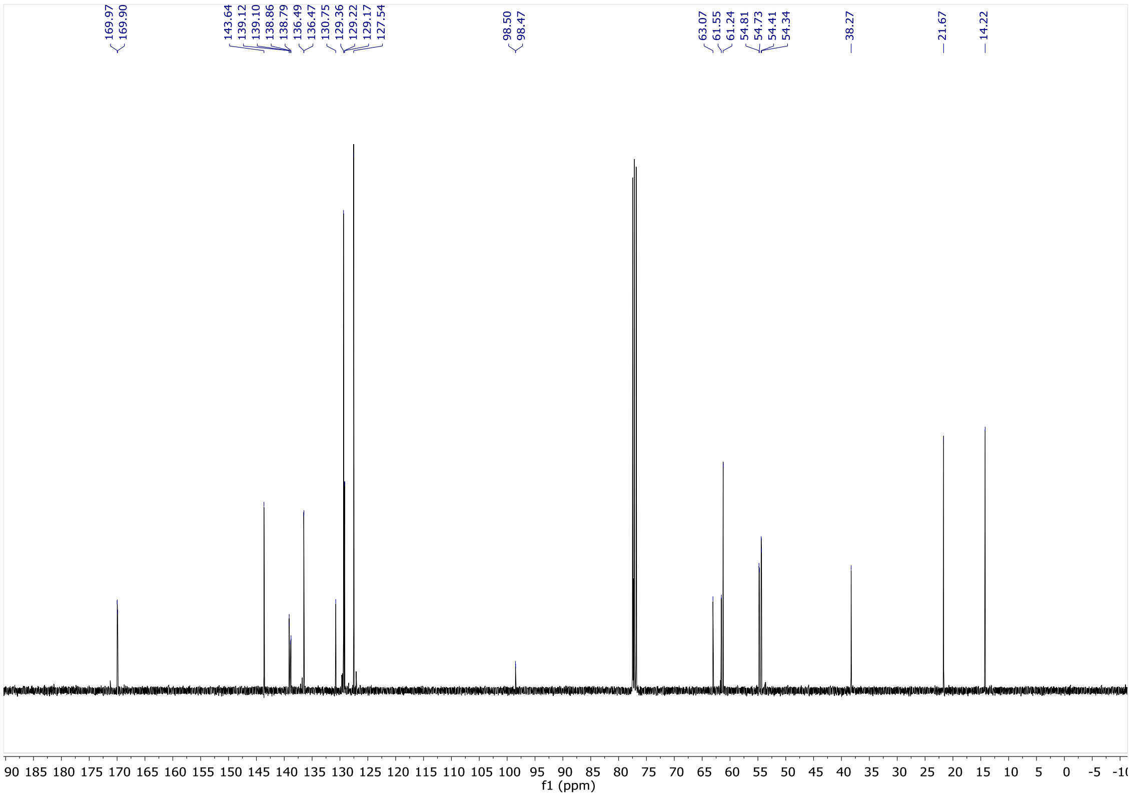

^{31}P NMR (120 MHz, $CDCl_3$)

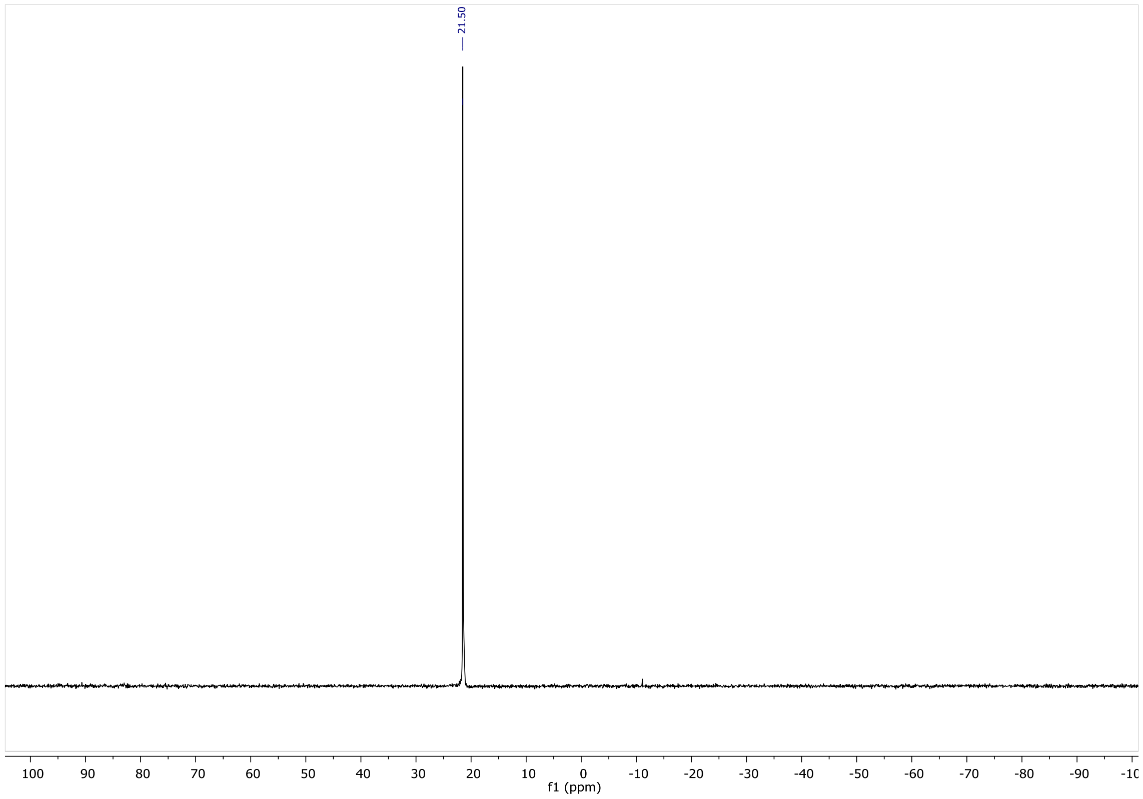

Ethyl 3-(4-bromophenyl)-3-(dimethoxyphosphoryl)-3-((4-methylphenyl)sulfonamido) propanoate (7e)

^{1}H NMR (400 MHz, $CDCl_3$)

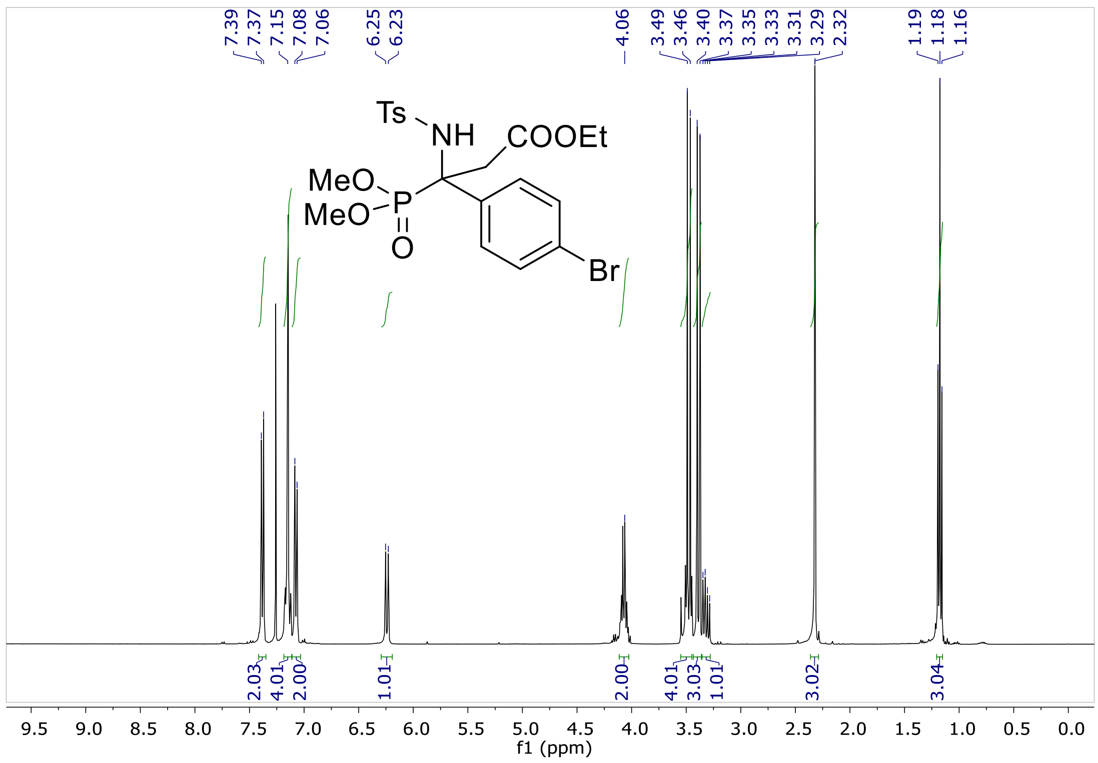

^{13}C NMR {^{1}H} (101 MHz, $CDCl_3$)

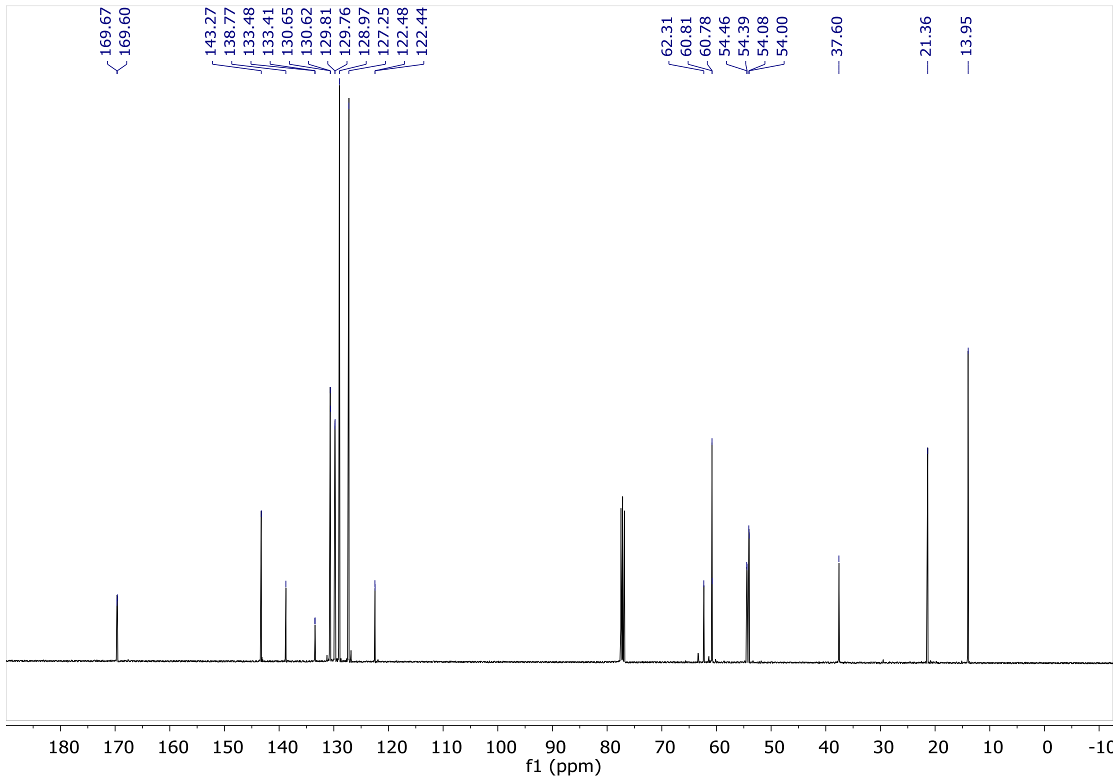

^{31}P NMR (120 MHz, $CDCl_3$)

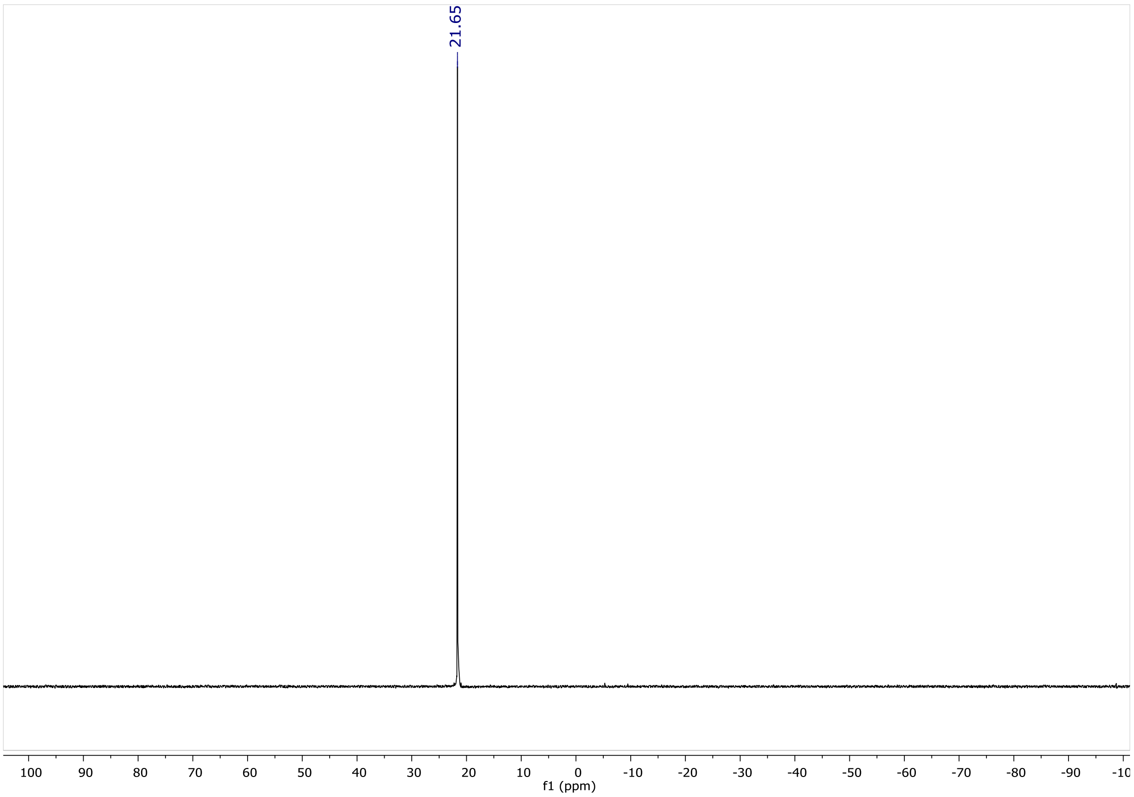

Ethyl 3-(4-chlorophenyl)-3-(dimethoxyphosphoryl)-3-((4-methylphenyl)sulfonamido) propanoate (7f)

^{1}H NMR (400 MHz, $CDCl_3$)

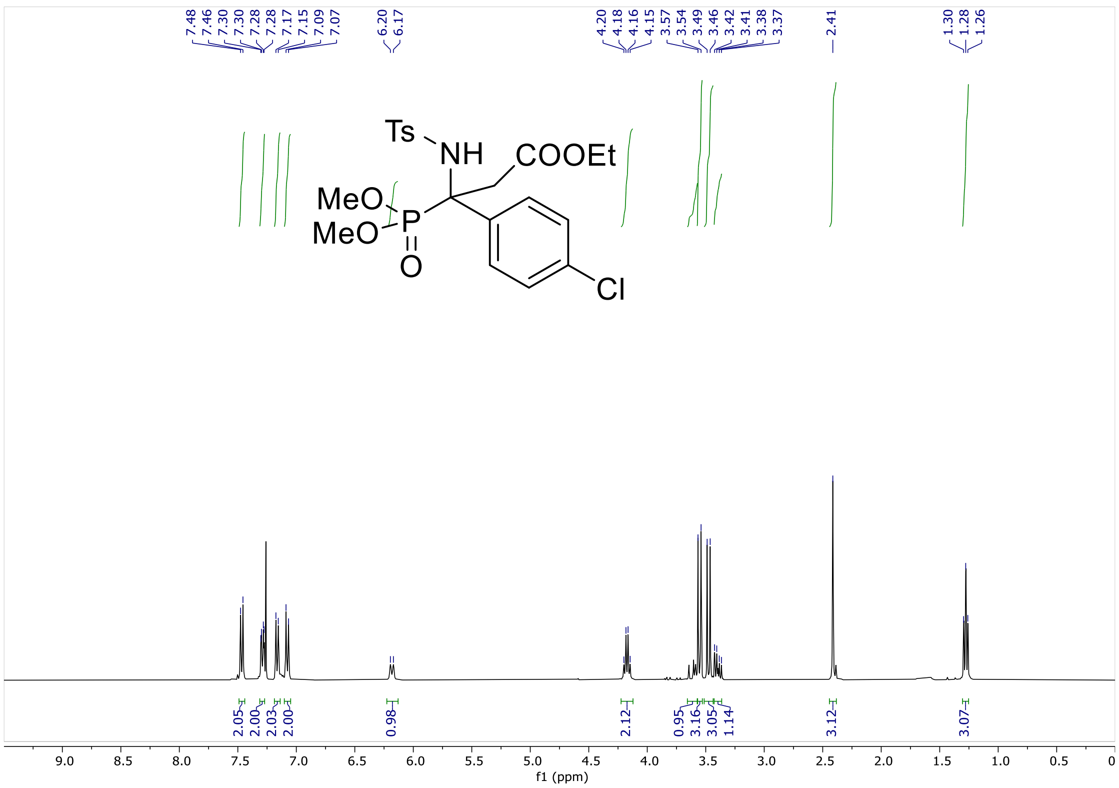

^{13}C NMR {^{1}H} (75 MHz, $CDCl_3$)

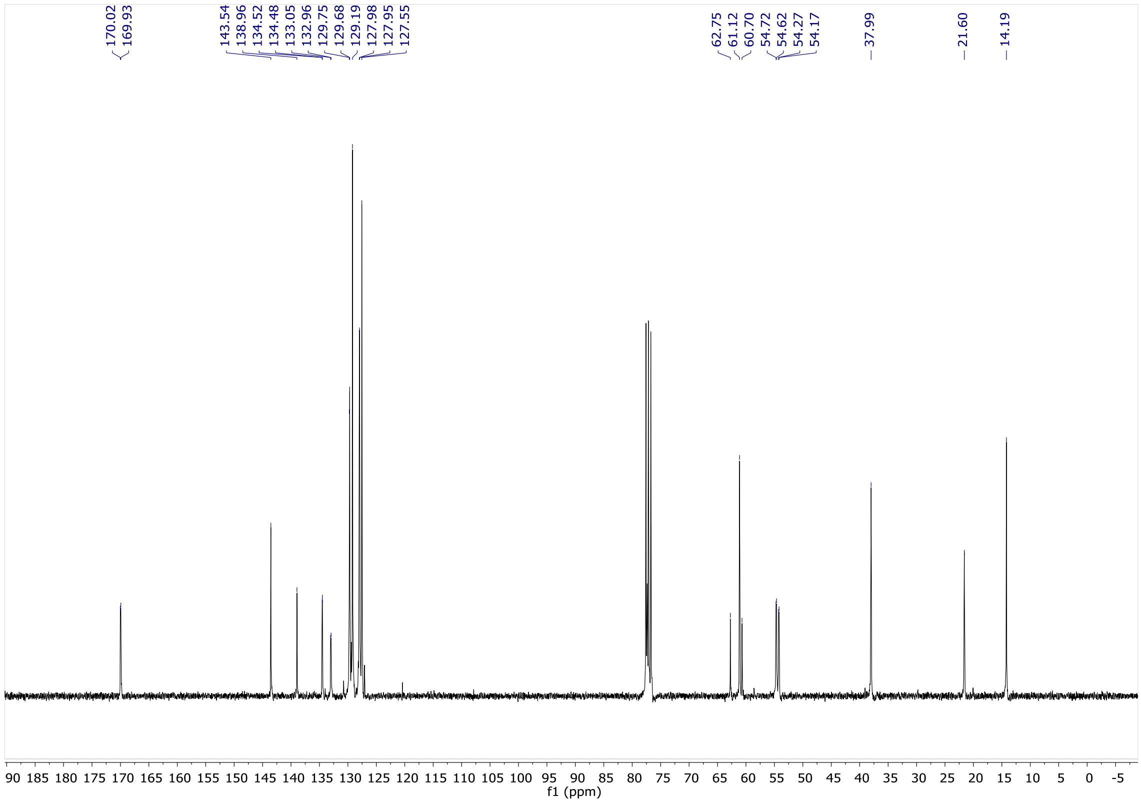

^{31}P NMR (120 MHz, $CDCl_3$)

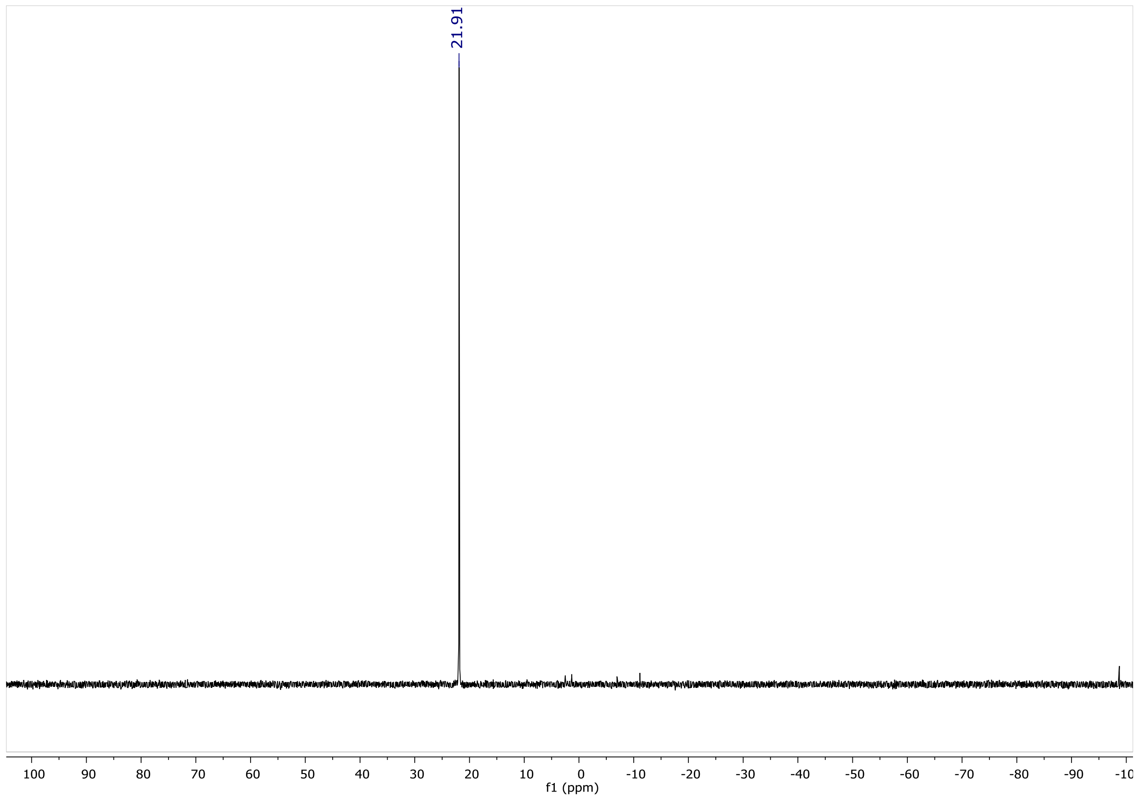

Ethyl 3-(3-chlorophenyl)-3-(dimethoxyphosphoryl)-3-((4-methylphenyl)sulfonamido) propanoate (7g)

^{1}H NMR (400 MHz, $CDCl_3$)

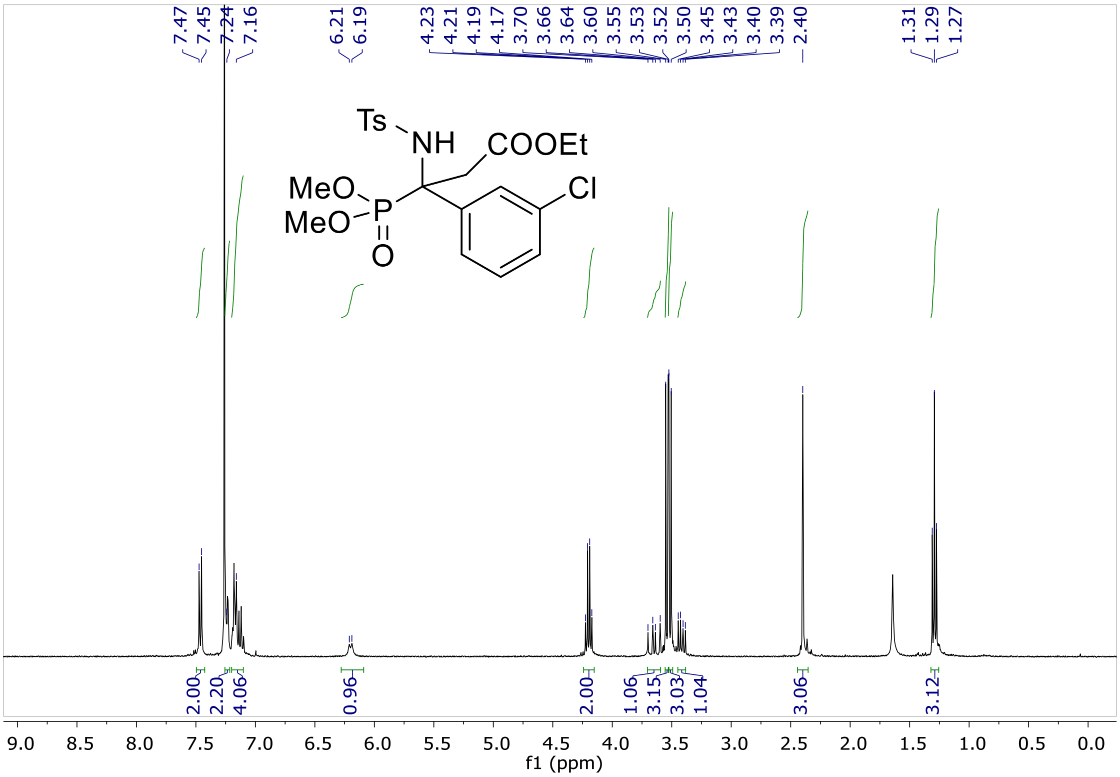

^{13}C NMR {^{1}H} (101 MHz, $CDCl_3$)

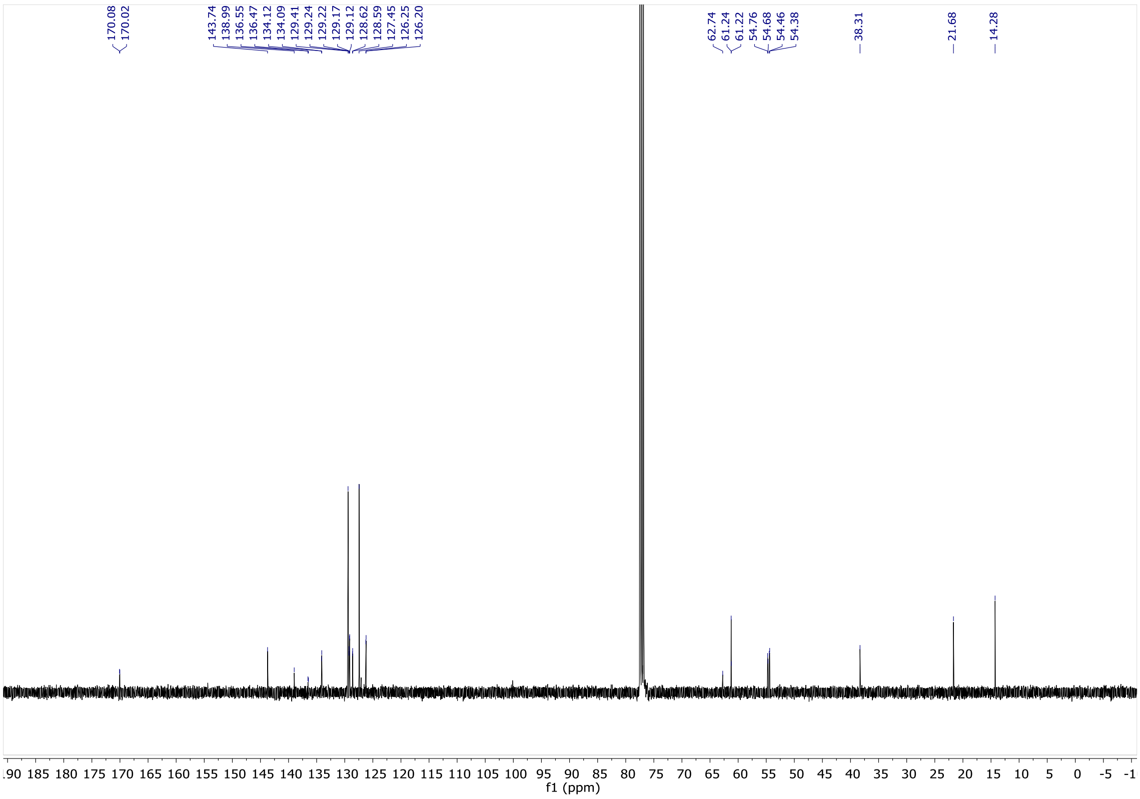

^{31}P NMR (120 MHz, $CDCl_3$)

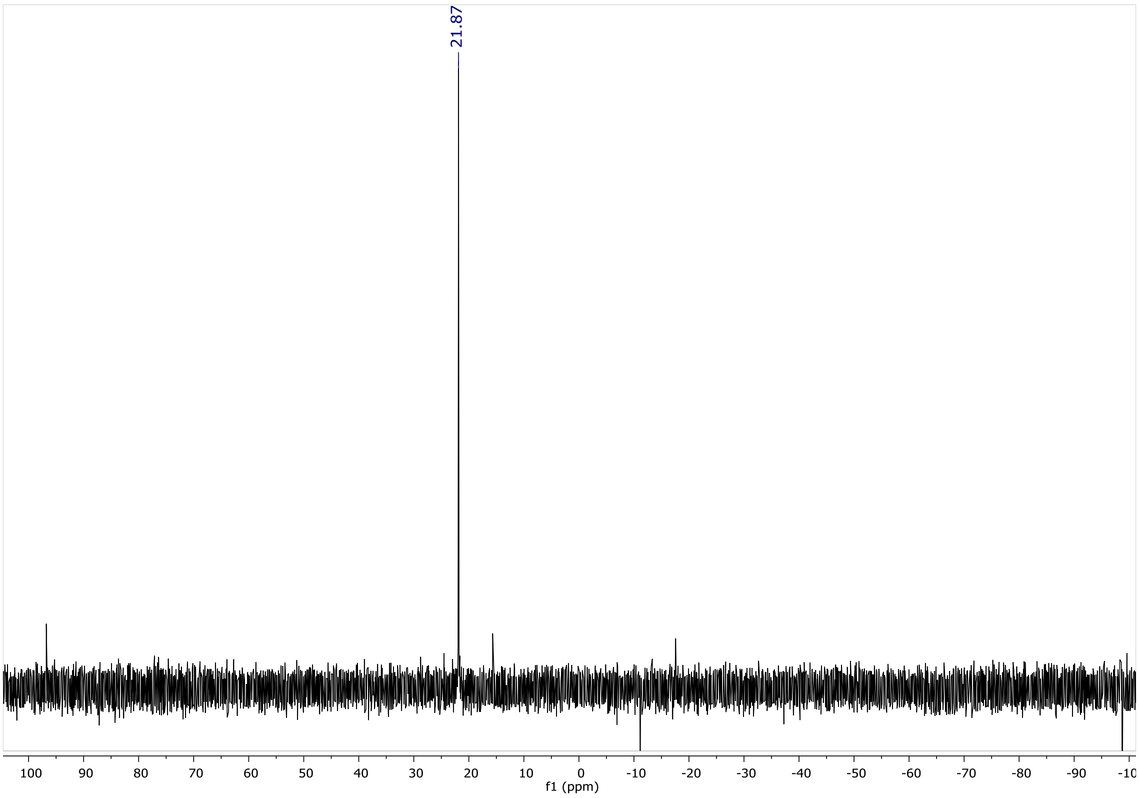

Ethyl 3-(3,4-dichlorophenyl)-3-(dimethoxyphosphoryl)-3-((4-methylphenyl)sulfonamido) propanoate (7h)

^{1}H NMR (400 MHz, $CDCl_3$)

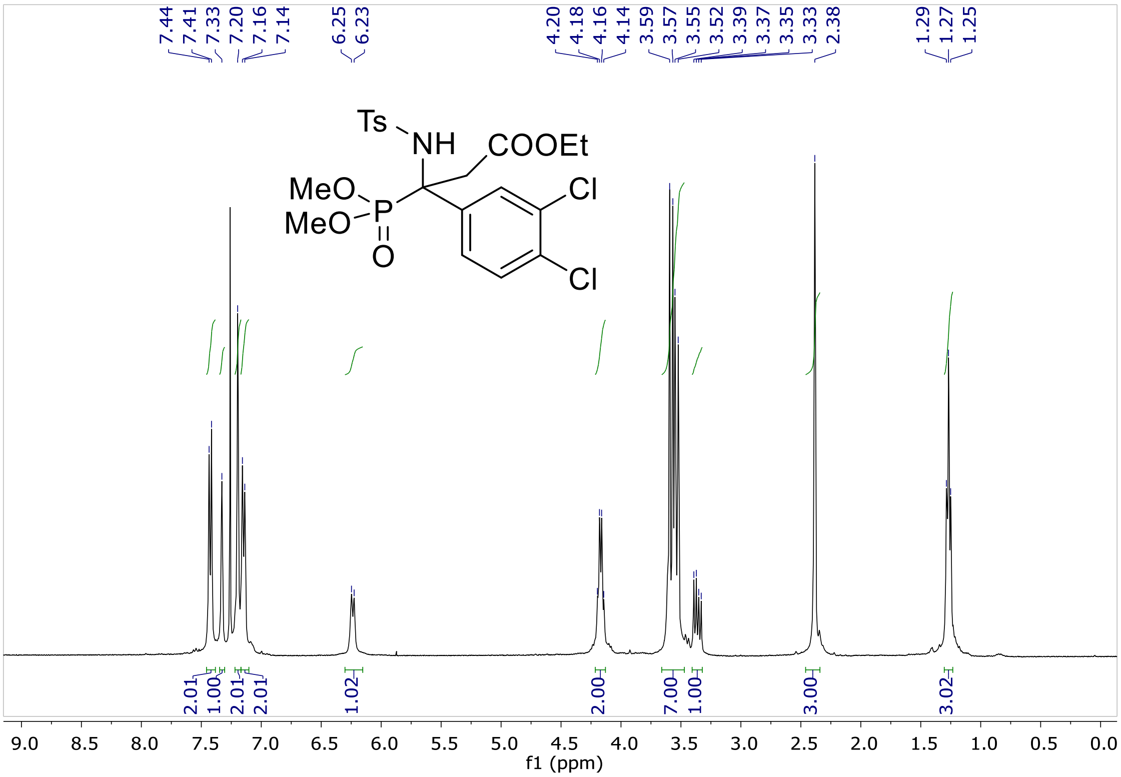

^{13}C NMR {^{1}H} (101 MHz, $CDCl_3$)

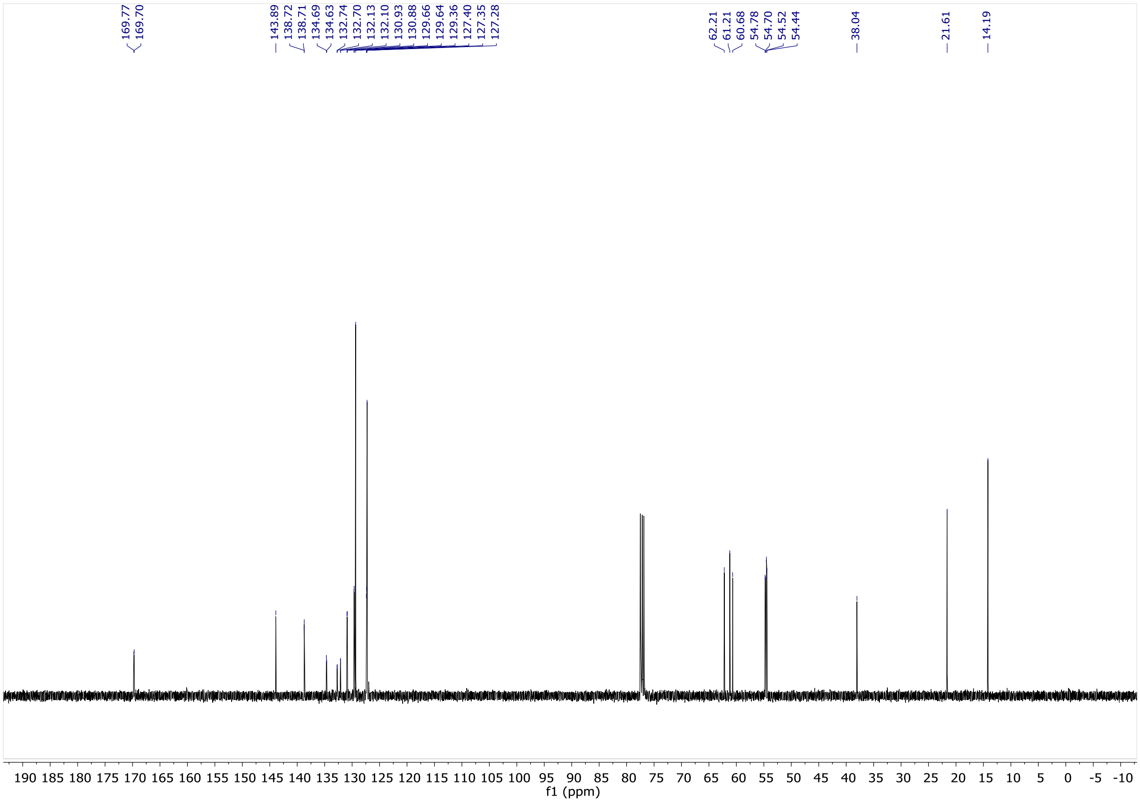

^{31}**P NMR** (120 MHz, $CDCl_3$)

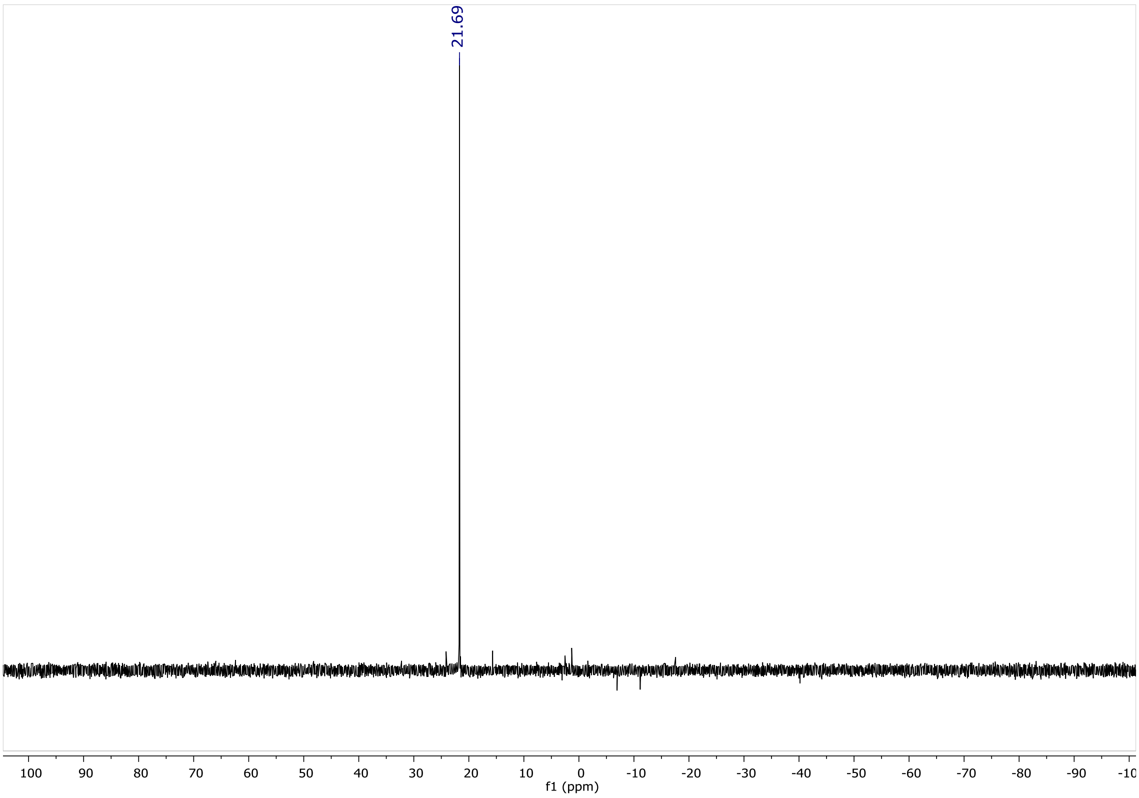

Ethyl 3-(3-chloro-4-methoxyphenyl)-3-(dimethoxyphosphoryl)-3-((4-methylphenyl) sulfonamido)propanoate (7i)

^{1}H NMR (400 MHz, $CDCl_3$)

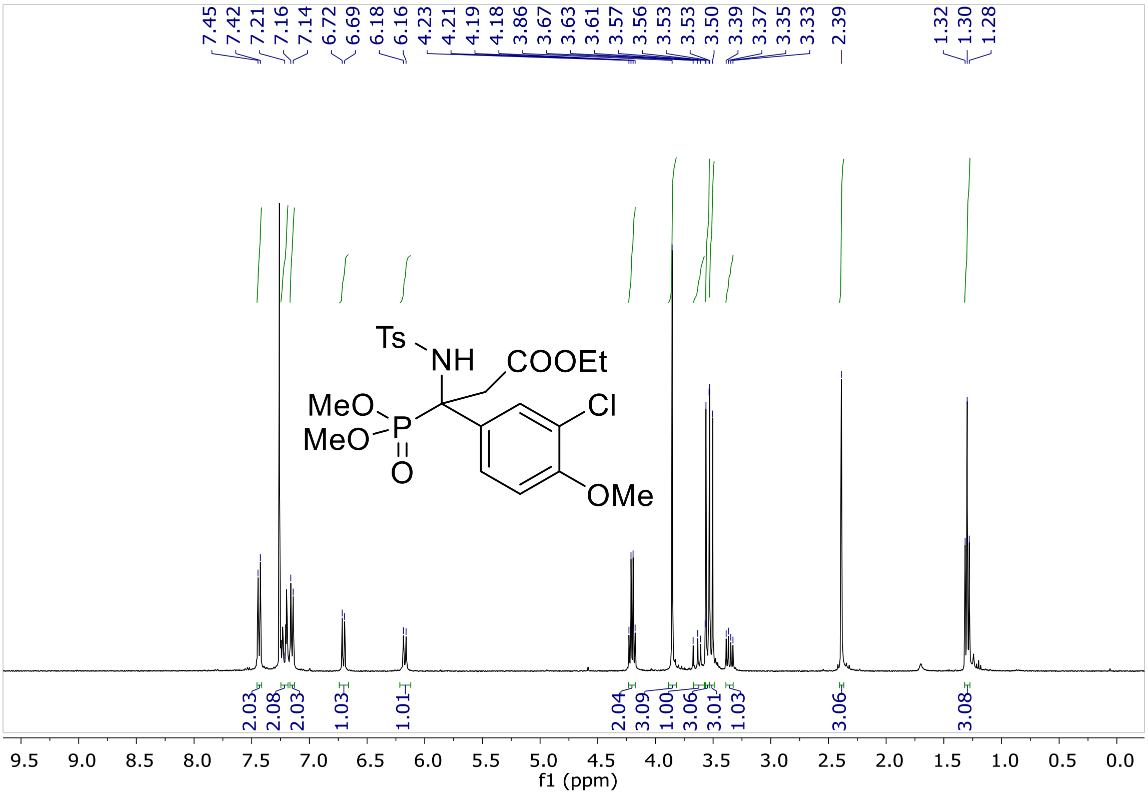

^{13}C NMR {^{1}H} (101 MHz, $CDCl_3$)

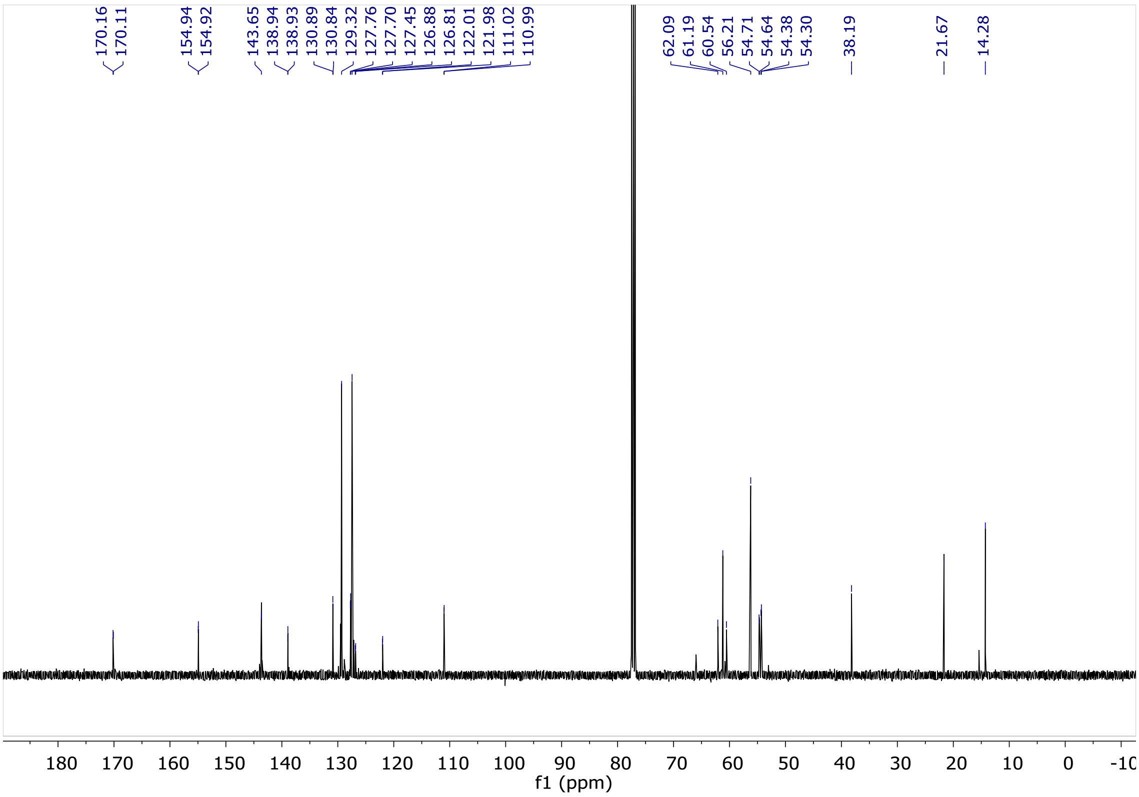

^{31}**P NMR** (120 MHz, $CDCl_3$)

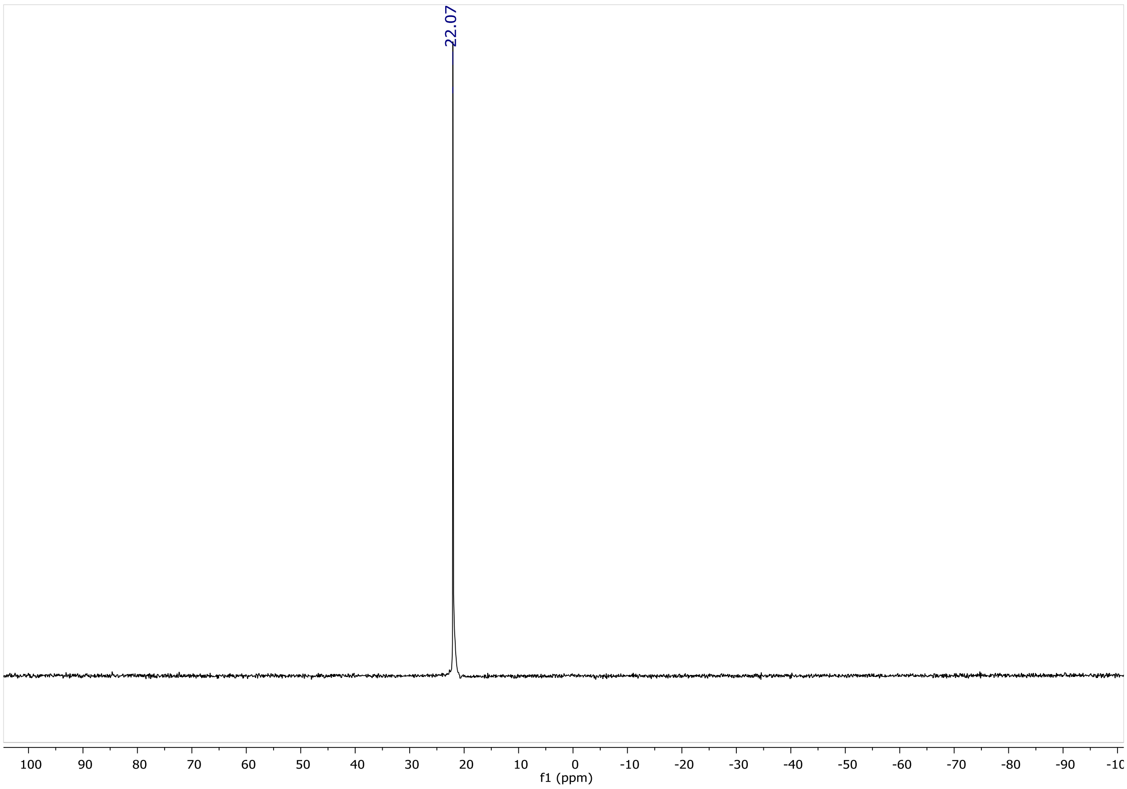

Ethyl 3-(dimethoxyphosphoryl)-3-(4-fluorophenyl)-3-((4-methylphenyl)sulfonamido) propanoate (7j)

^{1}H NMR (400 MHz, $CDCl_3$)

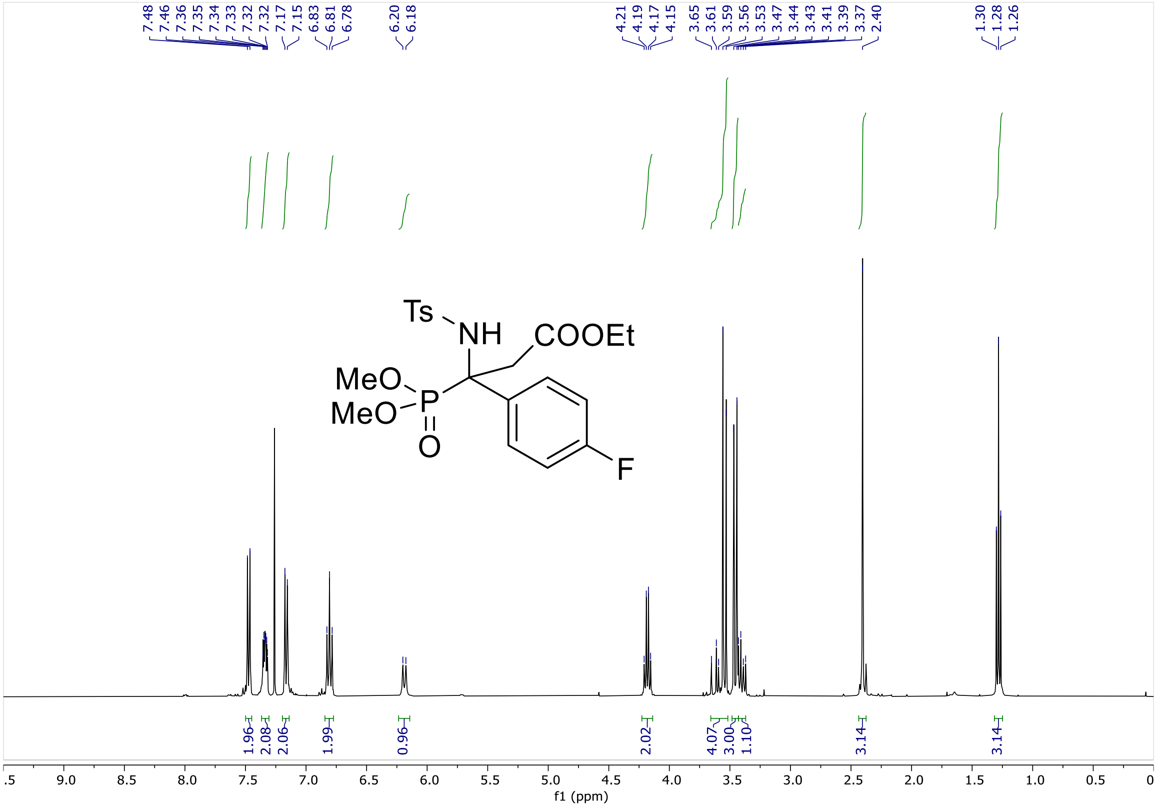

^{13}C NMR {^{1}H} (75 MHz, $CDCl_3$)

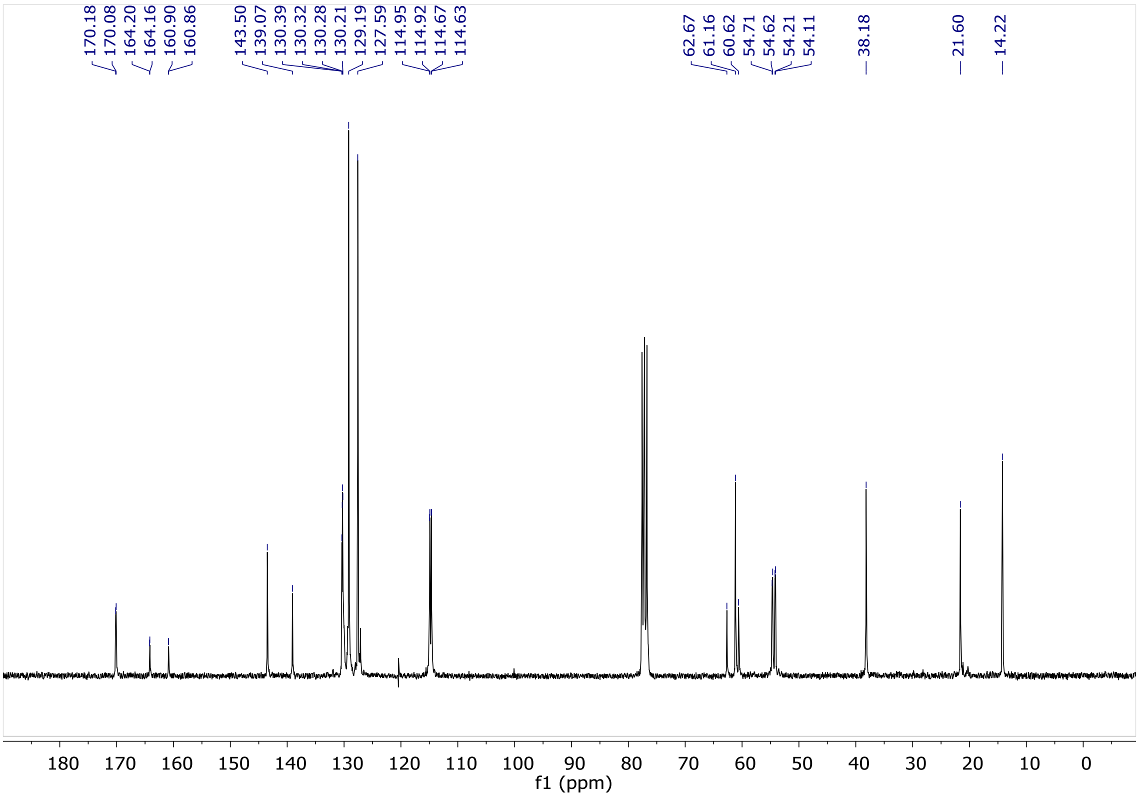

^{31}P NMR (120 MHz, $CDCl_3$)

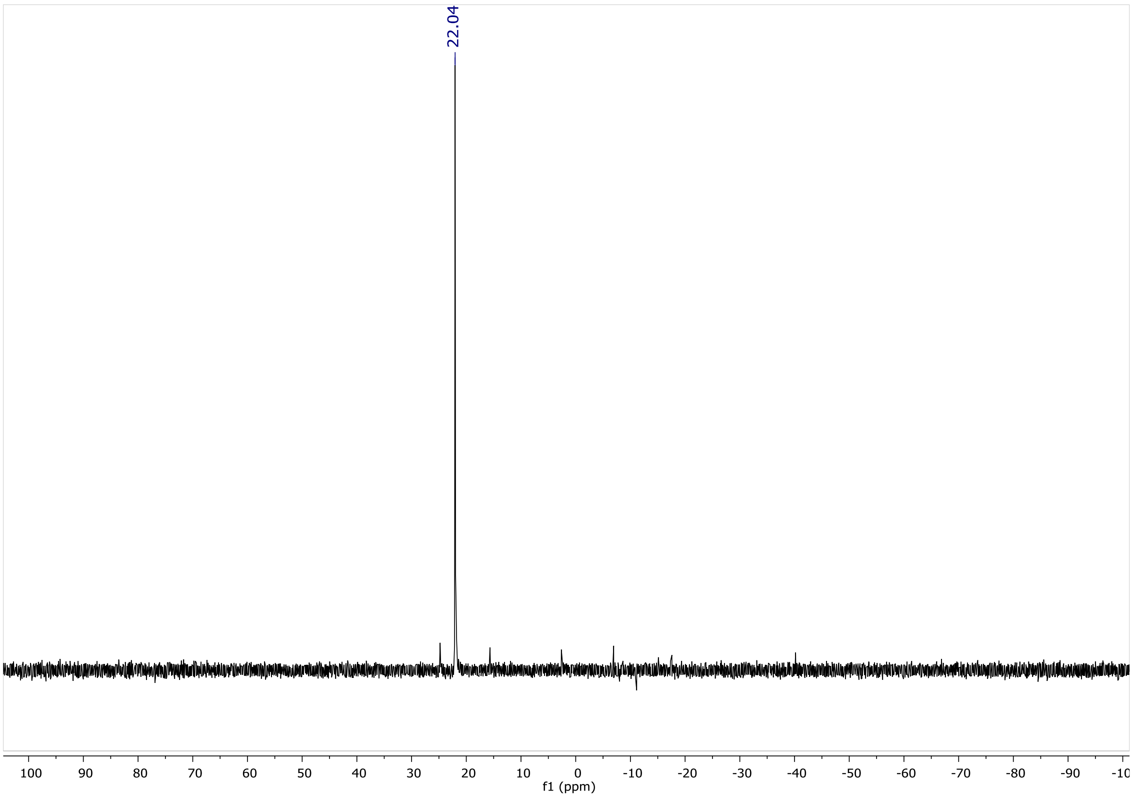

^{19}F NMR (282 MHz, $CDCl_3$)

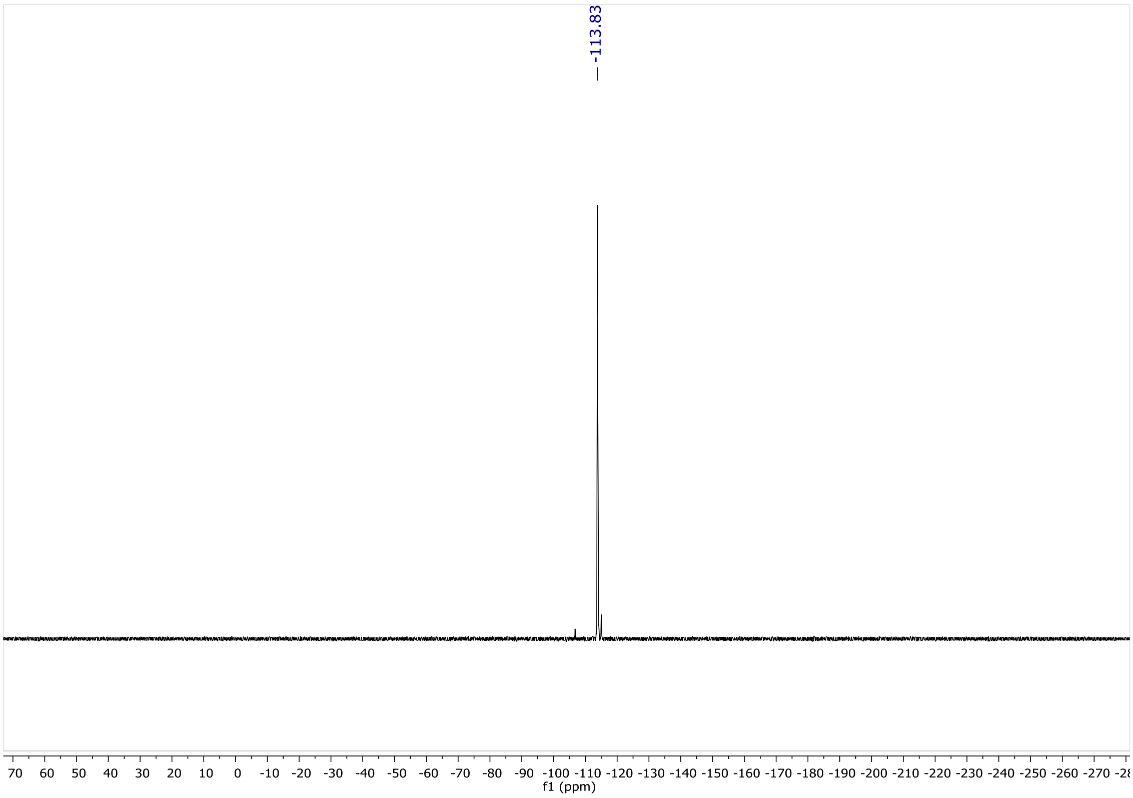

Ethyl 3-(dimethoxyphosphoryl)-3-(3-fluorophenyl)-3-((4-methylphenyl)sulfonamido) propanoate (7k)

^{1}H NMR (400 MHz, $CDCl_3$)

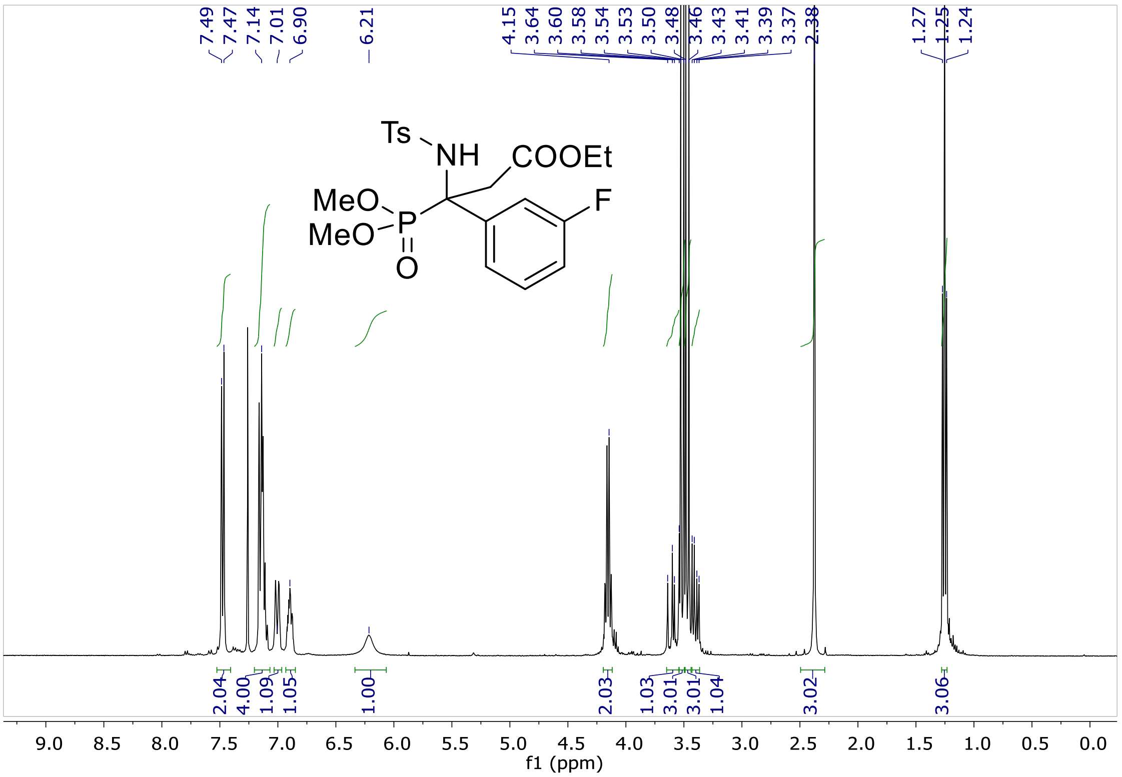

^{13}C NMR {^{1}H} (101 MHz, $CDCl_3$)

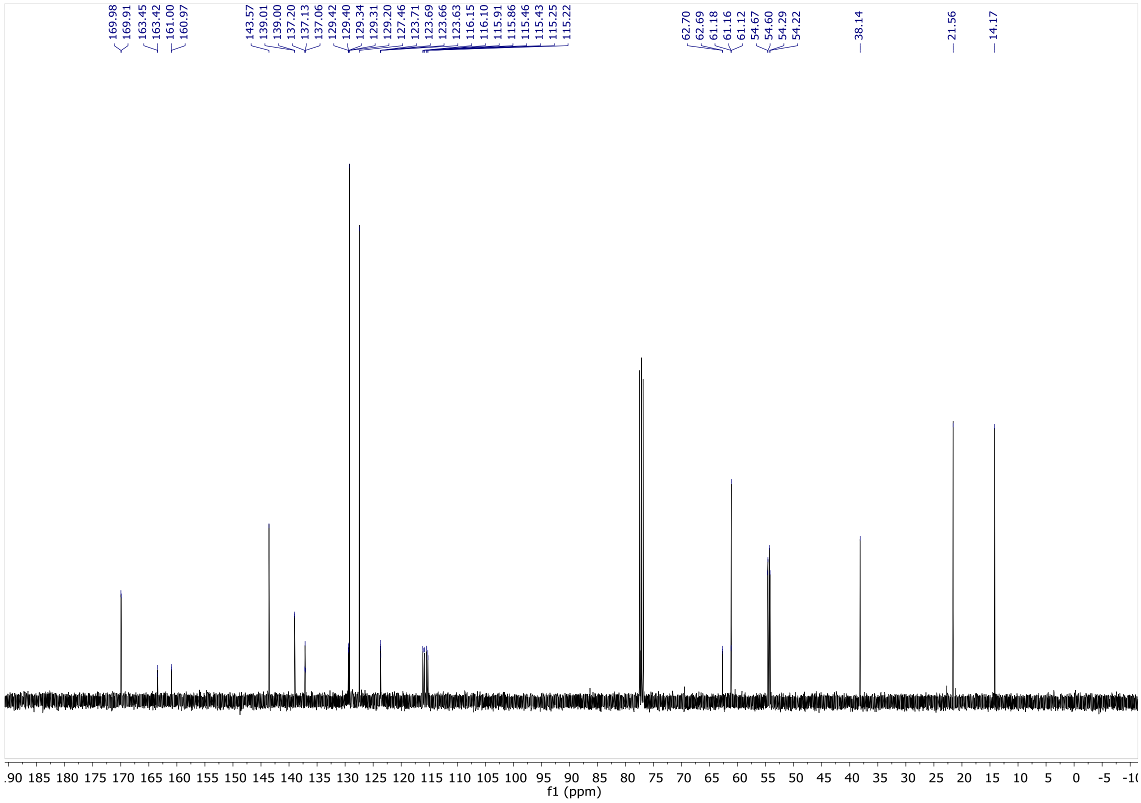

^{31}**P NMR** (120 MHz, $CDCl_3$)

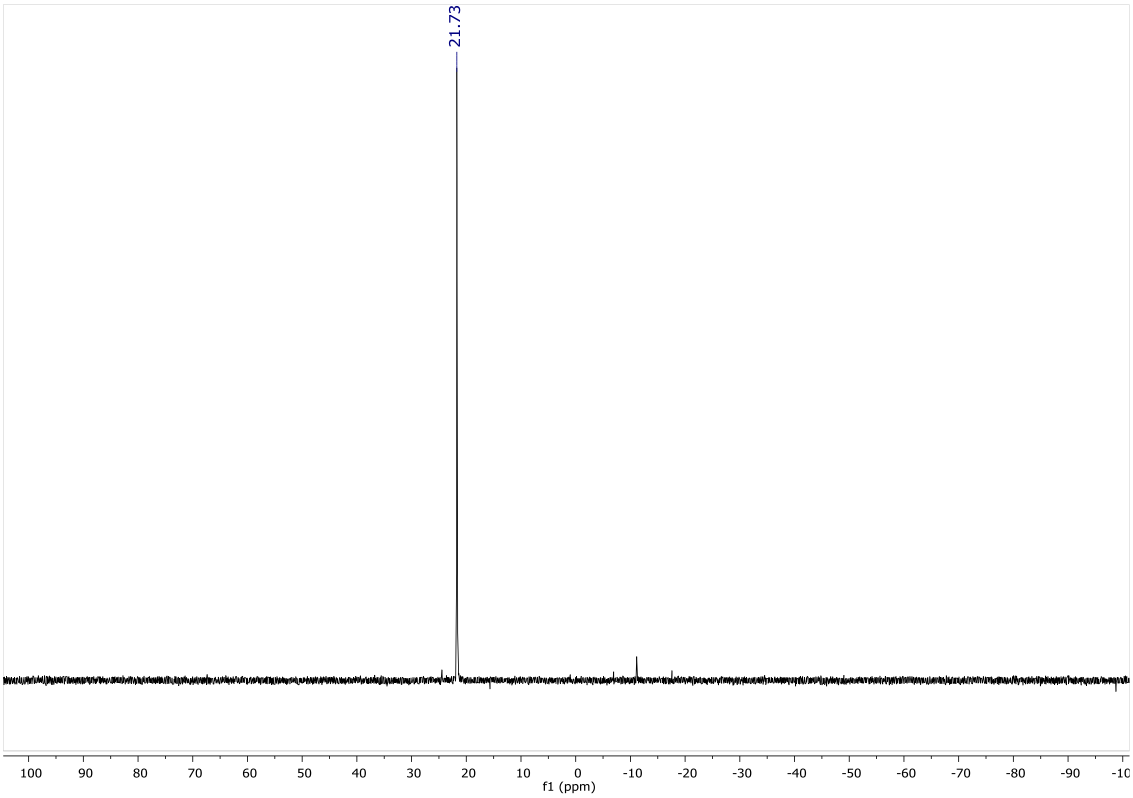

^{19}**F NMR** (282 MHz, $CDCl_3$)

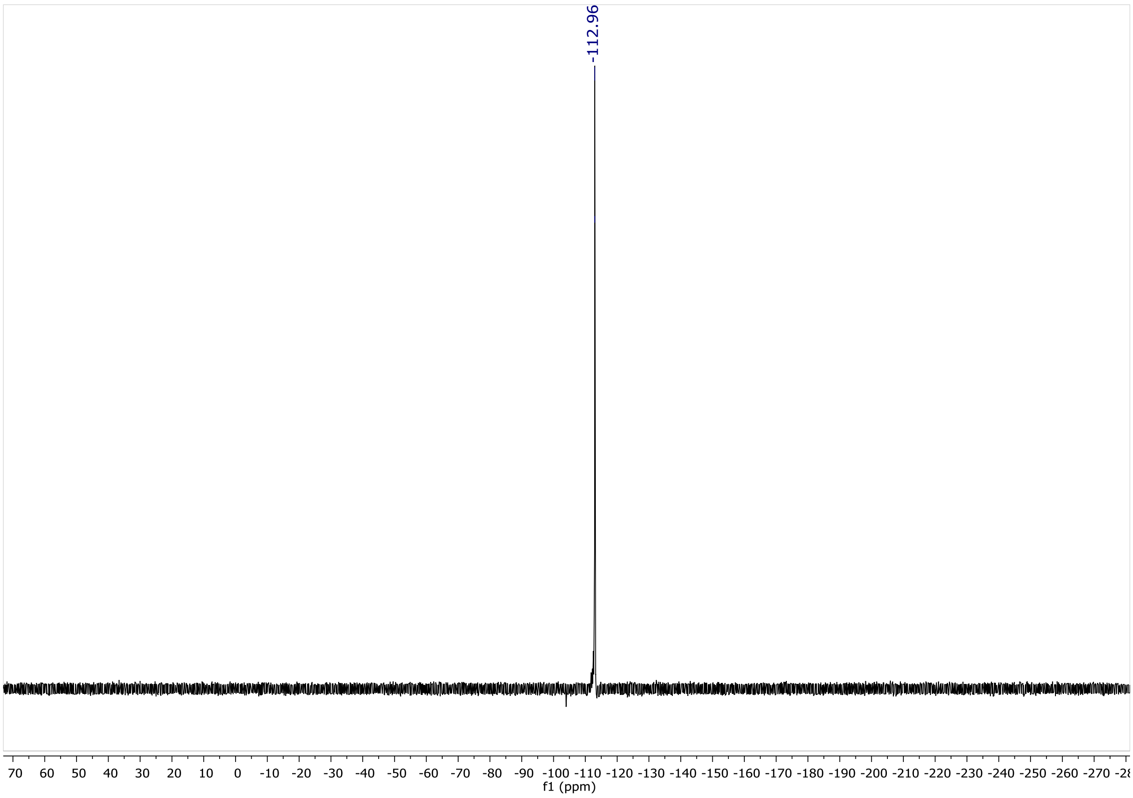

Ethyl 3-(dimethoxyphosphoryl)-3-(2-fluorophenyl)-3-((4-methylphenyl)sulfonamido) propanoate (7l)

^{1}H NMR (400 MHz, $CDCl_3$)

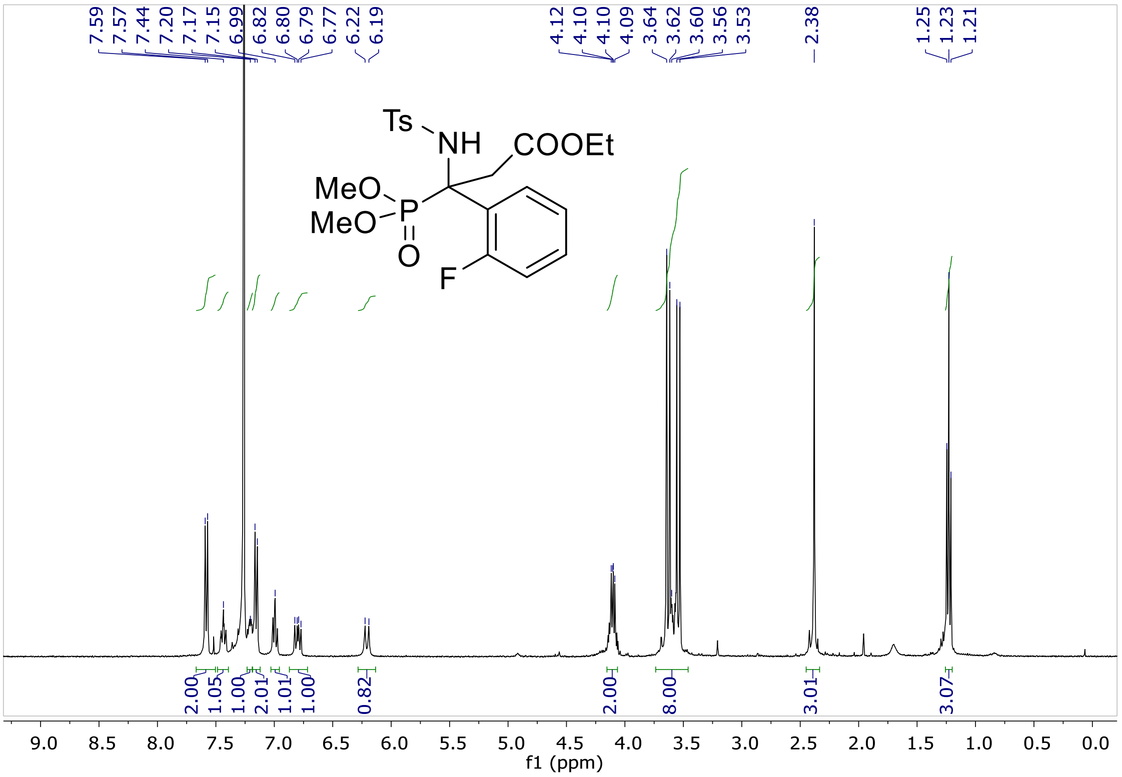

^{13}C NMR {^{1}H} (101 MHz, $CDCl_3$)

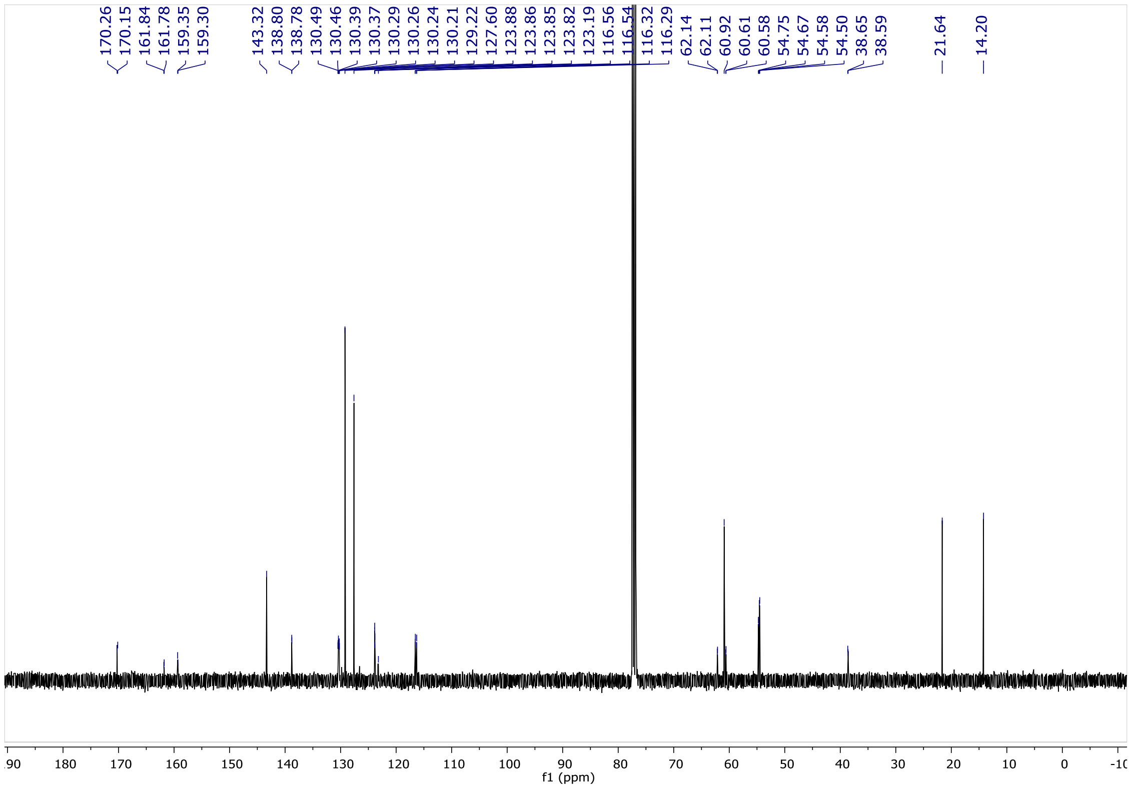

^{31}P NMR (120MHz, $CDCl_3$)

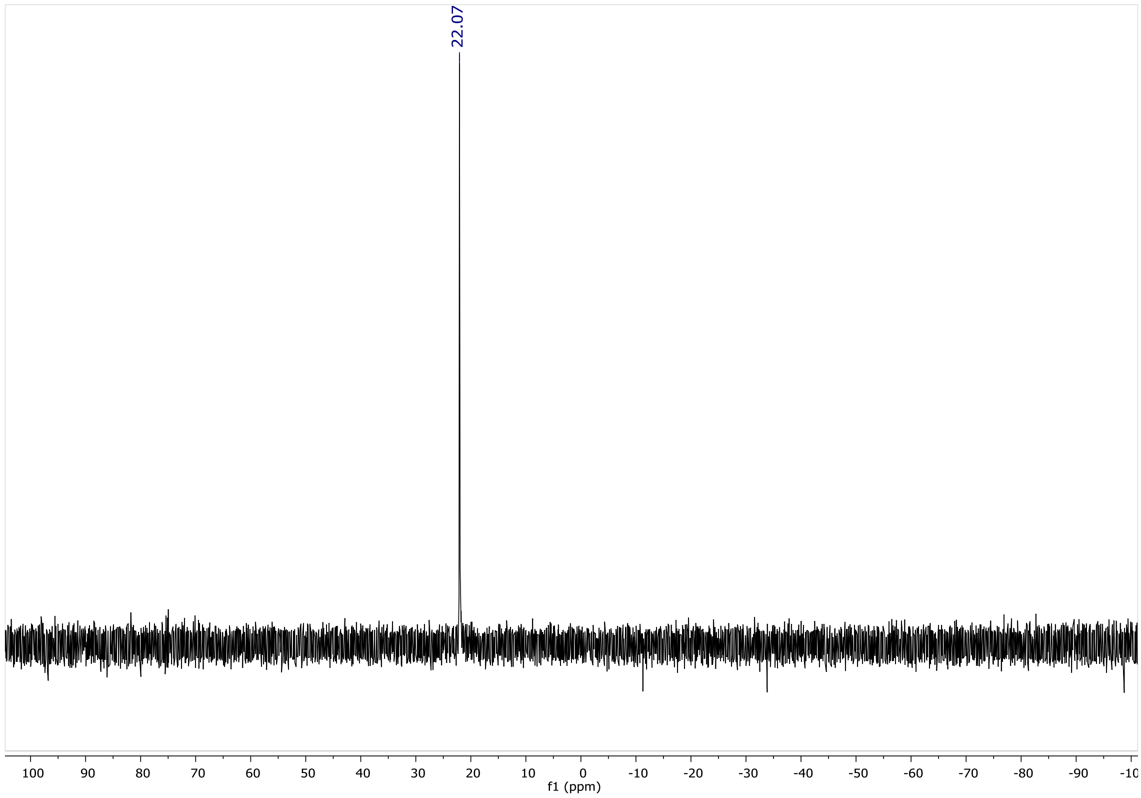

^{19}F NMR (282 MHz, $CDCl_3$)

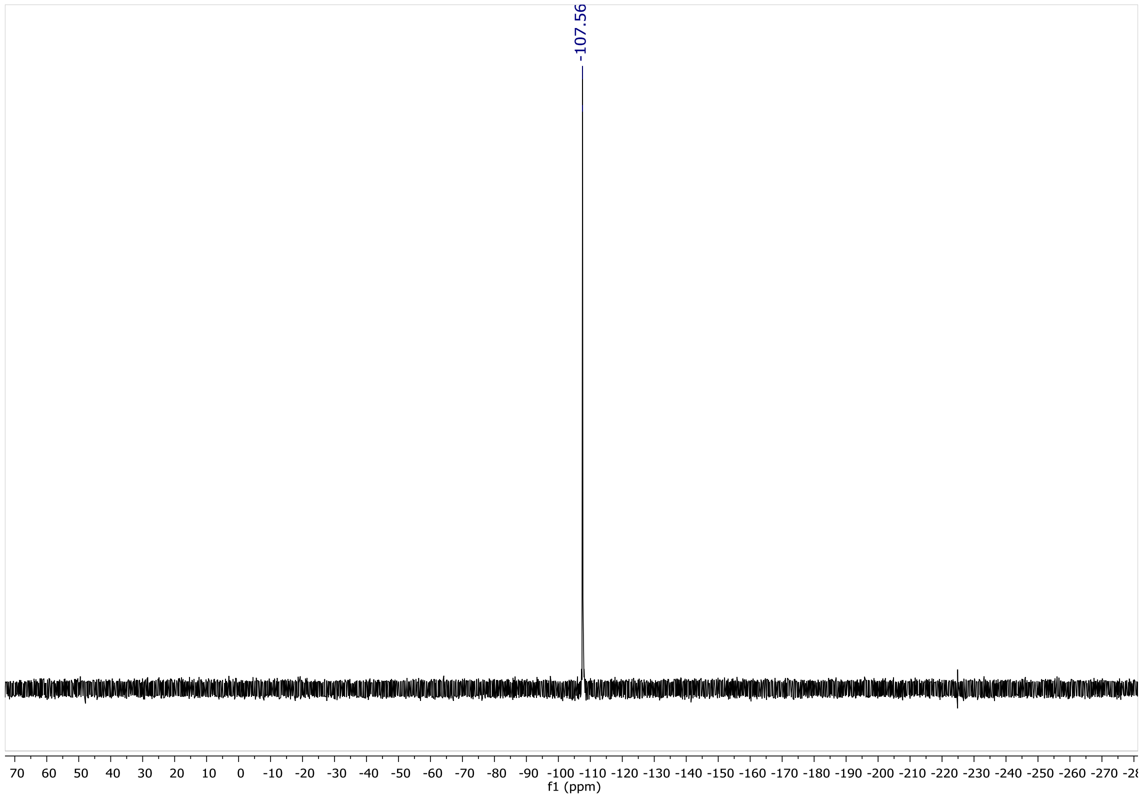

Ethyl 3-(2,4-difluorophenyl)-3-(dimethoxyphosphoryl)-3-((4-methylphenyl)sulfonamido) propanoate (7m)

^{1}H NMR (400 MHz, $CDCl_3$)

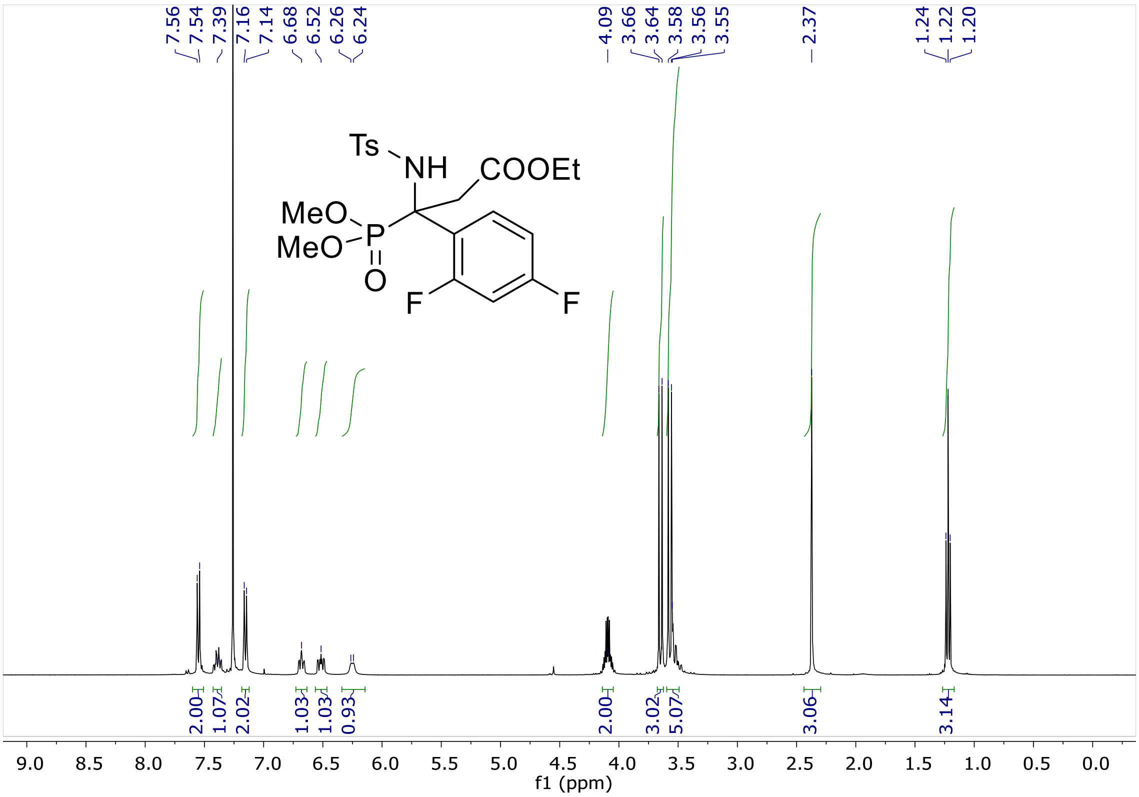

^{13}C NMR {^{1}H} (101 MHz, $CDCl_3$)

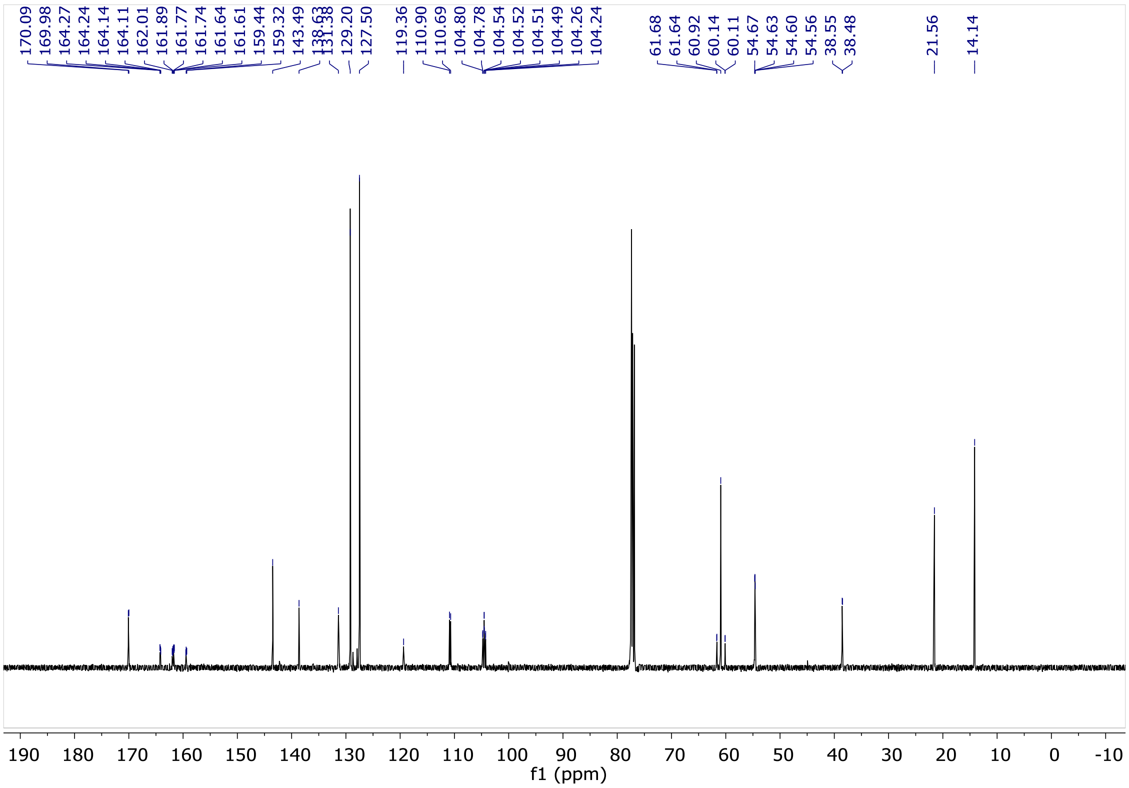

^{31}P NMR (120 MHz, $CDCl_3$)

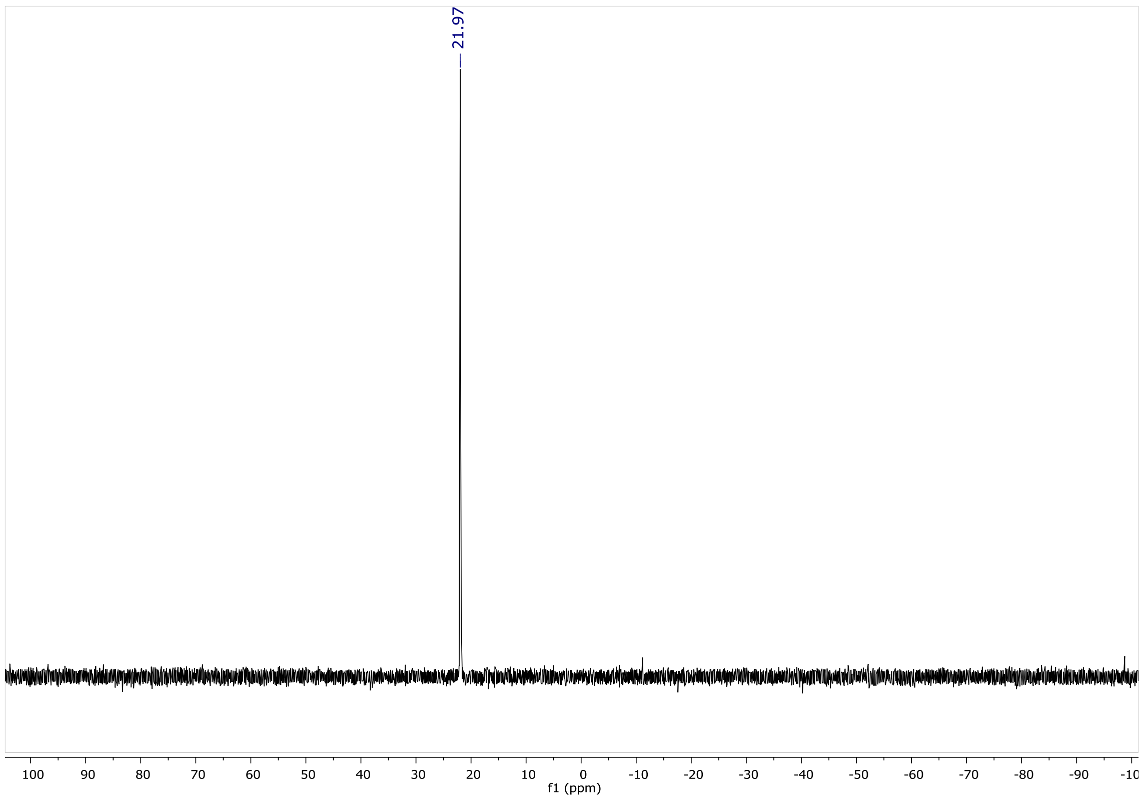

^{19}F NMR (282 MHz, $CDCl_3$)

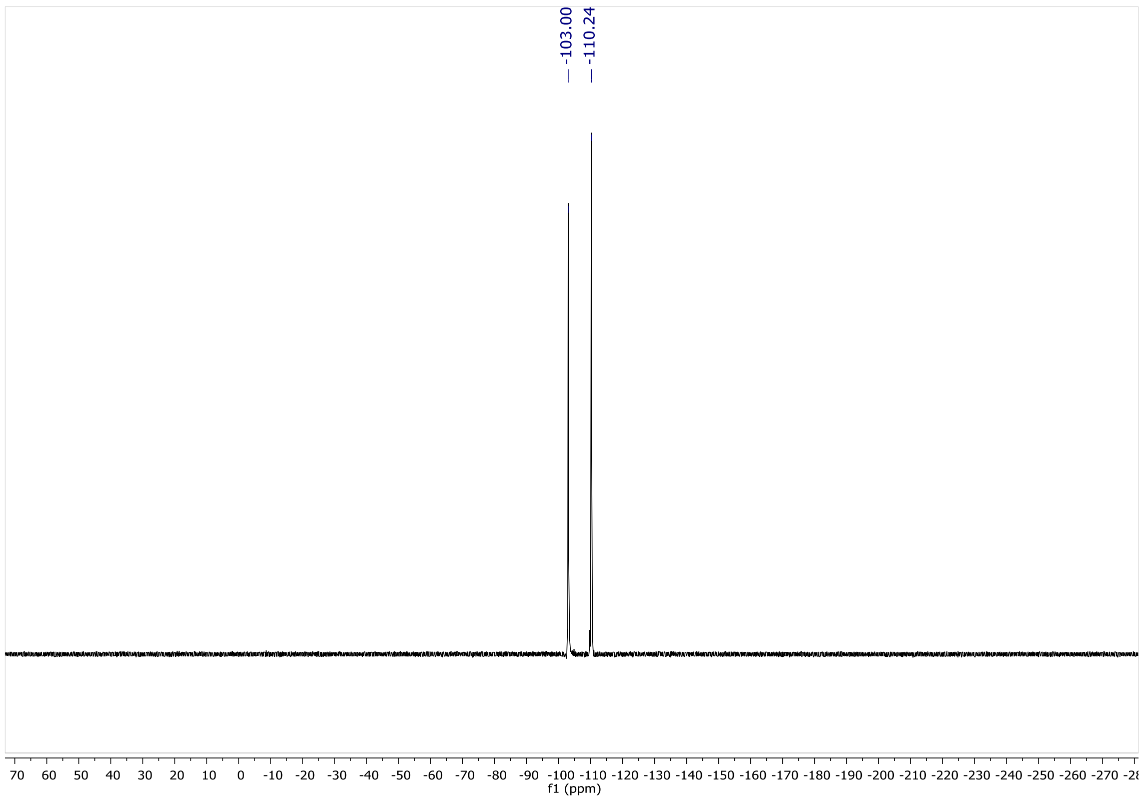

Ethyl 3-(3,4-difluorophenyl)-3-(dimethoxyphosphoryl)-3-((4-methylphenyl)sulfonamido) propanoate (7n)

^{1}H NMR (400 MHz, $CDCl_3$)

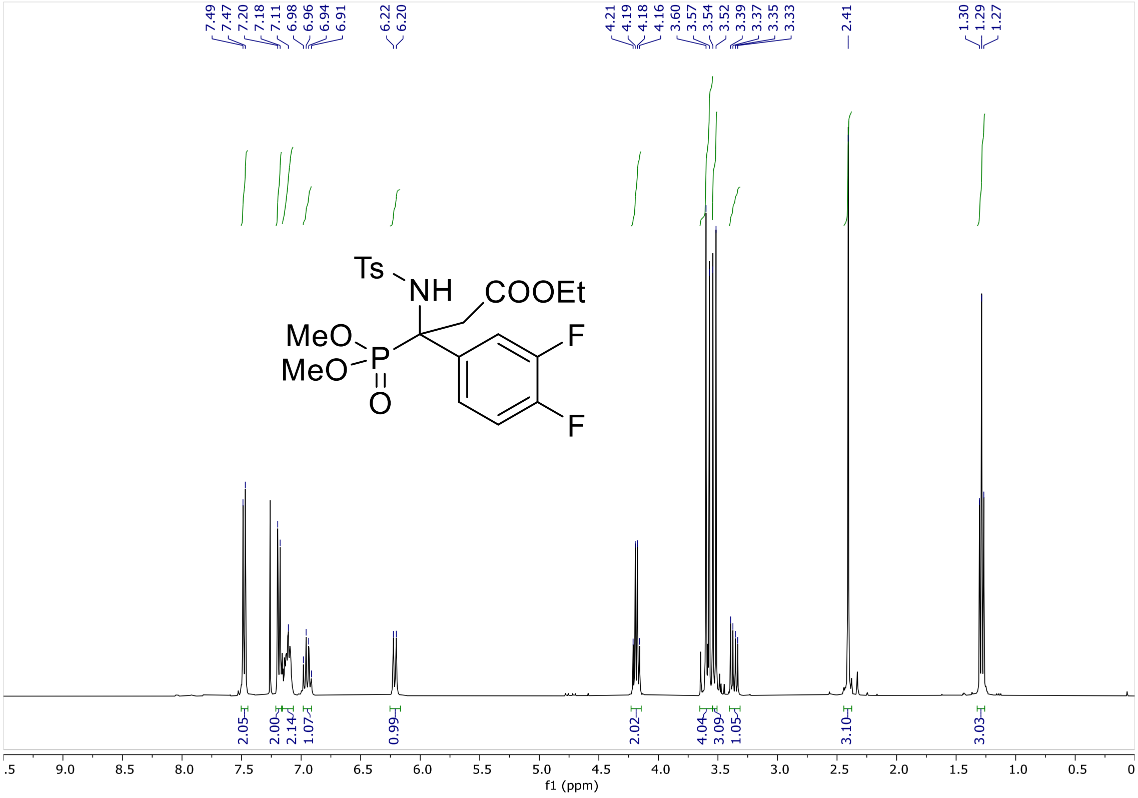

^{13}C NMR {^{1}H} (101 MHz, $CDCl_3$)

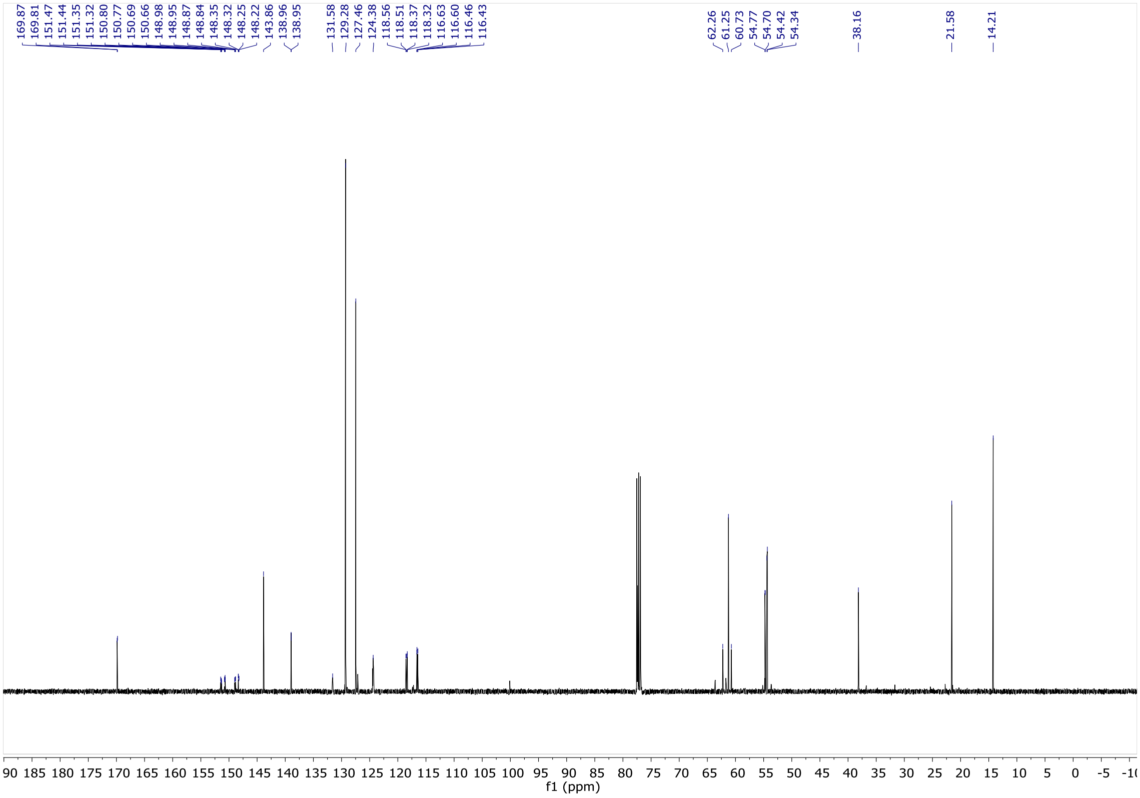

^{31}P NMR (120 MHz, $CDCl_3$)

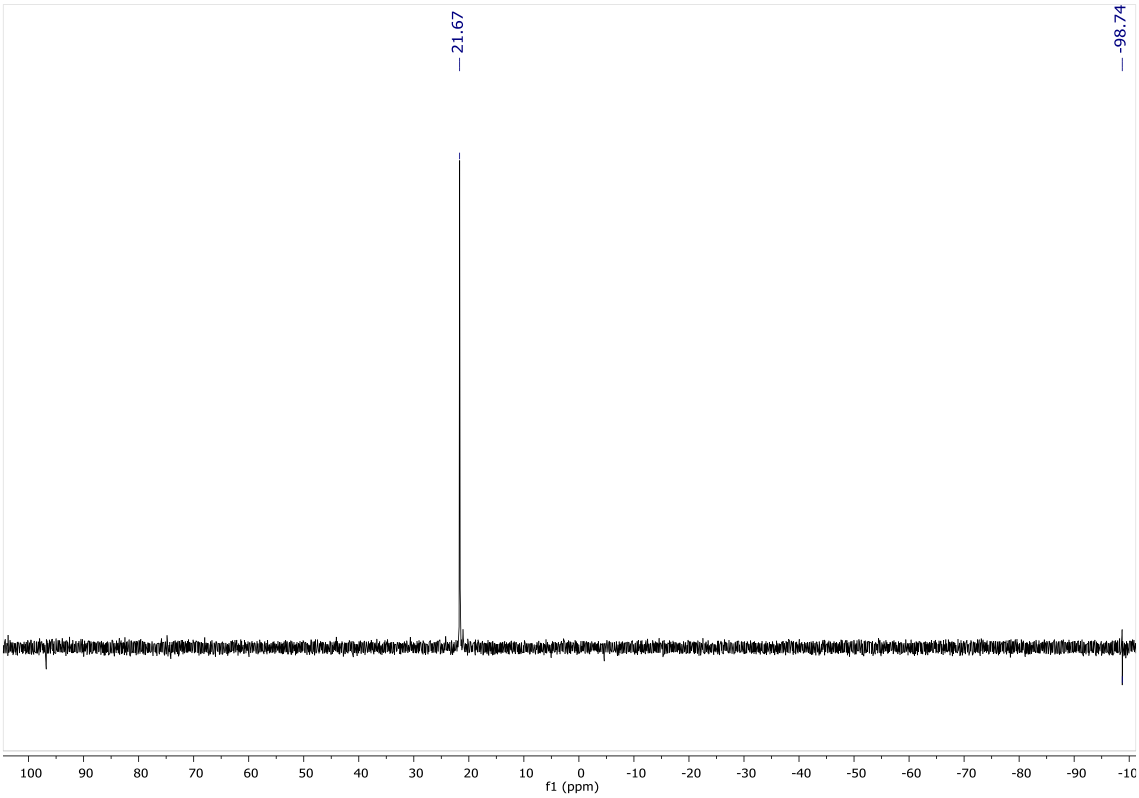

^{19}F NMR (282 MHz, $CDCl_3$)

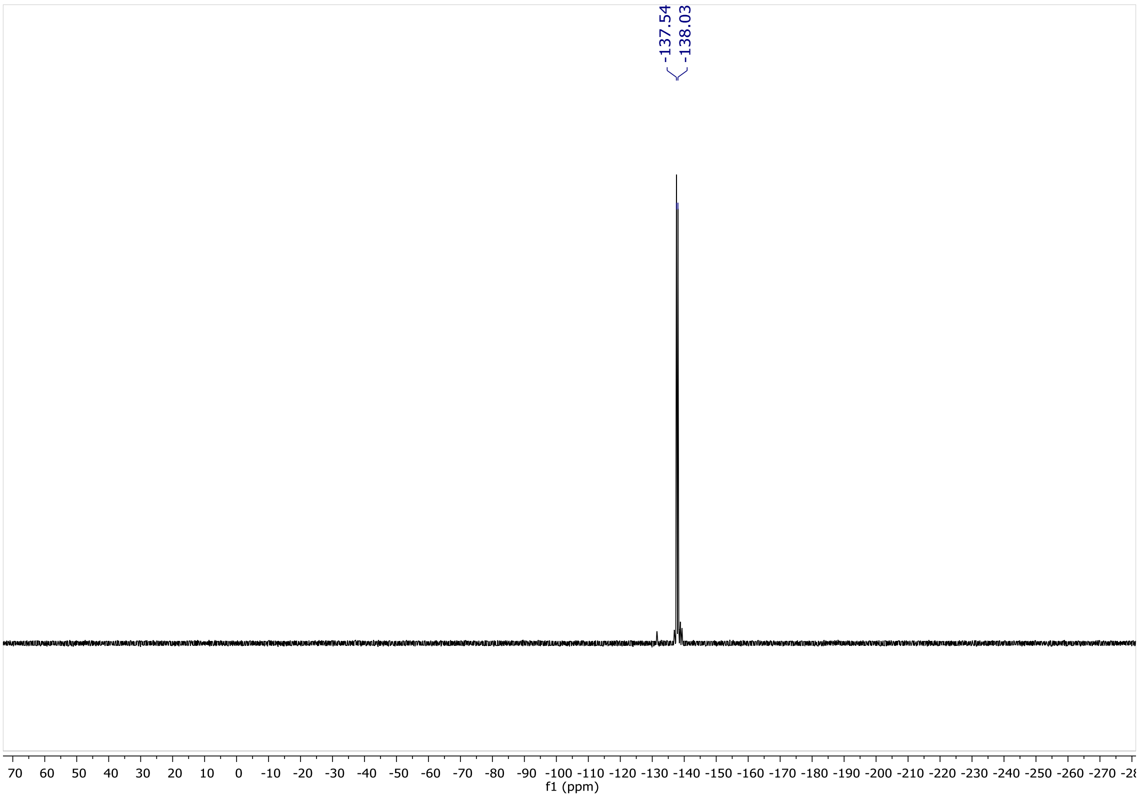

Ethyl 3-(dimethoxyphosphoryl)-3-((4-methylphenyl)sulfonamido)-3-(perfluorophenyl) propanoate (7o)

^{1}H NMR (400 MHz, $CDCl_3$)

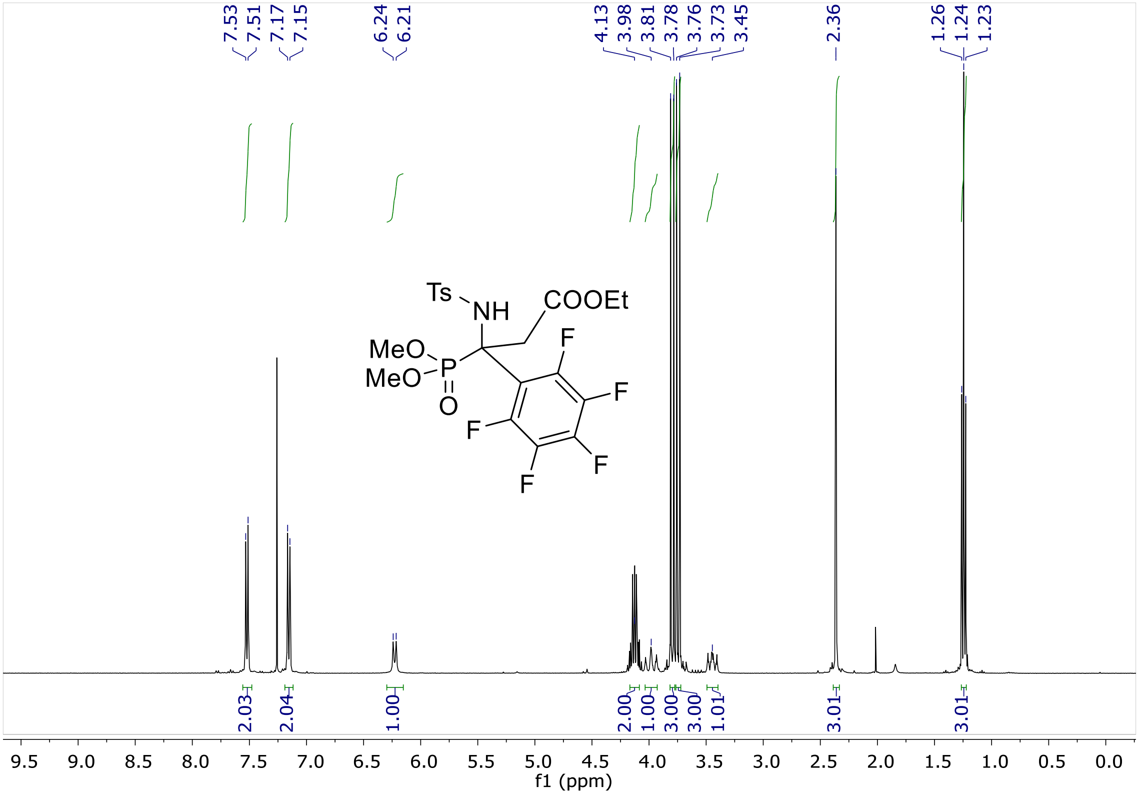

^{13}C NMR {^{1}H} (101 MHz, $CDCl_3$)

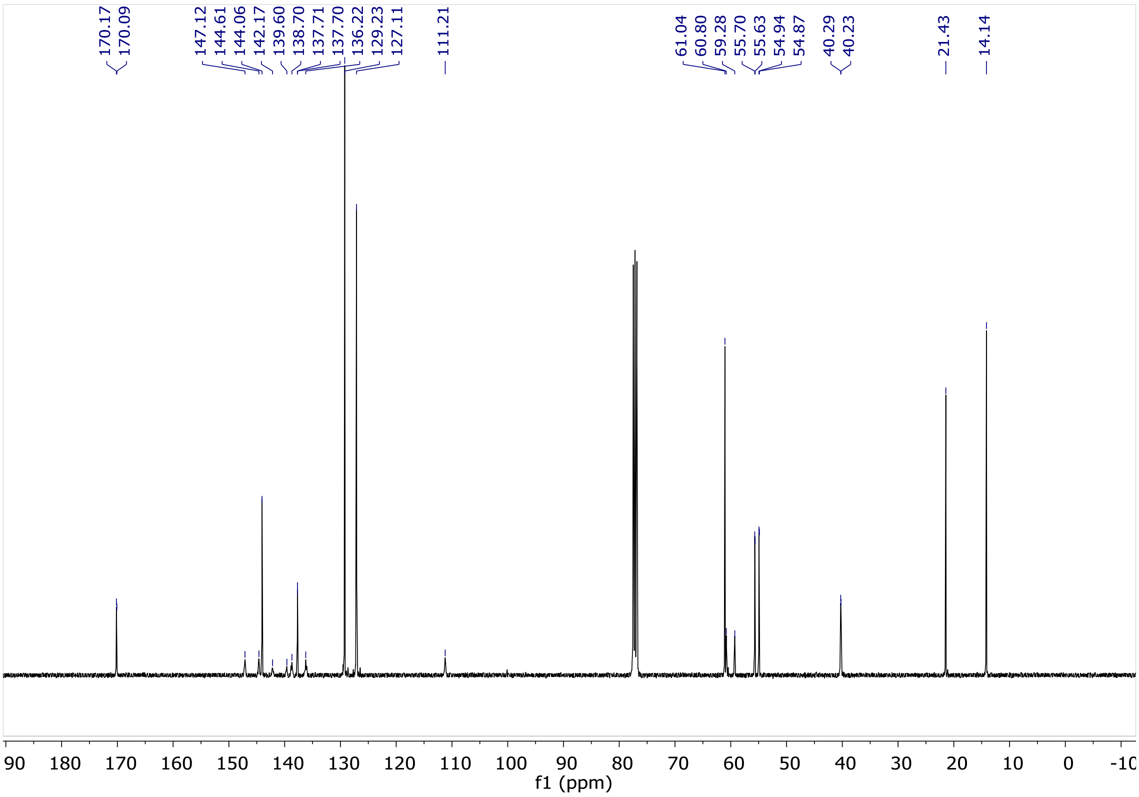

^{31}**P NMR** (120 MHz, $CDCl_3$)

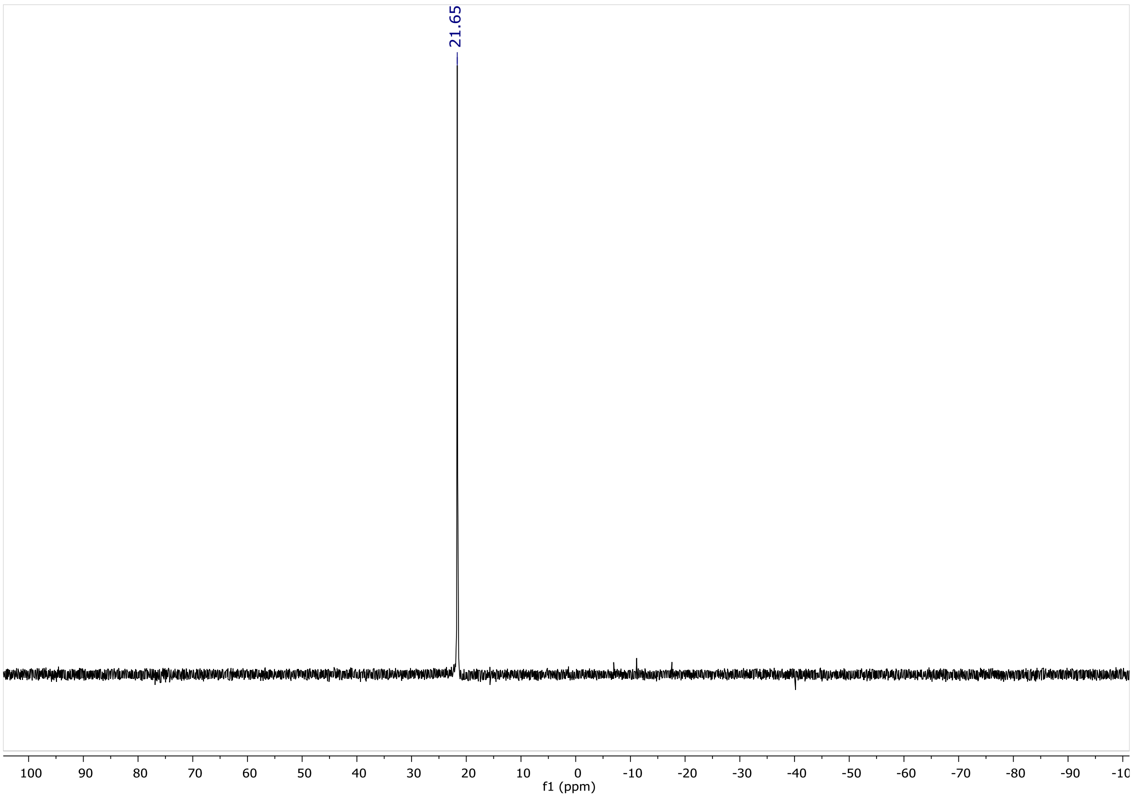

^{19}**F NMR** (282 MHz, $CDCl_3$)

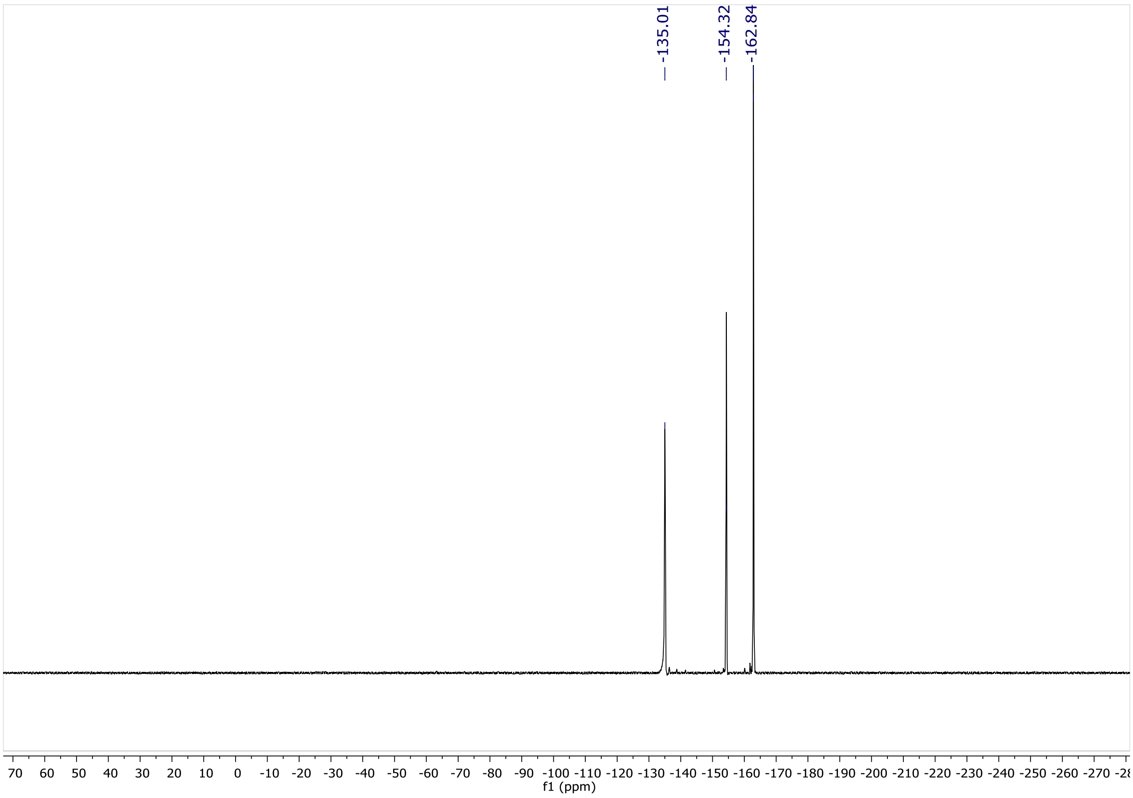

Ethyl 3-(dimethoxyphosphoryl)-3-((4-methylphenyl)sulfonamido)-3-(4-(trifluoromethyl)phenyl) propanoate (7p)

^{1}H NMR (400 MHz, $CDCl_3$)

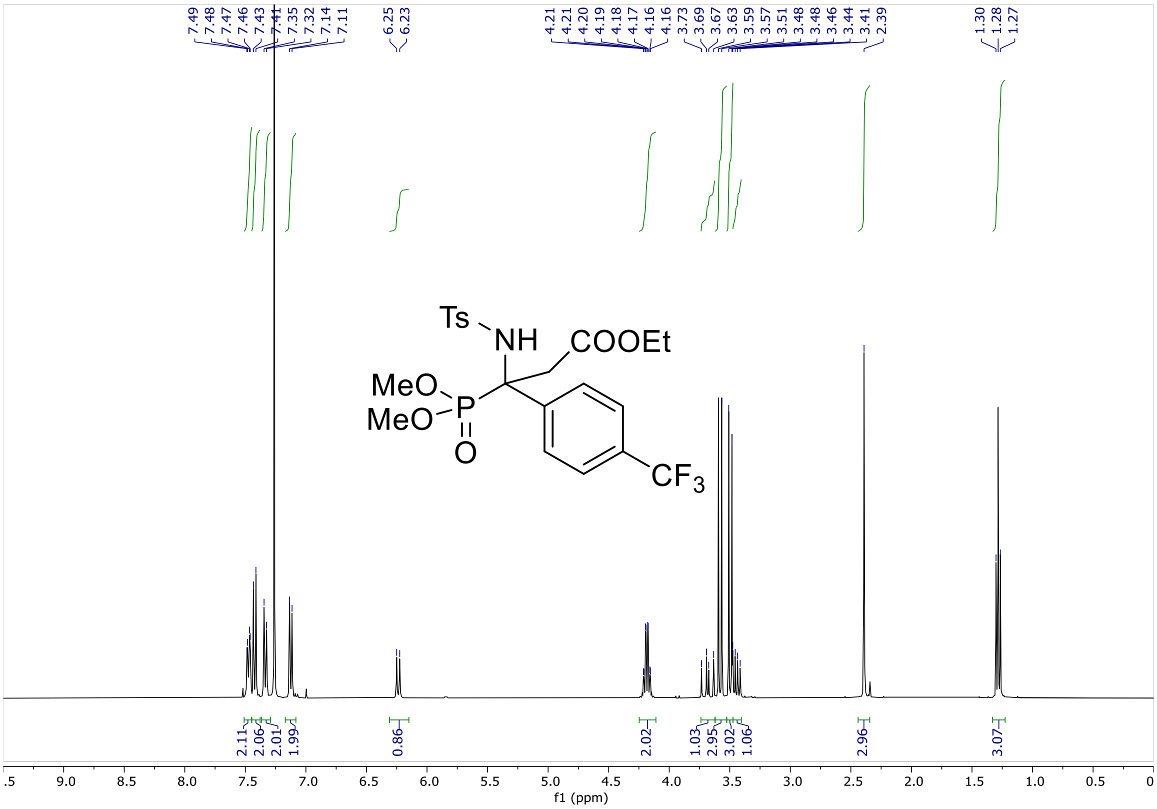

^{13}C NMR {^{1}H} (75 MHz, $CDCl_3$)

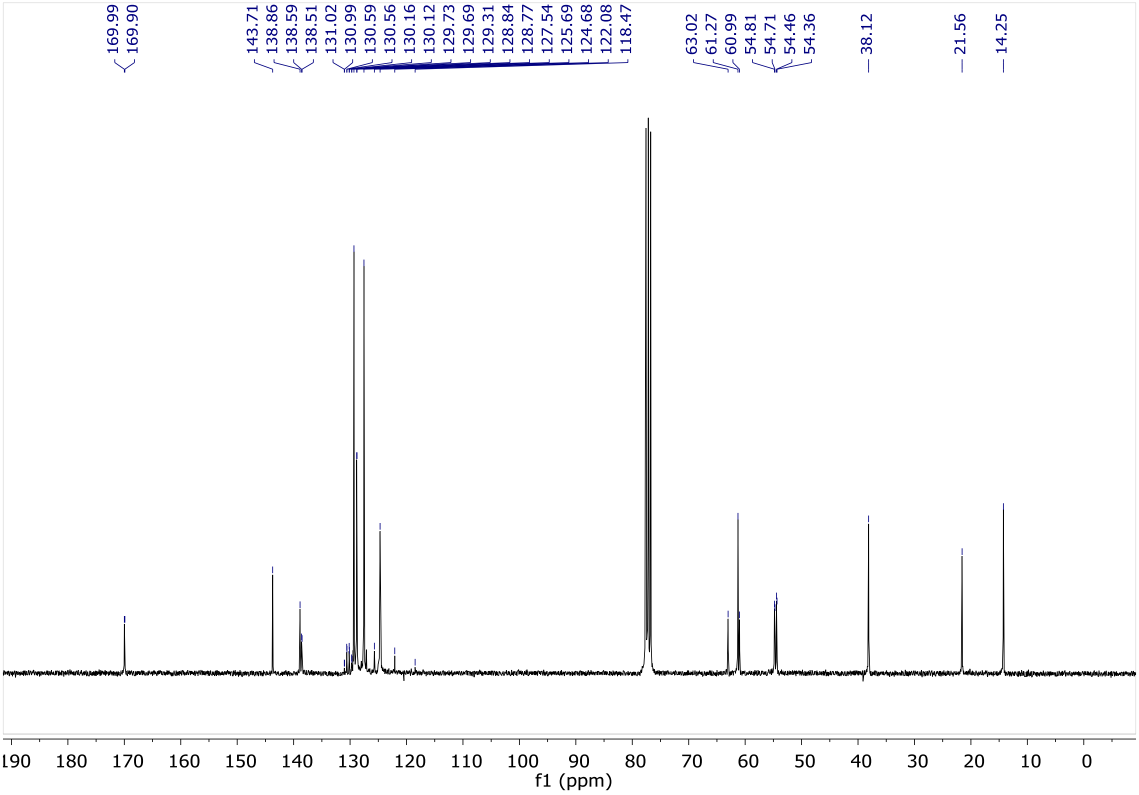

^{31}**P NMR** (120 MHz, $CDCl_3$)

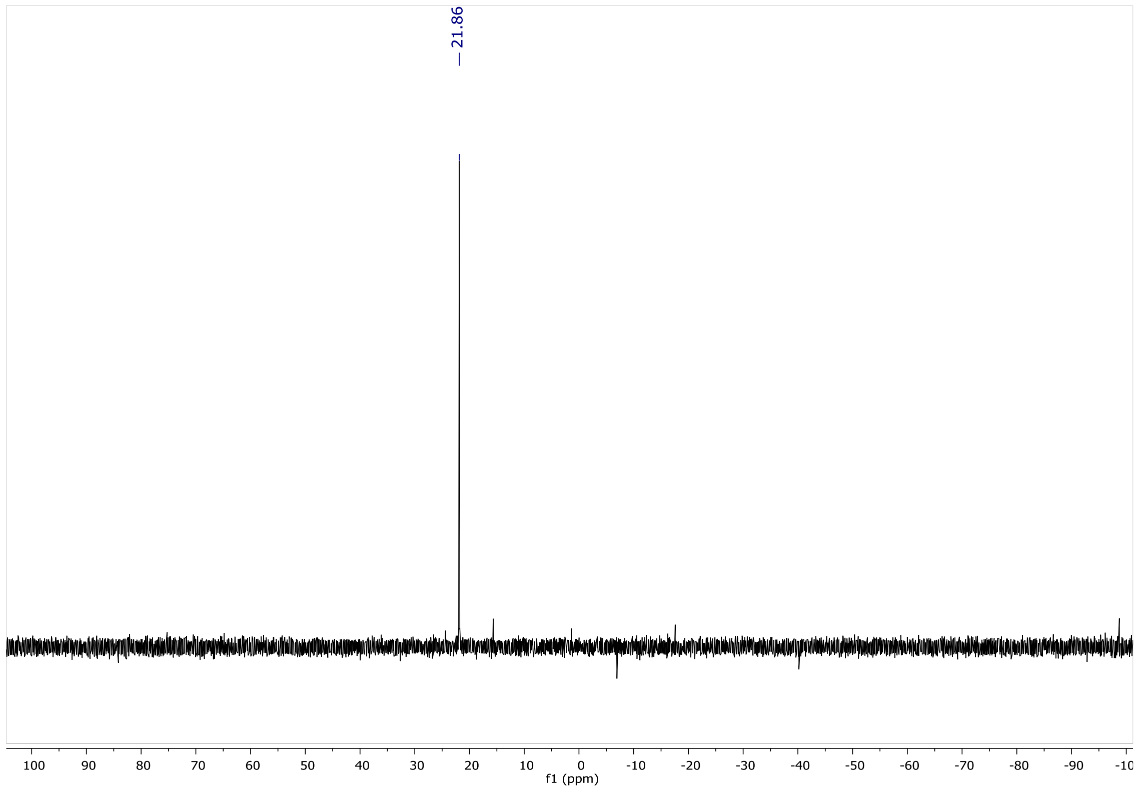

^{19}**F NMR** (282 MHz, $CDCl_3$)

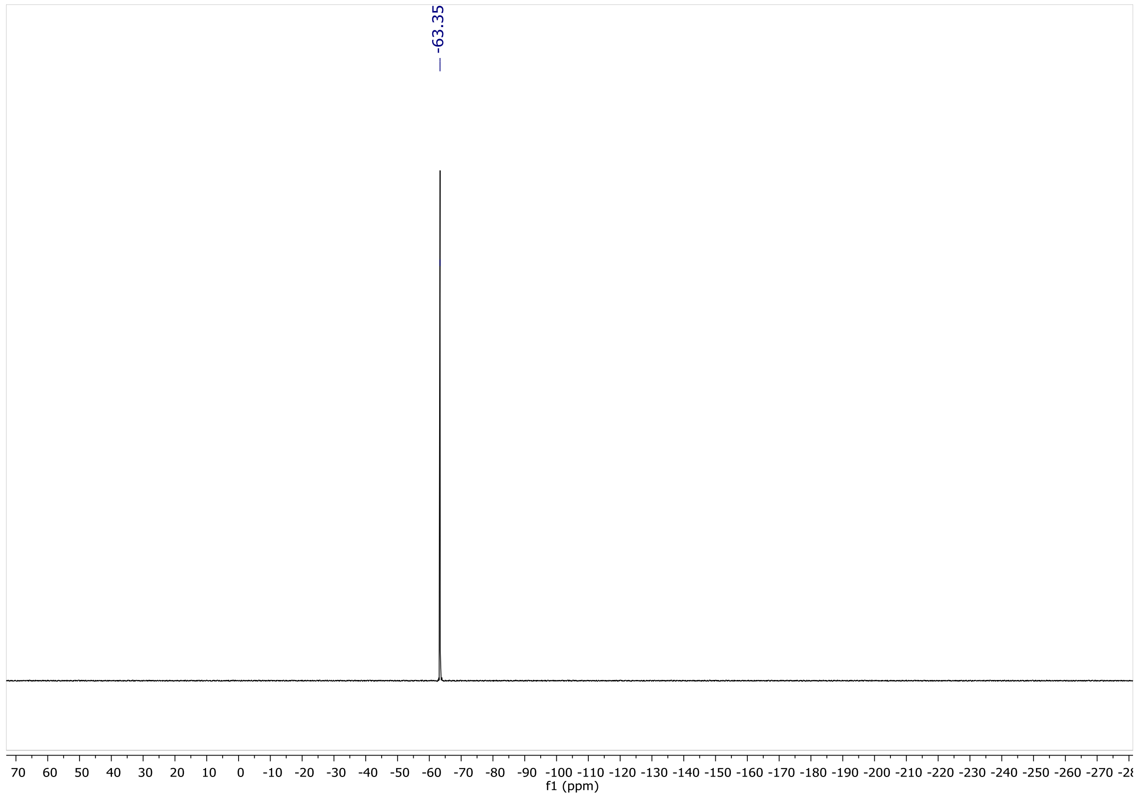

Ethyl 3-(dimethoxyphosphoryl)-3-((4-methylphenyl)sulfonamido)-3-(4-nitrophenyl)propanoate (7q)

^{1}H NMR (400 MHz, $CDCl_3$)

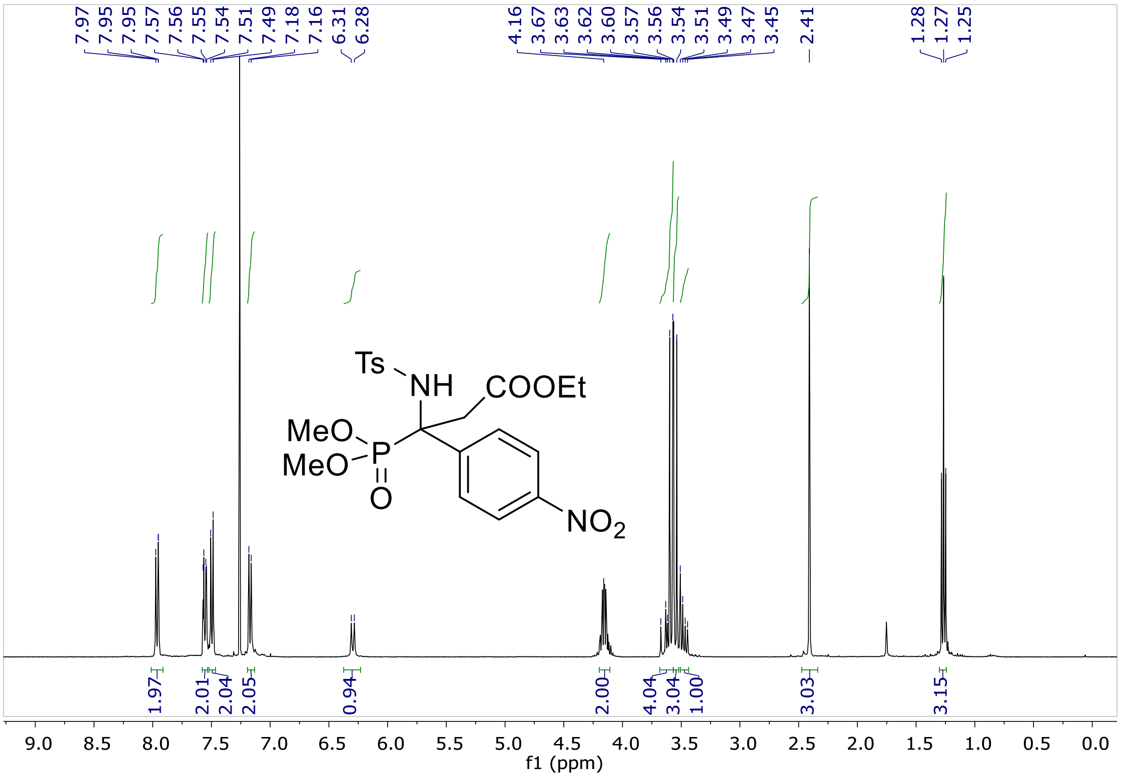

^{13}C NMR {^{1}H} (101 MHz, $CDCl_3$)

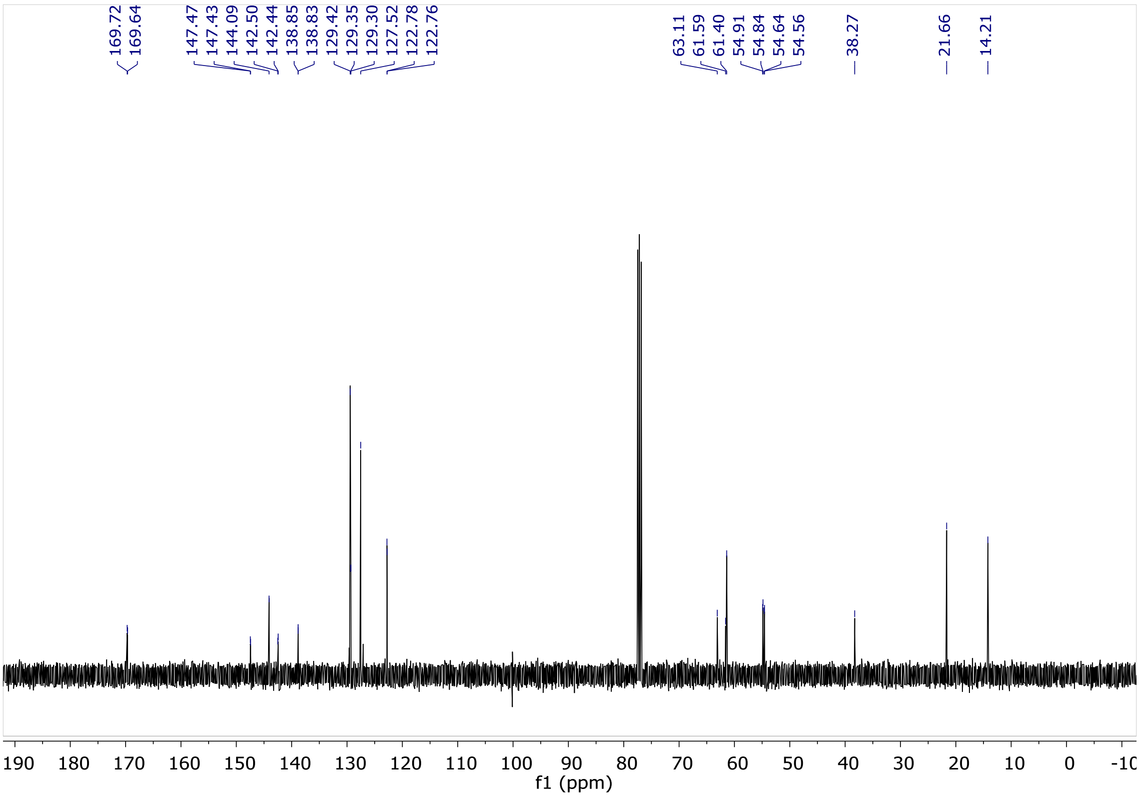

^{31}P NMR (120 MHz, $CDCl_3$)

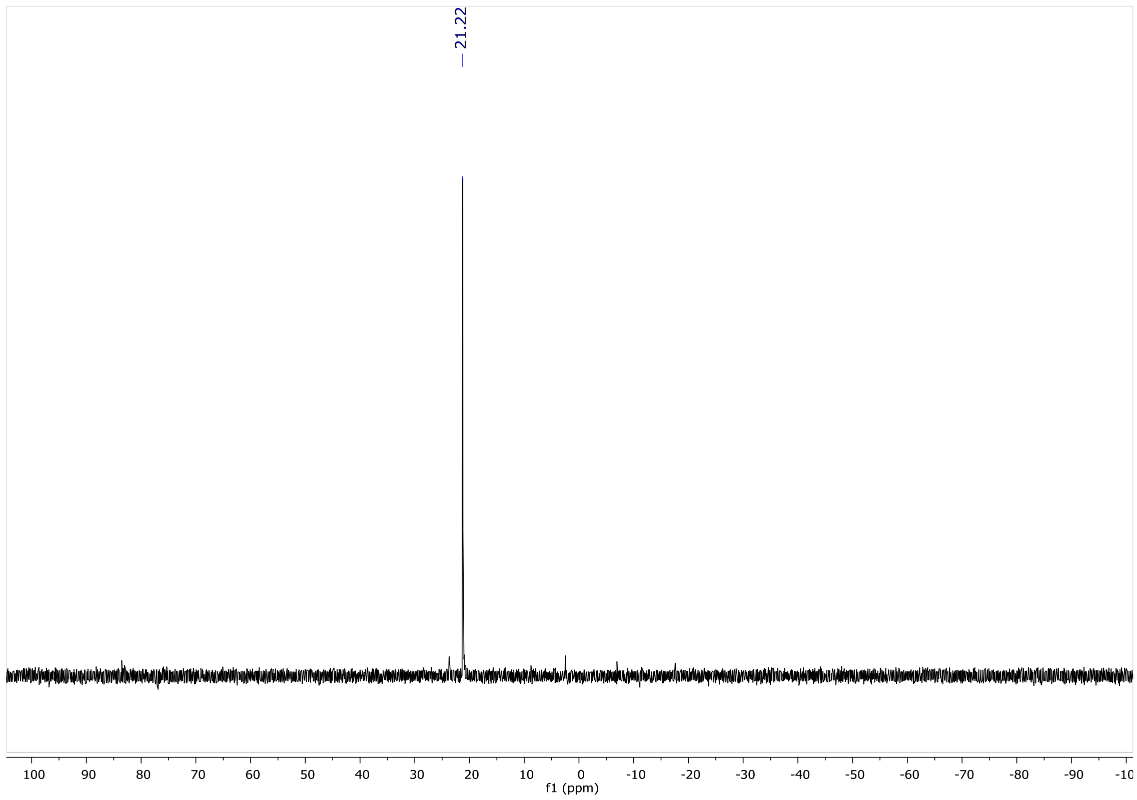

Ethyl 3-(5-chlorothiophen-2-yl)-3-(dimethoxyphosphoryl)-3-((4-methylphenyl)sulfonamido) propanoate (7r)

^{1}H NMR (400 MHz, $CDCl_3$)

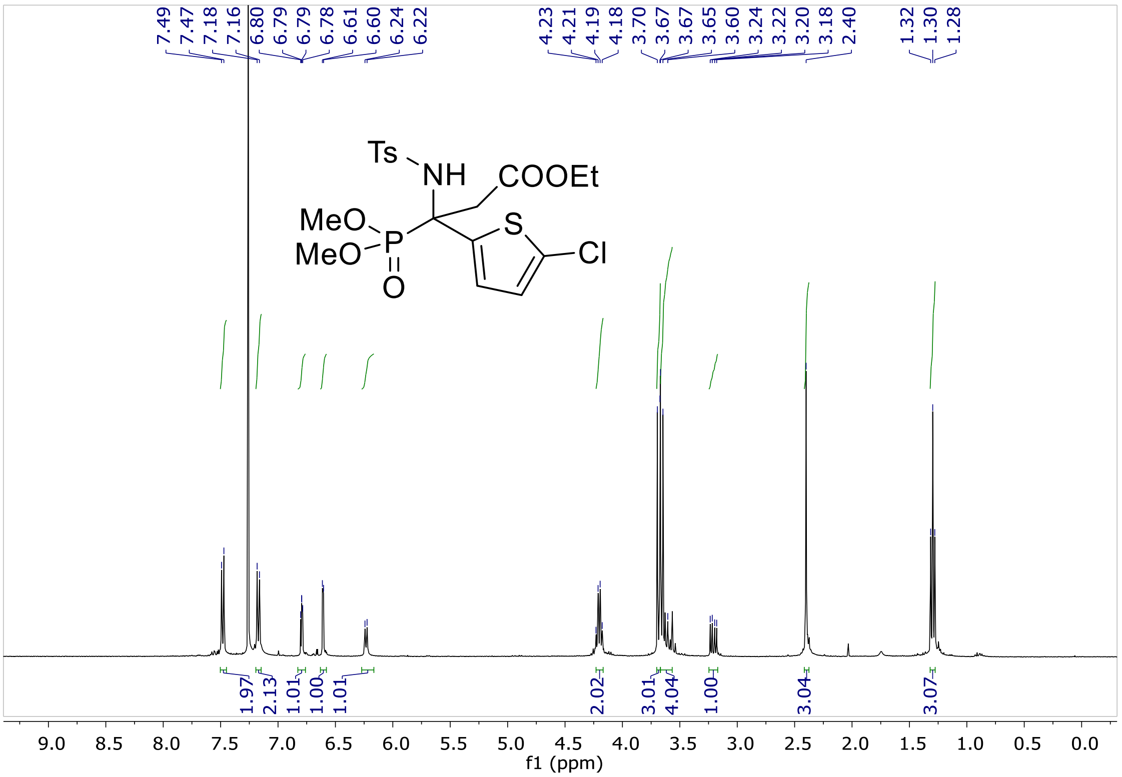

^{13}C NMR {^{1}H} (101 MHz, $CDCl_3$)

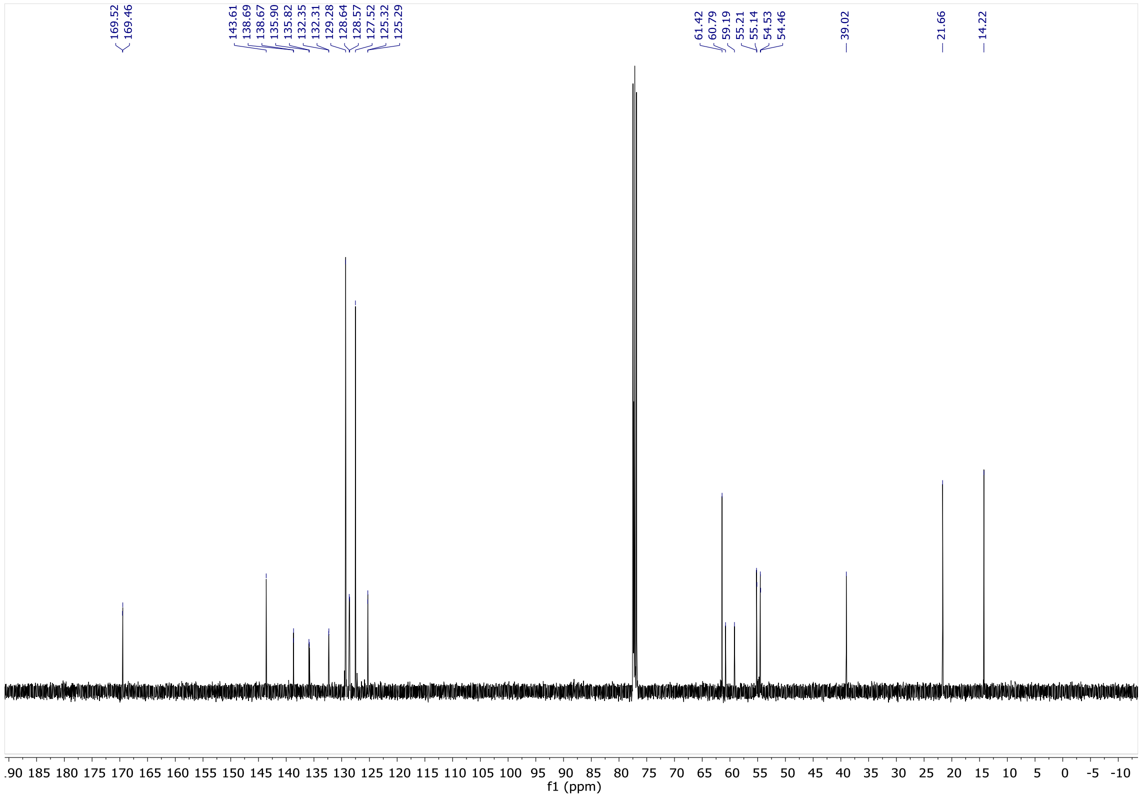

^{31}**P NMR** (120 MHz, $CDCl_3$)

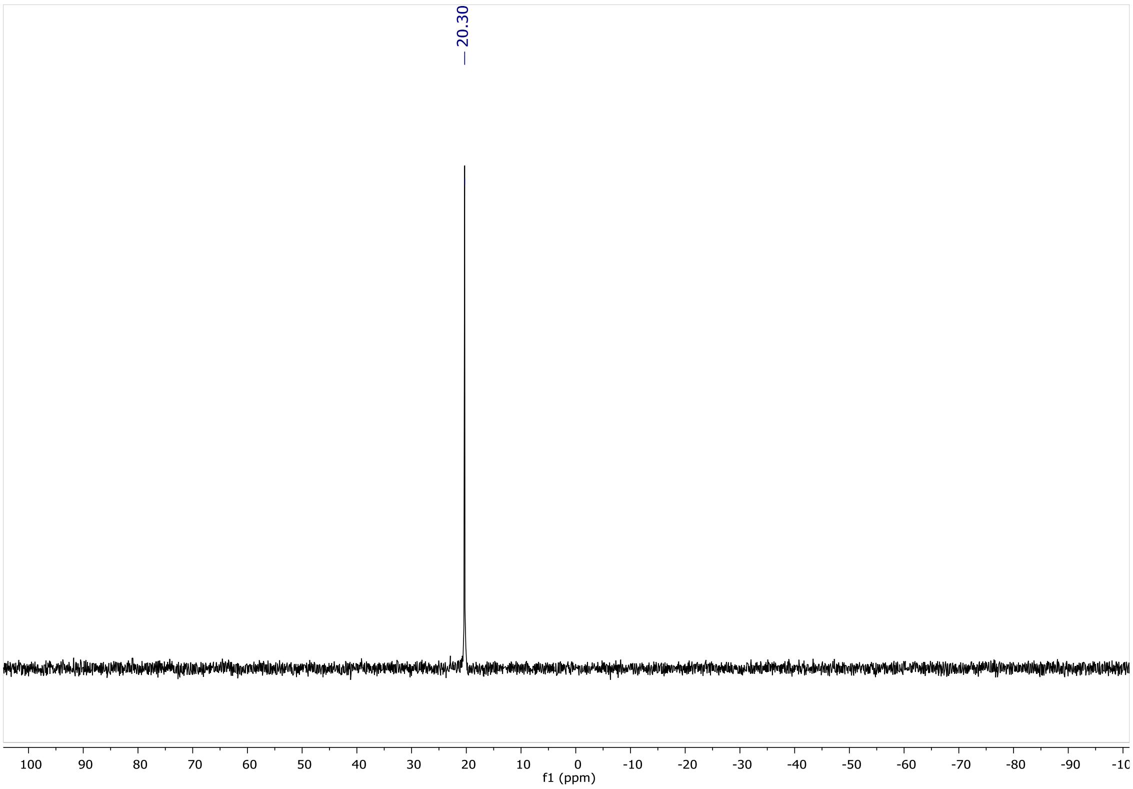

Ethyl 3-([1,1'-biphenyl]-4-yl)-3-(dimethoxyphosphoryl)-3-((4-methylphenyl)sulfonamido) propanoate (7s)

^{1}H NMR (400 MHz, $CDCl_3$)

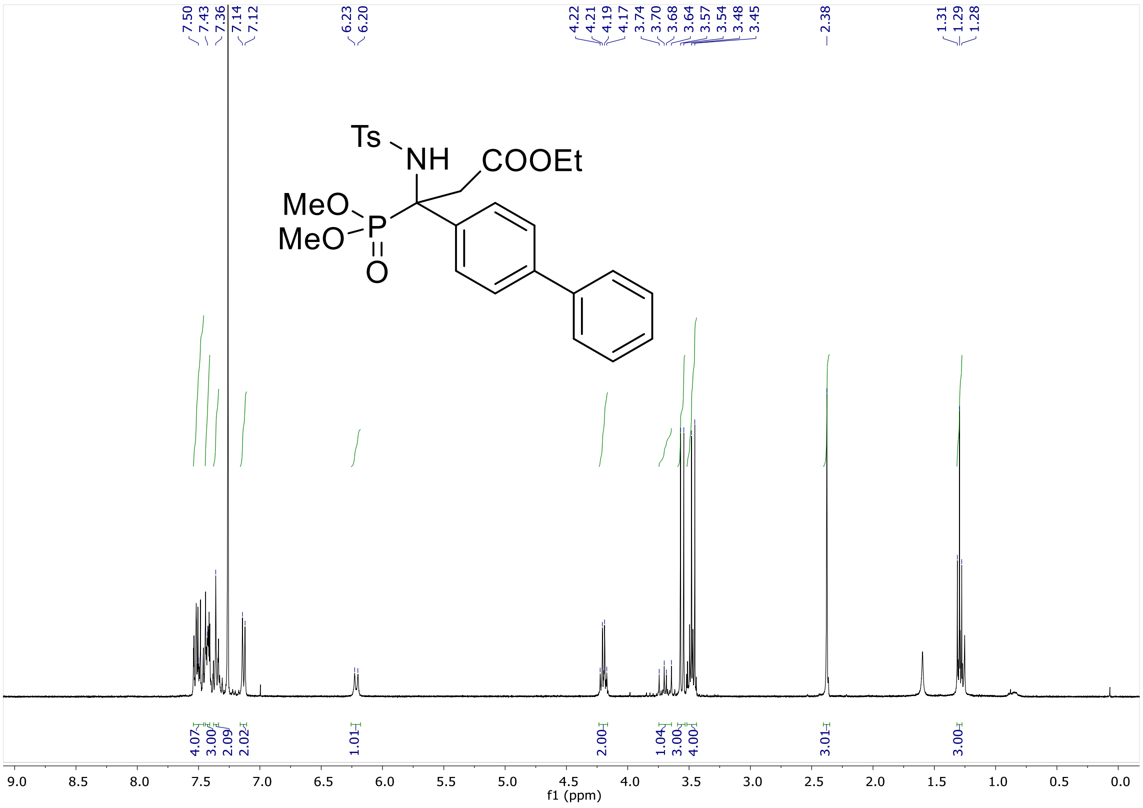

^{13}C NMR {^{1}H} (101 MHz, $CDCl_3$)

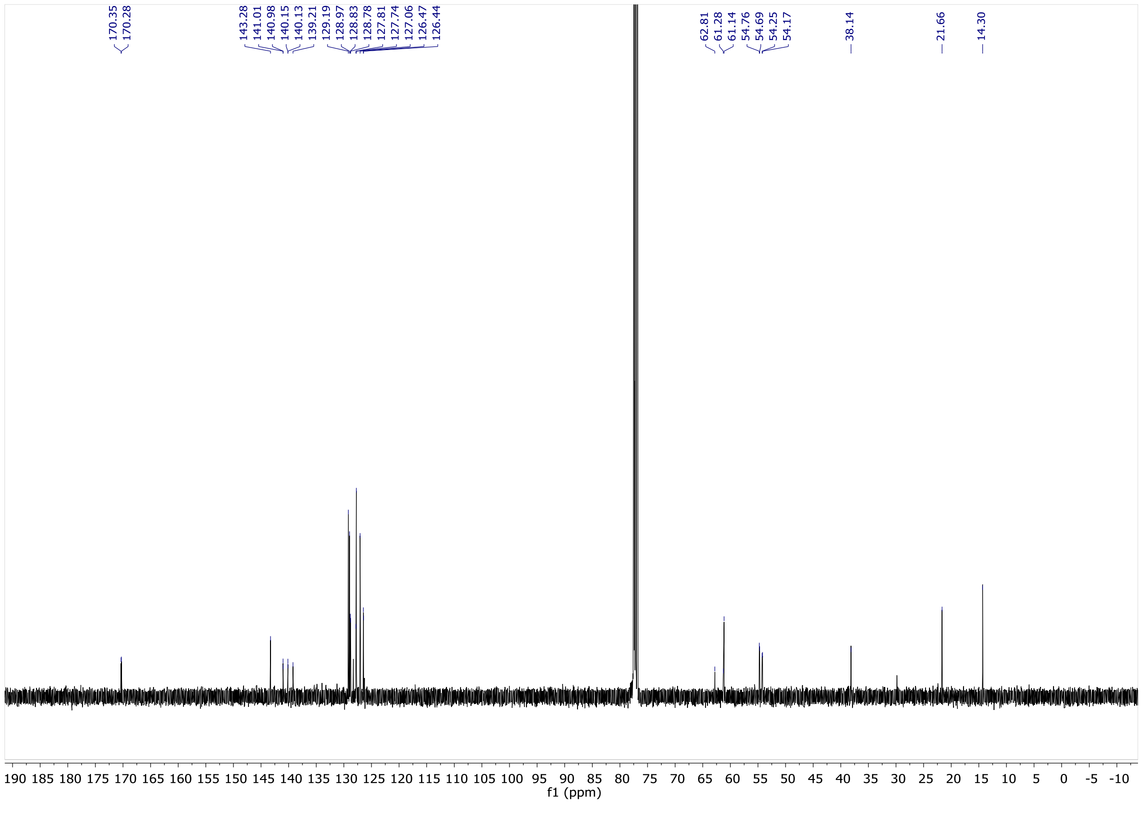

^{31}P NMR (120 MHz, $CDCl_3$)

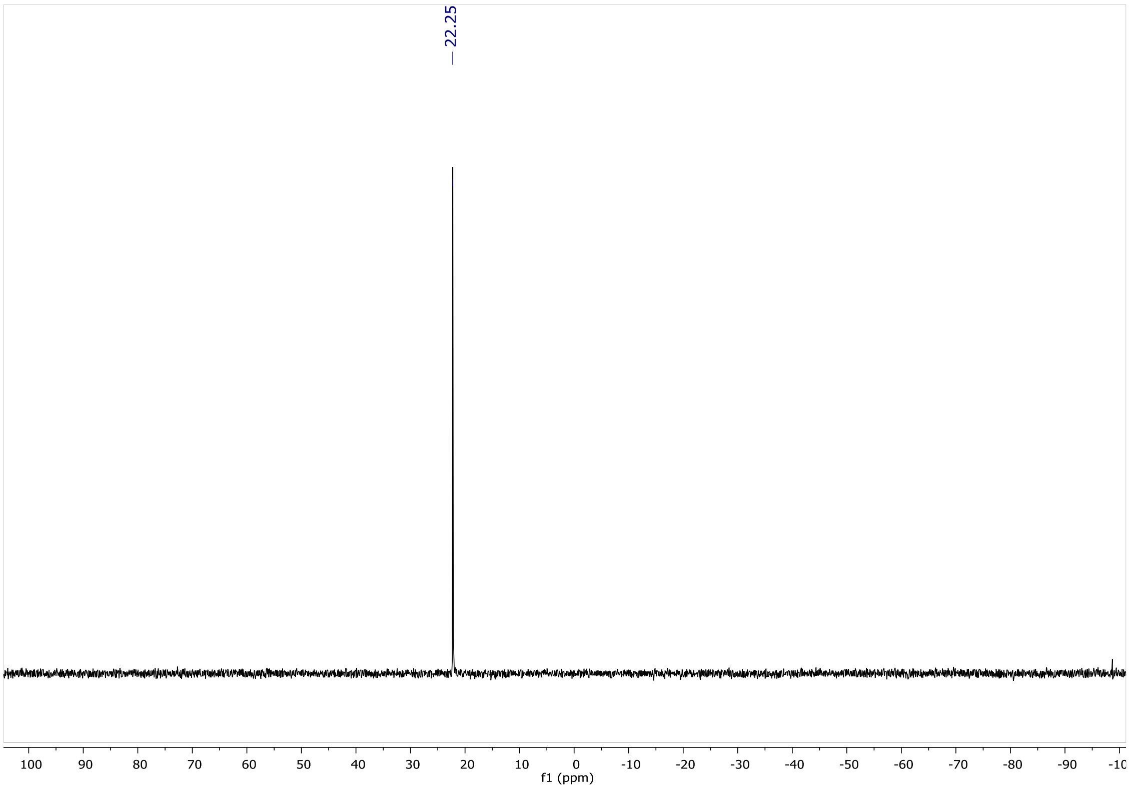

3. ^{1}H NMR, ^{13}C NMR, ^{31}P NMR and ^{19}FNMR spectra of compounds 12, 13 and 14

Methyl 3-(dimethoxyphosphoryl)-3-(3-fluorophenyl)-3-((4-methylphenyl)sulfonamido) propanoate (12)

^{1}**H NMR** (400 MHz, $CDCl_3$)

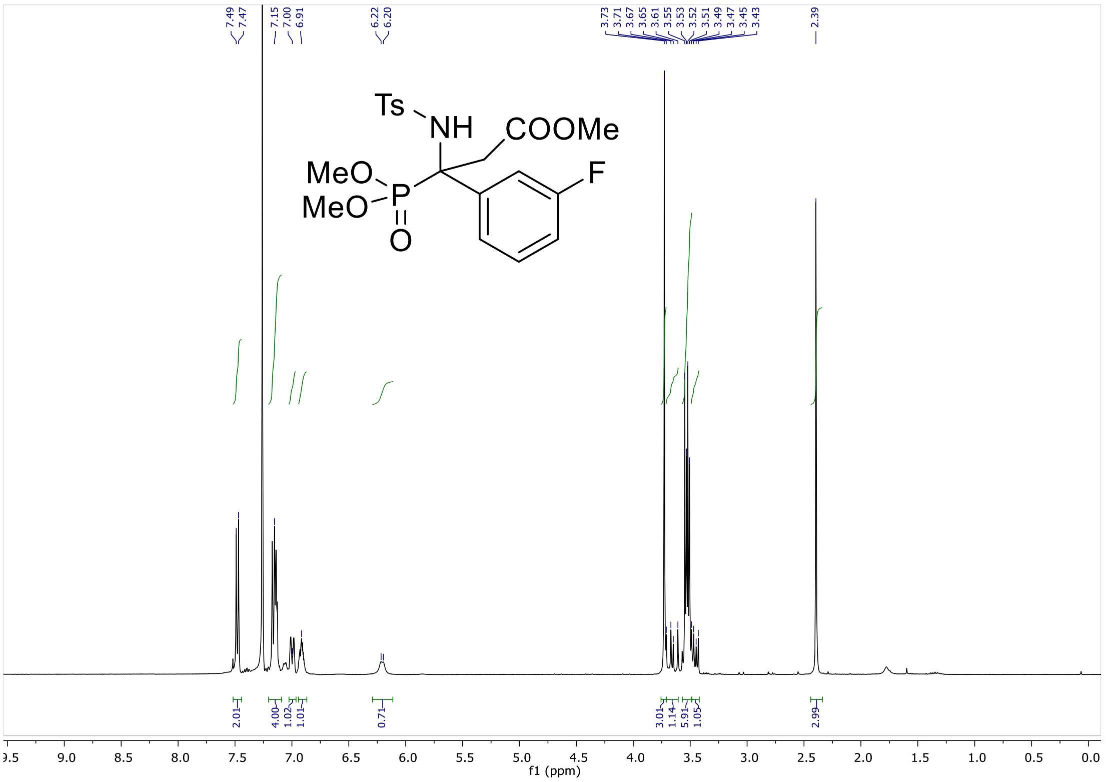

^{13}**C NMR** {^{1}H} (101 MHz, $CDCl_3$)

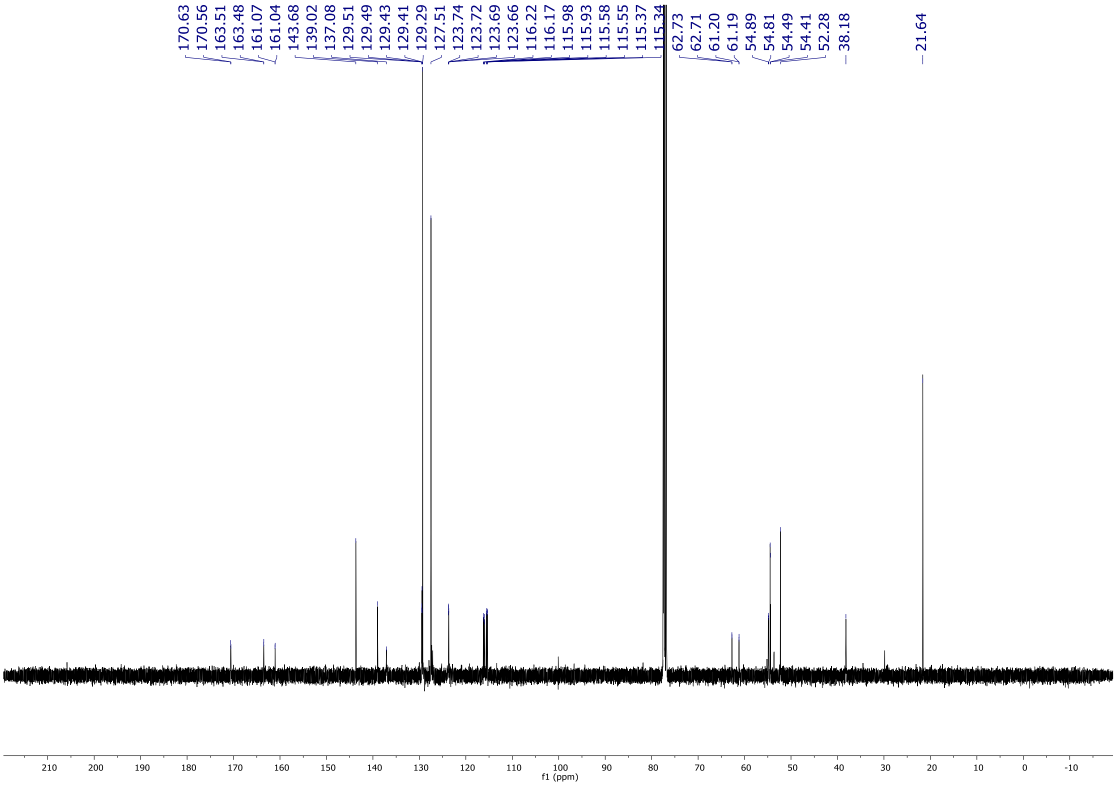

^{31}P NMR (120 MHz, $CDCl_3$)

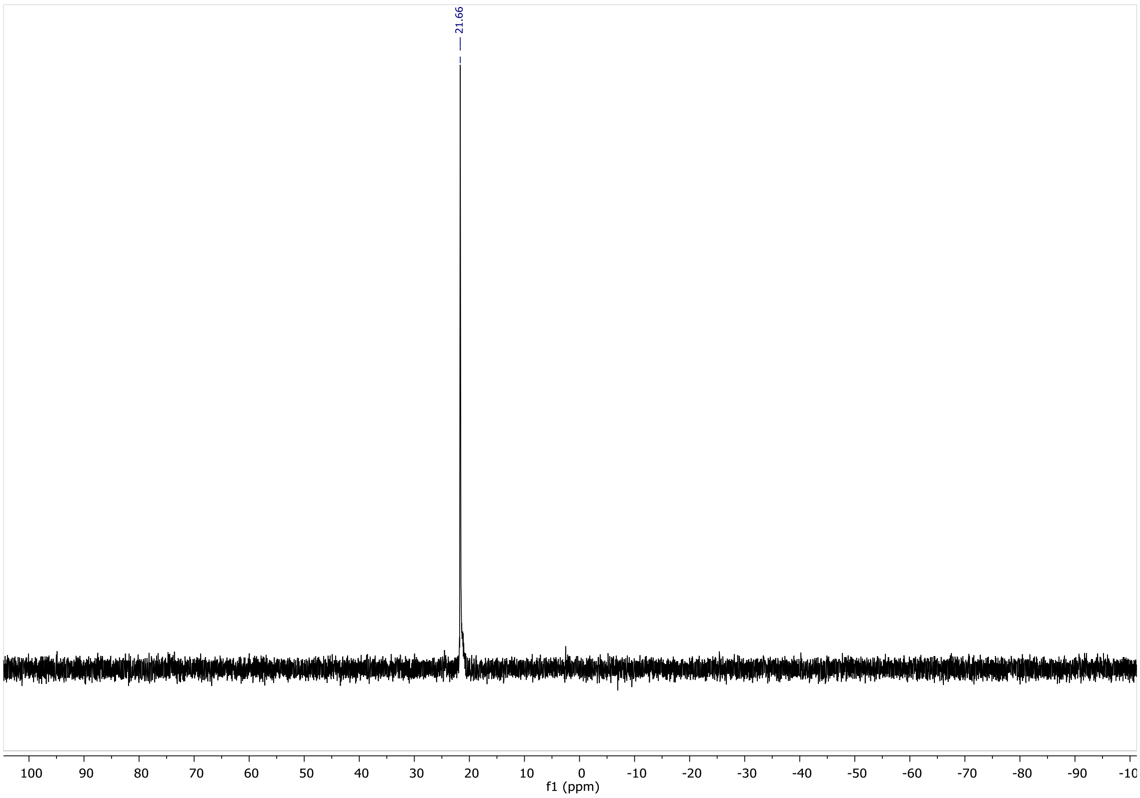

^{19}F NMR (282 MHz, $CDCl_3$)

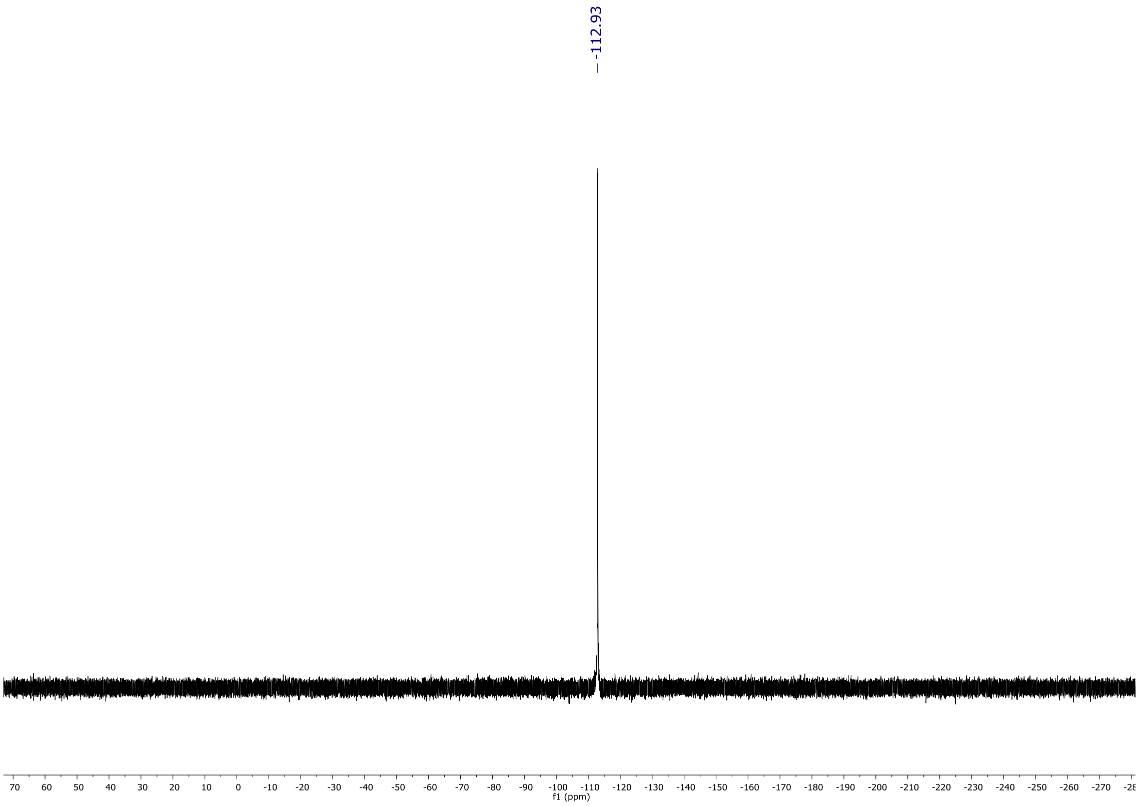

Benzyl 3-(dimethoxyphosphoryl)-3-(3-fluorophenyl)-3-((4-methylphenyl)sulfonamido) propanoate (13a)

^{1}H NMR (400 MHz, $CDCl_3$)

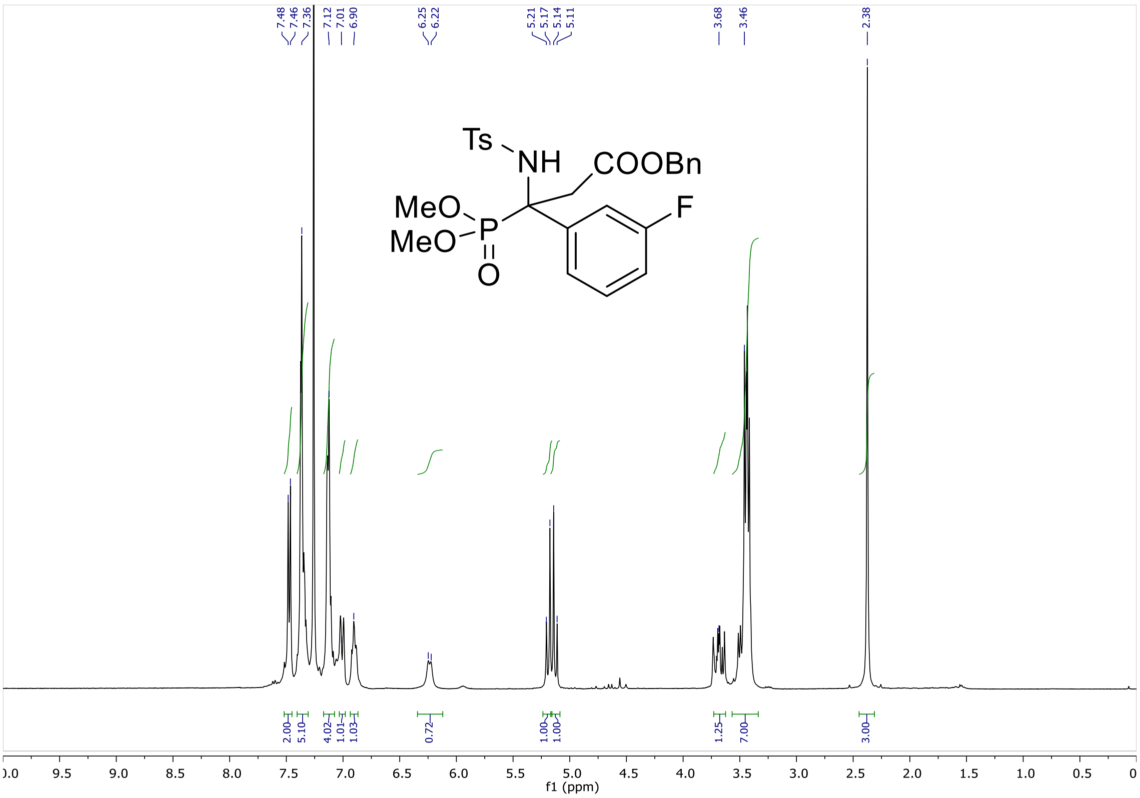

^{13}C NMR {^{1}H} (101 MHz, $CDCl_3$)

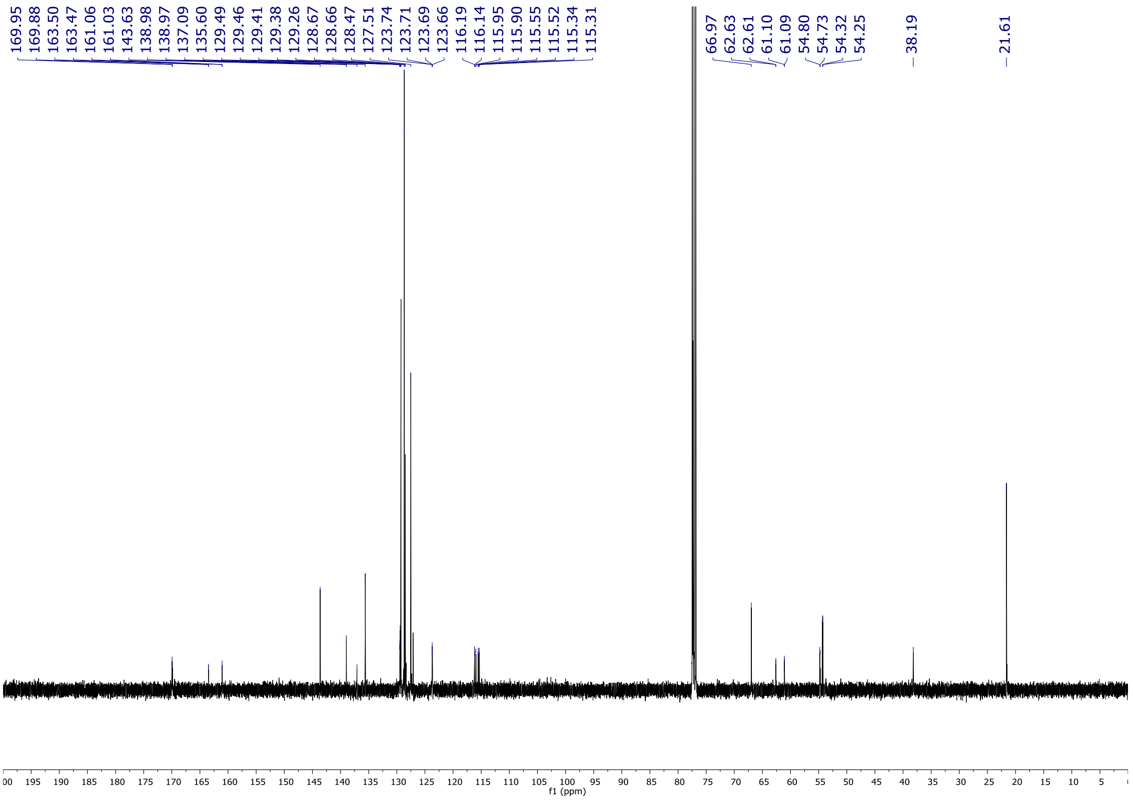

^{31}P NMR (120 MHz, $CDCl_3$)

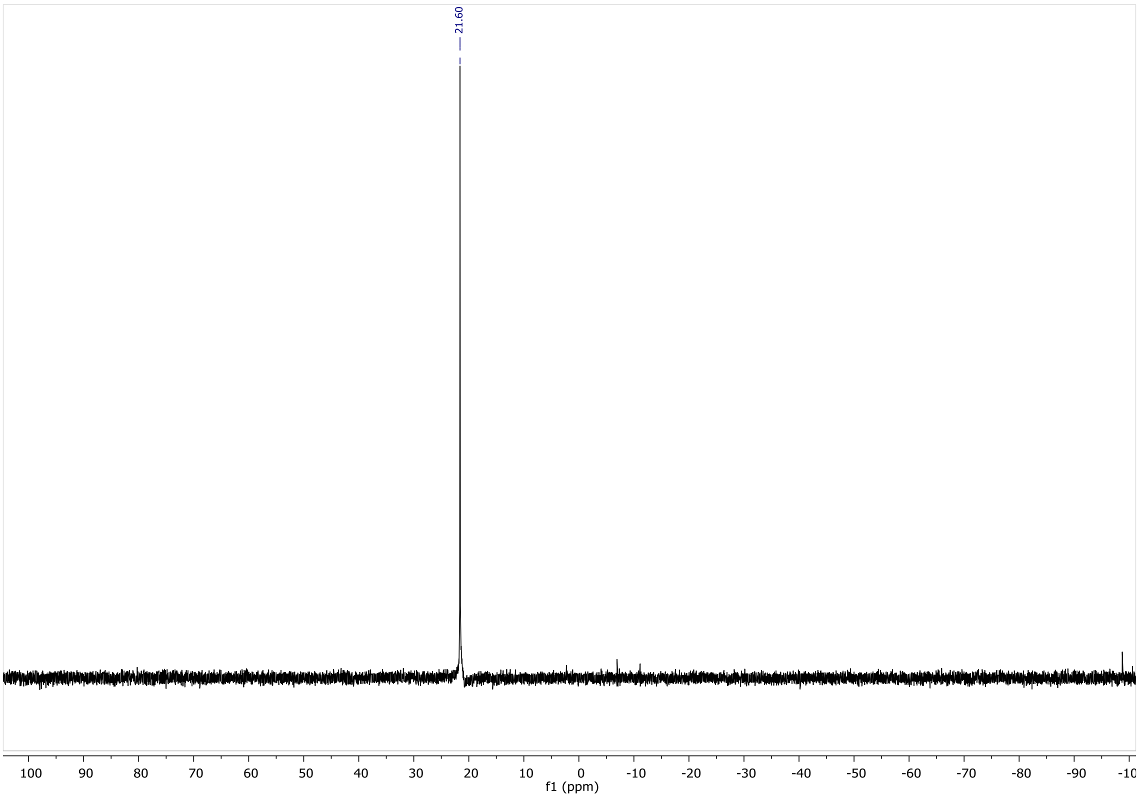

^{19}F NMR (282 MHz, $CDCl_3$)

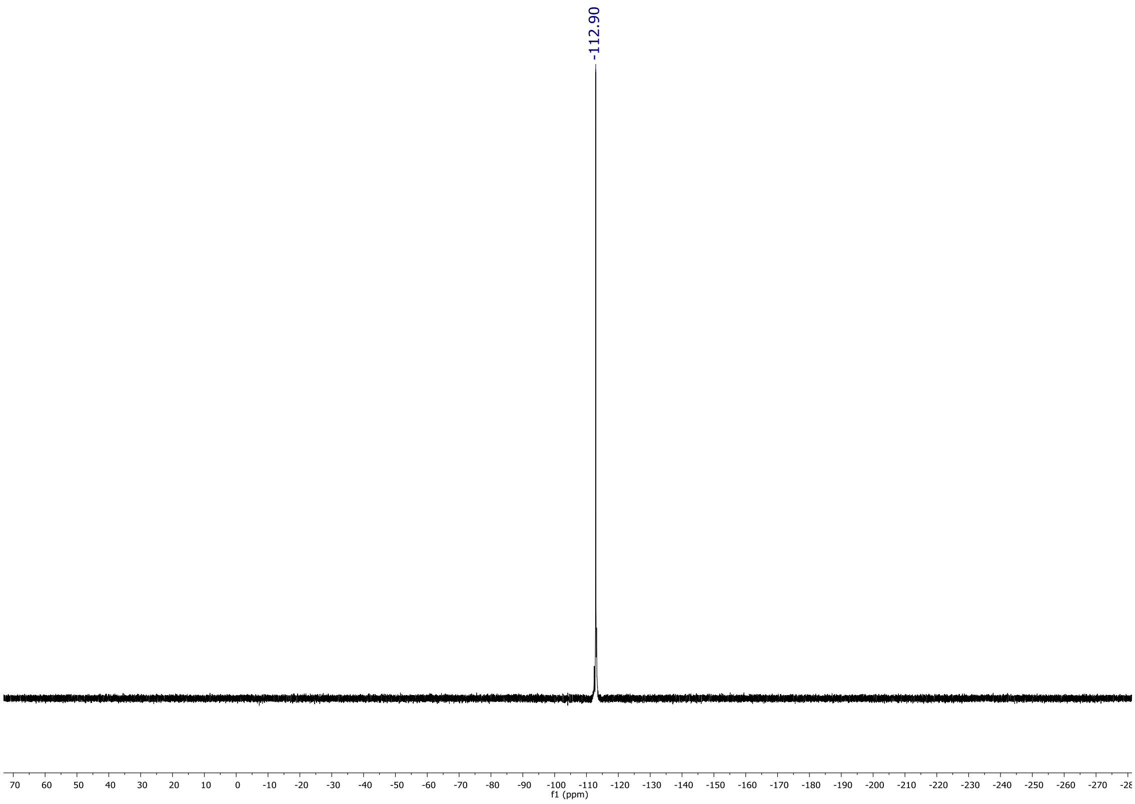

Benzyl 3-(dimethoxyphosphoryl)-3-((4-methylphenyl)sulfonamido)-3-phenylpropanoate (13b)

^{1}H NMR (400 MHz, $CDCl_3$)

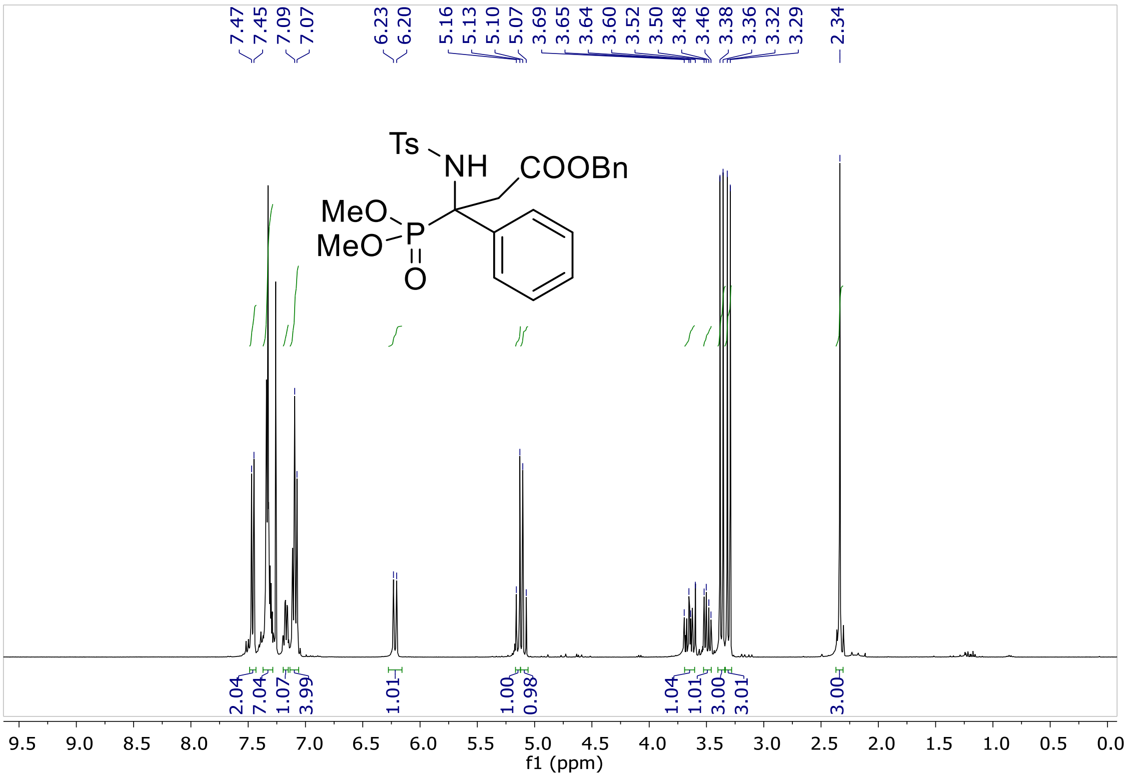

^{13}C NMR {^{1}H} (101 MHz, $CDCl_3$)

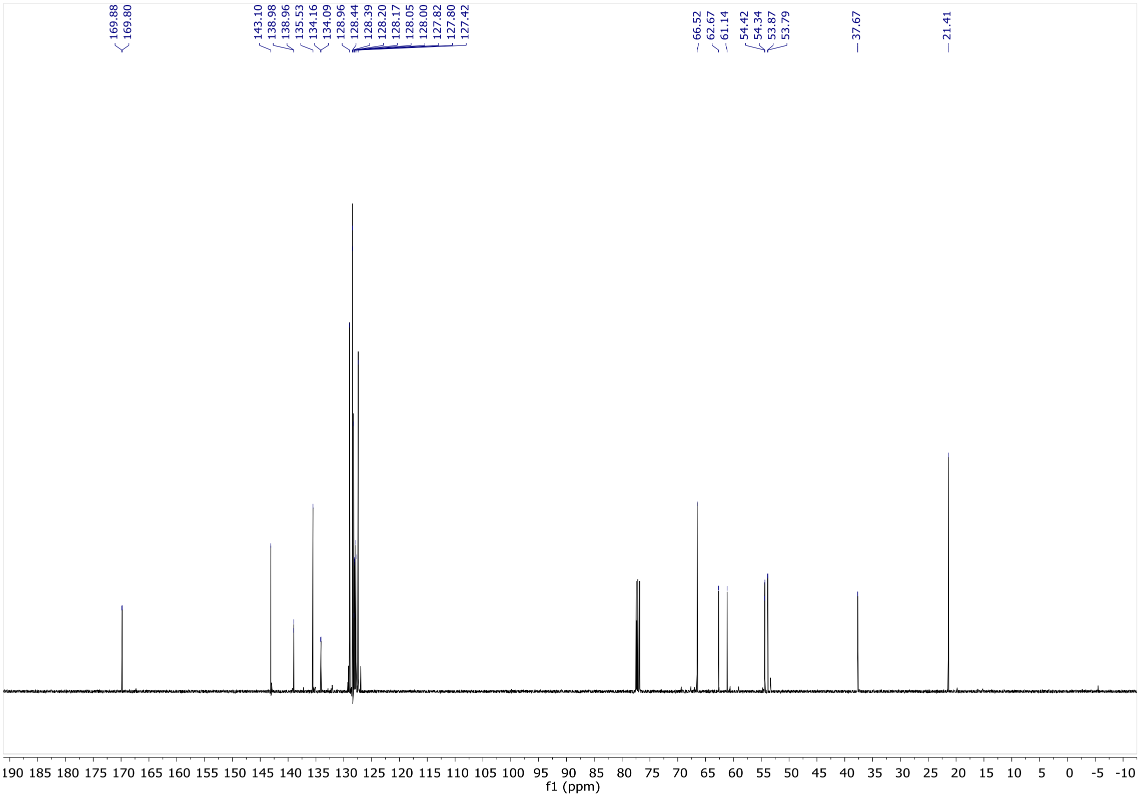

^{31}**P NMR** (120 MHz, $CDCl_3$)

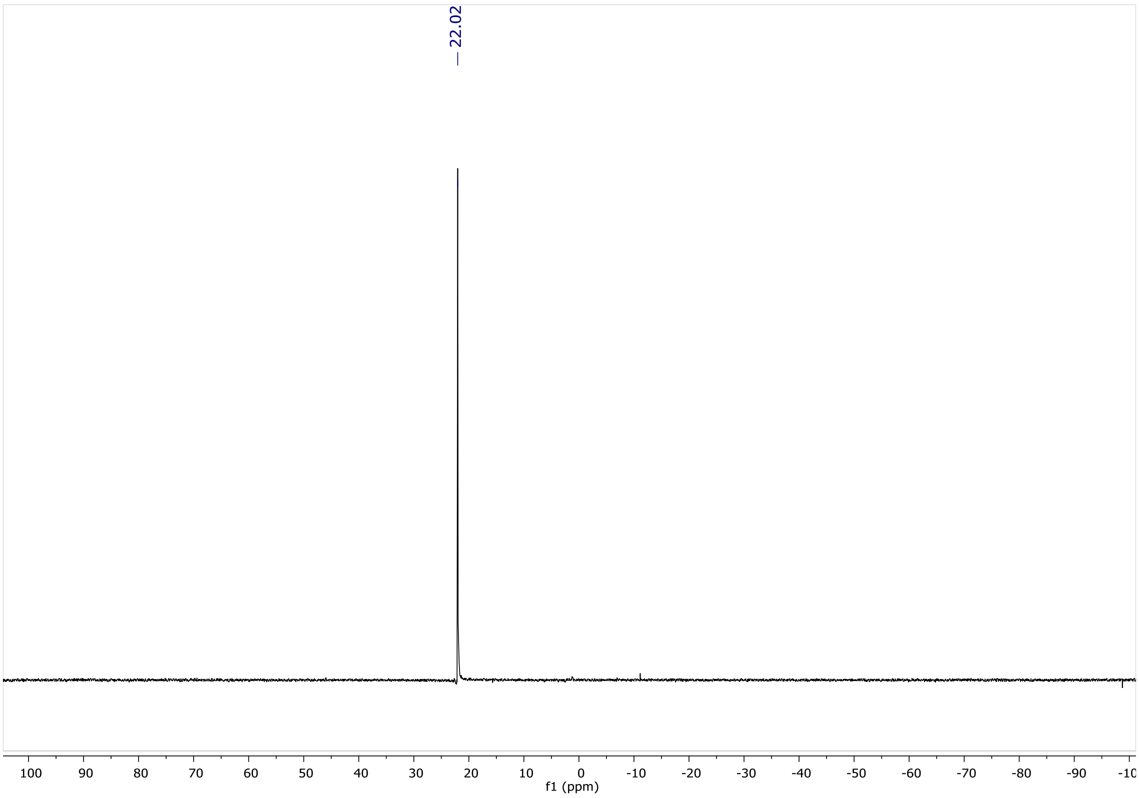

3-(Dimethoxyphosphoryl)-3-((4-methylphenyl)sulfonamido)-3-phenylpropanoic acid (14)

¹H NMR (400 MHz, $CDCl_3$)

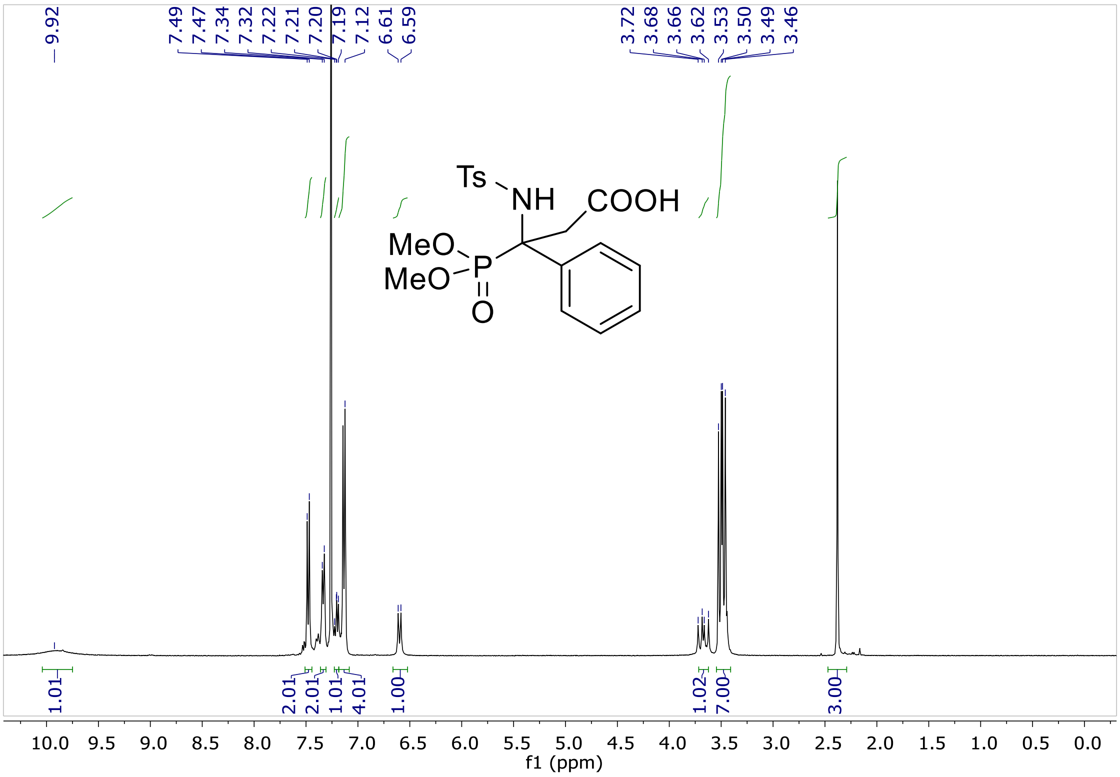

¹³C NMR {¹H} (101 MHz, $CDCl_3$)

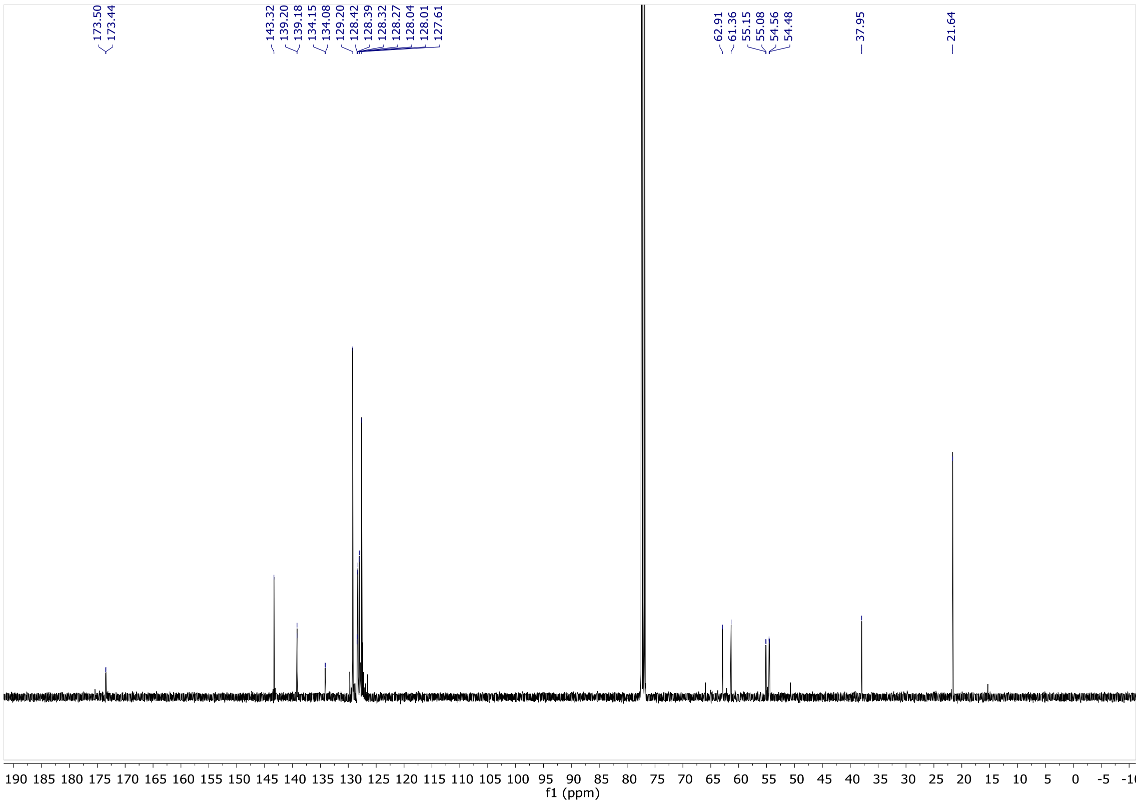

^{31}P NMR (120 MHz, $CDCl_3$)

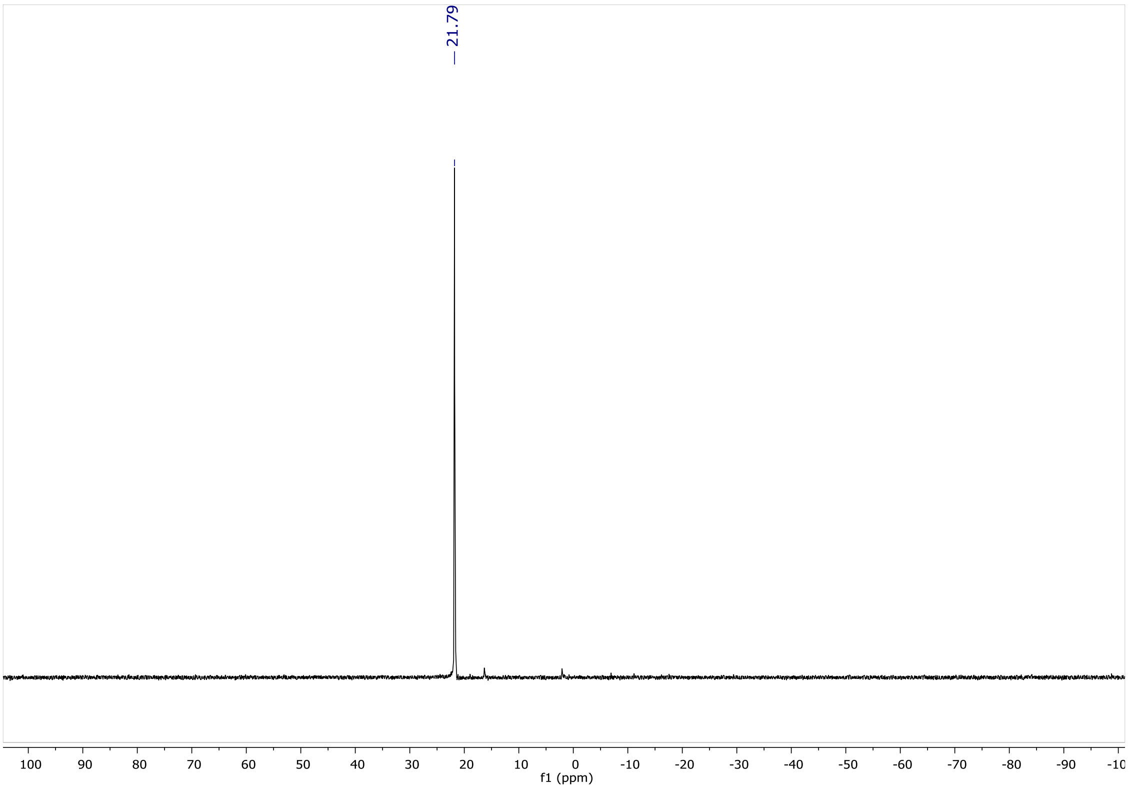

4. ¹H NMR, ¹³C NMR and ³¹P NMR spectra of compound 18

Dimethyl (1-((4-methylphenyl)sulfonamido)-1-phenylethyl)phosphonate (18)

¹H NMR (400 MHz, $CDCl_3$)

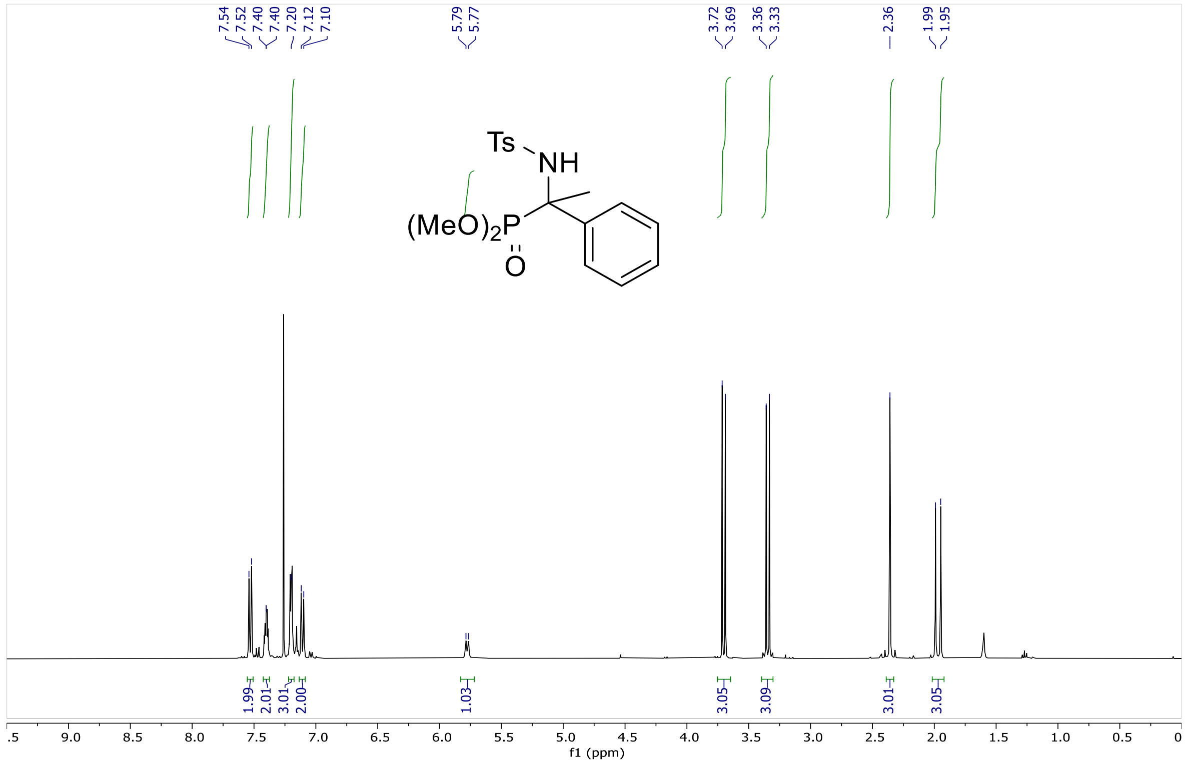

¹³C NMR {¹H} (75 MHz, $CDCl_3$)

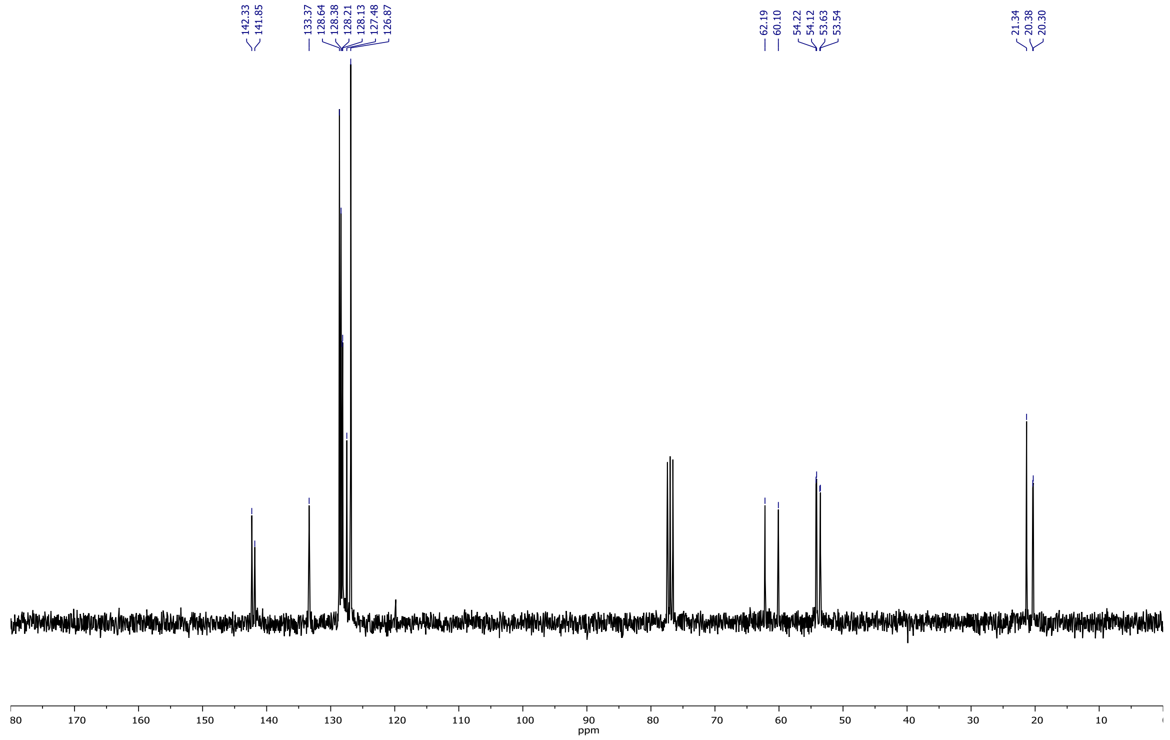

^{31}**P NMR** (120 MHz, $CDCl_3$)

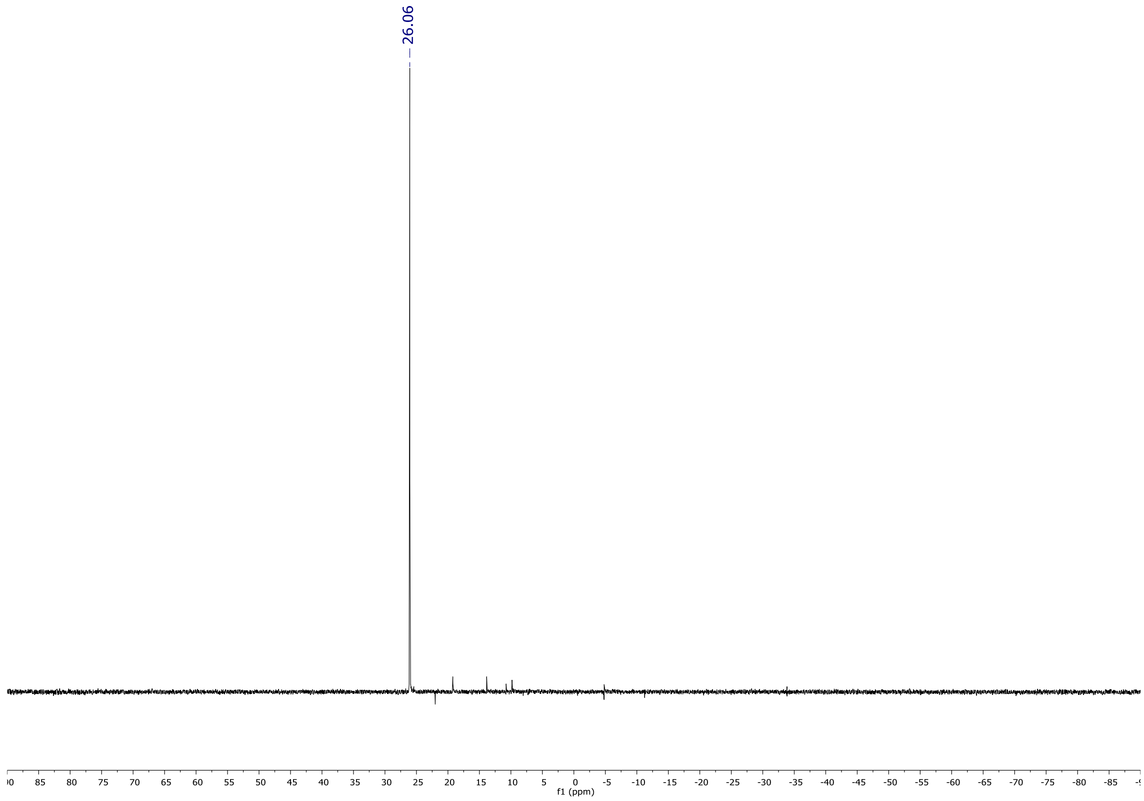

5. 2D NMR spectra of compound 7a

2D-COSY NMR {^1H – ^1H} (400 MHz, $CDCl_3$)

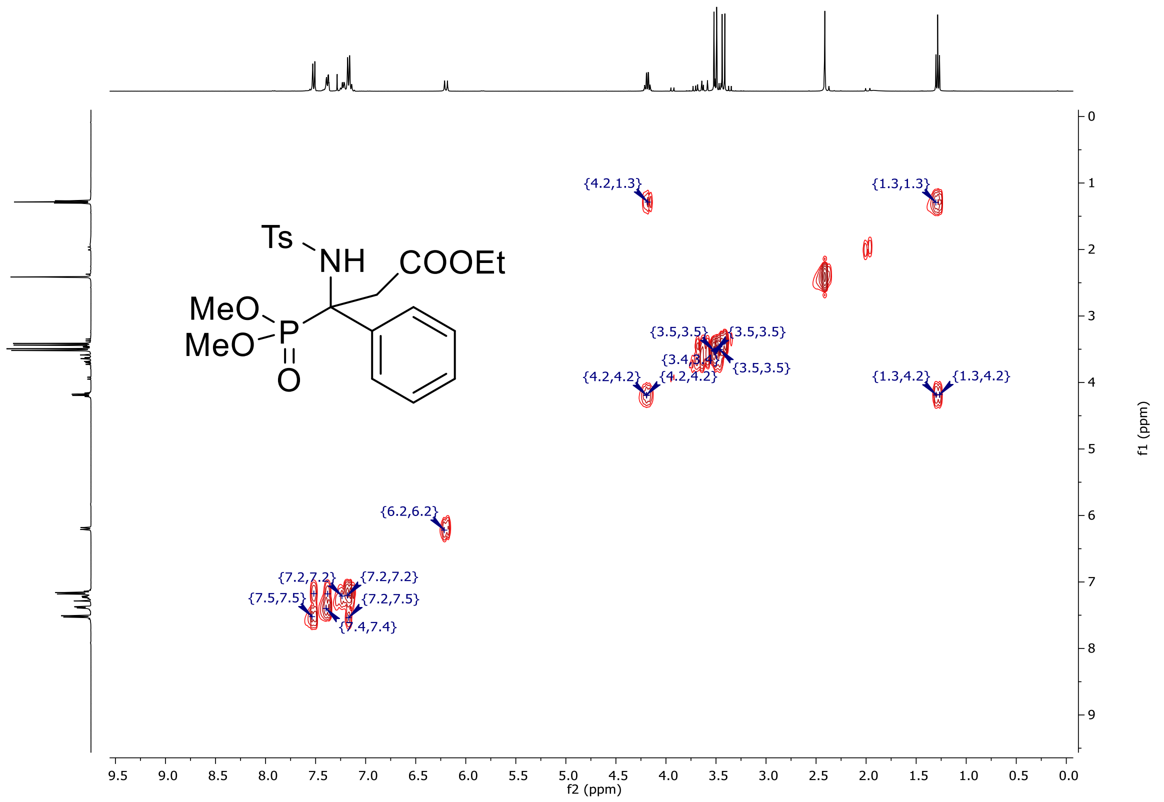

2D-HSQC NMR {^1H – ^{13}C} (^1H: 400 MHz, ^{13}C: 101 MHz, $CDCl_3$)

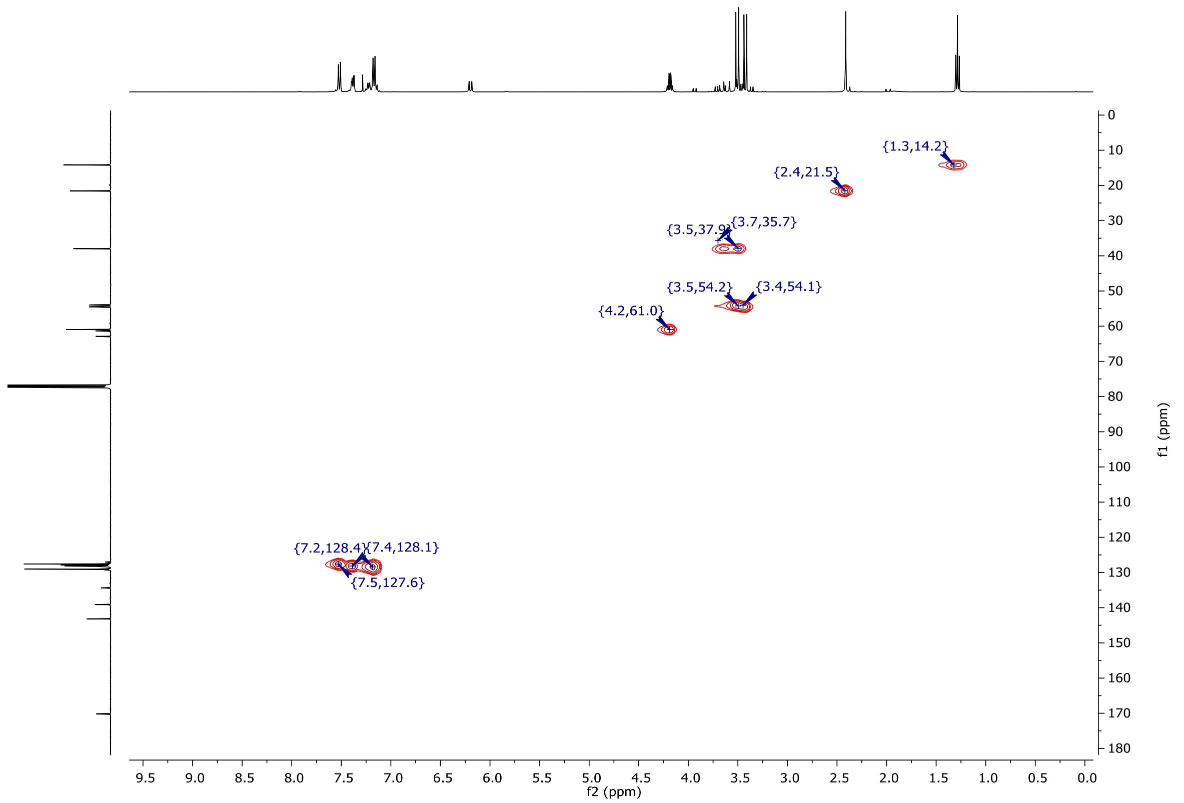

2D-HMBC NMR {^{1}H – ^{13}C} (^{1}H: 400 MHz, ^{13}C: 101 MHz, $CDCl_3$)

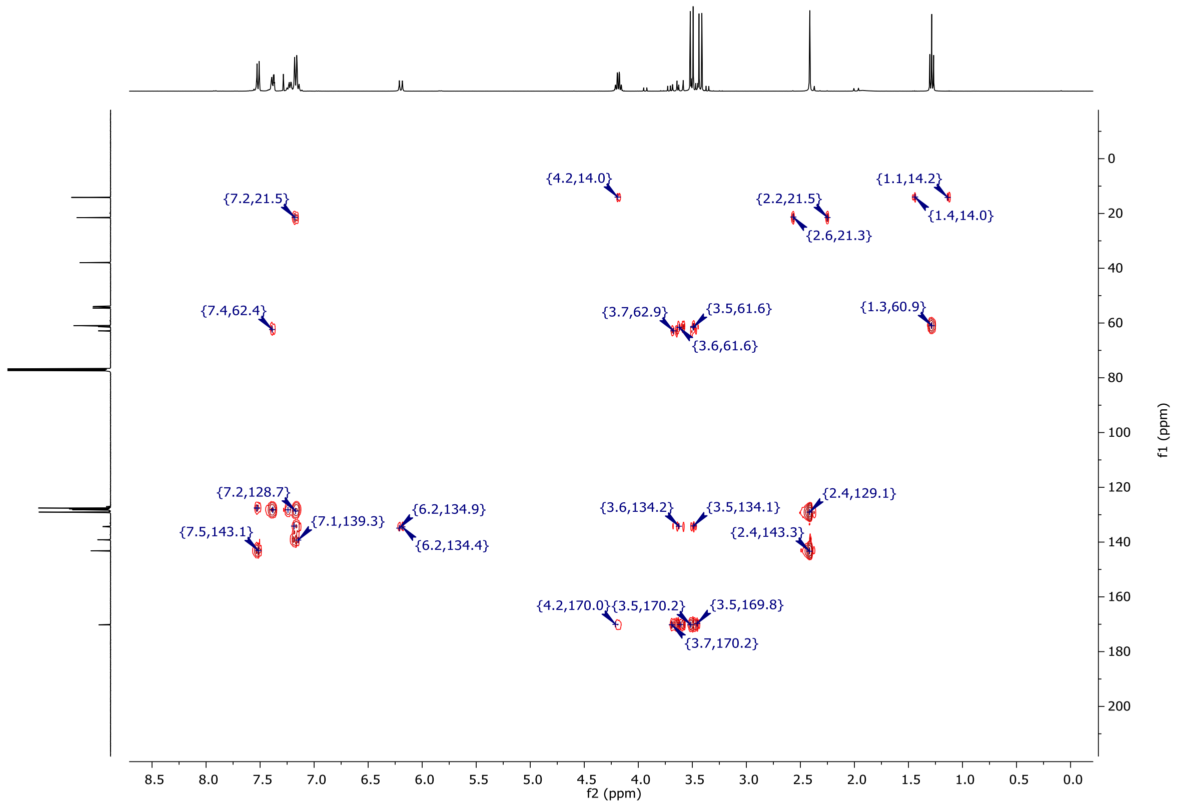

Supplement: Supplementary file 1 [file molecules-27-08024-s001.zip › molecules-1948083-supplementary.pdf]
